# Doubly periodic lozenge tilings of a hexagon and matrix valued orthogonal polynomials

**DOI:** 10.1111/sapm.12339

**Published:** 2020-10-02

**Authors:** Christophe Charlier

**Affiliations:** ^1^ Department of Mathematics Royal Institute of Technology (KTH) Stockholm Sweden

**Keywords:** doubly periodic lozenge tilings, matrix valued orthogonal polynomials, Riemann–Hilbert problems

## Abstract

We analyze a random lozenge tiling model of a large regular hexagon, whose underlying weight structure is periodic of period 2 in both the horizontal and vertical directions. This is a determinantal point process whose correlation kernel is expressed in terms of non‐Hermitian matrix valued orthogonal polynomials (OPs). This model belongs to a class of models for which the existing techniques for studying asymptotics cannot be applied. The novel part of our method consists of establishing a connection between matrix valued and scalar valued OPs. This allows to simplify the double contour formula for the kernel obtained by Duits and Kuijlaars by reducing the size of a Riemann–Hilbert problem. The proof relies on the fact that the matrix valued weight possesses eigenvalues that live on an underlying Riemann surface M of genus 0. We consider this connection of independent interest; it is natural to expect that similar ideas can be used for other matrix valued OPs, as long as the corresponding Riemann surface M is of genus 0. The rest of the method consists of two parts, and mainly follows the lines of a previous work of Charlier, Duits, Kuijlaars and Lenells. First, we perform a Deift–Zhou steepest descent analysis to obtain asymptotics for the scalar valued OPs. The main difficulty is the study of an equilibrium problem in the complex plane. Second, the asymptotics for the OPs are substituted in the double contour integral and the latter is analyzed using the saddle point method. Our main results are the limiting densities of the lozenges in the disordered flower‐shaped region. However, we stress that the method allows in principle to rigorously compute other meaningful probabilistic quantities in the model.

## 1 INTRODUCTION

**FIGURE 1:**
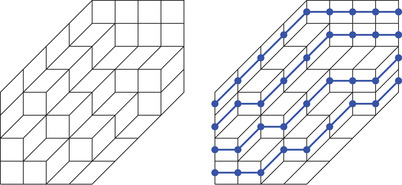
A tiling of a hexagon, and the associated non‐intersecting paths

A lozenge tiling of a hexagon is a collection of three different types of lozenges (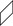, 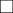, and 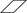), which cover this hexagon without overlaps, see Figure 1 (left). There are finitely many such tilings; hence by assigning to each tiling T a nonnegative weight W(T), we define a probability measure on the tilings by

(1) $$\begin{array}{c} P\left(T\right)=\frac{W(T)}{\sum_{T^{\prime }}W\left(T^{\prime }\right)}, \end{array}$$

where the sum is taken over all the tilings (and is assumed to be nonzero). Uniform random tilings of a hexagon (i.e., when W(T)=1 for all T) is a well‐studied model. As the size of the hexagon tends to infinity (while the size of the lozenges is kept fixed), the local statistical properties of this model are described by universal processes.^1^, ^2^, ^3^, ^4^ We also refer to Refs. 5, 6, 7 for important early results and to Refs. 8, 9 for general references on tiling models. Uniform lozenge tilings of more complicated domains (nonnecessarily convex) have also been widely studied in recent years.^10^, ^11^, ^12^, ^13^, ^14^

**FIGURE 2:**
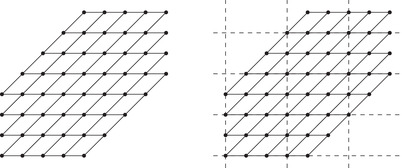
The graph $G_{4}$, and the periods of a 2 × 3 periodic weighting

In this work, we consider the regular hexagon of (large) size *n*

(2) $$\begin{array}{c} H_{n}:=\left\{\left(x,y\right)\in R^{2}:0\leq x\leq 2n,0\leq y\leq 2n,-n\leq x-y\leq n\right\},\;n\in N_{\geq 1}, \end{array}$$

but we deviate from the uniform measure and study instead measures with periodic weightings. To explain what this means, we first briefly recall a well‐known one‐to‐one correspondence between tilings of a hexagon and certain nonintersecting paths. This bijection can be written down explicitly, but is best understood informally. The paths are obtained by drawing lines on top of two types of lozenges
(3)
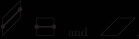
as shown in Figure 1 (right). The paths associated to the tilings of $H_{n}$ lie on a graph $G_{n}$, which depends only on the size of the hexagon, see Figure 2 (left). To each edge e of $G_{n}$, we assign a nonnegative weight $w_{e}$. The weight of a path p is then defined as $w_{p}=\prod_{e\in p}w_{e}$, and the weight of a tiling T as $W\left(T\right)=\prod_{p\in T}w_{p}$. Provided that at least one tiling has a positive weight, this defines a probability measure on the set of tilings by (1). If each edge is assigned the same weight, then we recover the uniform measure over tilings. We say that a lozenge tiling model has p×q periodic weightings if the weight structure on the edges is periodic of period *p* in the vertical direction, and periodic of period *q* in the horizontal direction, see Figure 2 (right) for an illustration with p=2 and q=3. Thus, a p×q periodic weighting is completely determined by 2pq edge weights. Note that all paths share the same number of horizontal edges, and also the same number of oblique edges; hence lozenge tiling models with 1 × 1 periodic weightings are all equivalent to the uniform measure.

By putting points on the paths as shown in (3), each tiling of the hexagon gives rise to a point configuration, see also Figure 1 (right). Thus, the probability measure (1) on tilings can be viewed as a discrete point process.^15^, ^16^ For lozenge tiling models with p×q periodic weightings, it follows from the Lindström‐Gessel‐Viennot theorem^17^, ^18^ combined with the Eynard‐Mehta theorem^19^ that this point process is determinantal. Therefore, to understand the fine asymptotic structure (as n→+∞) it suffices to analyze the asymptotic behavior of the correlation kernel. However, until recently,^20^, ^21^ the existing techniques were not appropriate for such analysis.

The main result of Ref. 20 is a double contour formula for the correlation kernels of various tiling models with periodic weightings (including lozenge tiling models of a hexagon as considered here). In this formula, the integrand is expressed in terms of the solution (denoted *Y*) to a 2p×2p Riemann–Hilbert (RH) problem. This RH problem is related to certain orthogonal polynomials (OPs), which are nonstandard in two aspects:

- the OPs and the weight are p×p matrix valued,
- the orthogonality conditions are non‐Hermitian.

The size of the RH problem, the size of the weight, and the size of the OPs all depend on *p*, but quite interestingly not on *q*.

Lozenge tiling models of the hexagon with p×q periodic weightings are rather unexplored up to now. To the best of our knowledge, the model considered in Ref. 21 is the only one (other than the uniform measure) prior to the present work for which results on fine asymptotics exist. The model considered in Ref. 21 is 1 × 2 periodic and uses the formula of Ref. 20 as the starting point of the analysis. The techniques of Ref. 21 combine the Deift/Zhou steepest descent method^22^ of *Y* (of size 2 × 2) with a nonstandard saddle point analysis of the double contour integral. However, because p=1, the associated OPs are scalar (this fact was extensively used in the proof) and it is not clear how to generalize these techniques to the case p≥2.

The aim of this paper is precisely to develop a method to handle a situation involving matrix valued OPs. We will implement this method on a particular lozenge tiling model with 2 × 2 periodic weightings, which presents one simply connected liquid region (which has the shape of a flower with six petals), six frozen regions, and six staircase regions (also called semifrozen regions); it will be presented in more detail in Section 2. The starting point of our analysis is the double contour formula from,^20^ which expresses the kernel in terms of a 4 × 4 RH problem related to 2 × 2 matrix valued OPs. The method can be summarized in three steps as follows:

1. First, we establish a connection between matrix valued and scalar valued OPs. In particular, in Theorem 1 we obtain a new expression for the kernel in terms of the solution (denoted *U*) to 2 × 2 RH problem related to scalar OPs. This formula allows for a simpler analysis than the original formula from Ref. 20.
2. Second, we perform an asymptotic analysis of the RH problem for *U* via the Deift–Zhou steepest descent method. The construction of the equilibrium measure and the associated *g*‐function is the main difficulty. The remaining part of the RH analysis is rather standard.
3. Third, the asymptotics for the OPs are substituted in the double contour integral and the latter is analyzed using the saddle point method.

The first step is the main novel part of the paper. The remaining two steps were first developed in Ref. 21 for a tiling model with 1 × 2 periodic weightings.

Our main results, which are stated in Theorem 2, are the limiting densities of the different lozenges in the liquid region. However, we emphasize that the method also allows in principle to rigorously compute more sophisticated asymptotic behaviors in the model (such as the limiting process in the bulk).

### 1.1 An expression for the kernel in terms of scalar OPs

The eigenvalues and eigenvectors of the 2 × 2 orthogonality weight play an important role in the first step of the analysis. They are naturally defined on a two‐sheeted Riemann surface M, which turns out to be of genus 0. This fact is crucial to obtain the new formula for the kernel in terms of scalar OPs. We expect that ideas similar to the ones presented here can be applied to other tiling models with periodic weightings, as long as the corresponding Riemann surface M is of genus 0.

Lozenge tiling models of large hexagons with periodic weightings can feature all of the three possible types of phases known in random tiling models: the solid, liquid, and gas phases. A solid region (also called frozen region) is filled with one type of lozenges. In the liquid and gas phases, all three types of lozenges coexist. The difference between these two phases is reflected in the correlations between two points: in the liquid region, the correlation decay is polynomial with the distance between the points, while in the gas region the decay is exponential. It is known that there is no gas phase for the uniform measure (corresponding to p=q=1). In fact, it is expected that the smallest periods that lead to the presence of a gas phase are either p=2,q=3 or p=3,q=2. For models that present gas phases, we expect M to have genus at least 1, and then new techniques are required. This is left for future works.

### 1.2 Related works

Random lozenge tilings of the regular hexagon is a particular example of a tiling model. We briefly review here other tiling models with periodic weightings that have been studied in the literature and for which more results are known. We also discuss the related techniques and explain why they cannot be applied in our case.

The Aztec diamond is a well‐studied tiling model.^5^, ^23^, ^24^, ^25^ It consists of covering the region {(x,y):|x|+|y|≤n+1} with 2 × 1 or 1 × 2 rectangles (called dominos), where n>0 is an integer which parameterizes the size of the covered region. Uniform domino tilings of the Aztec diamond features four solid regions and one liquid region. The associated discrete point process is determinantal, and turns out to belong to the class of Schur processes (introduced in Ref. 26), for which there exists a double contour integral for the kernel that is suitable for an asymptotic analysis as n→+∞. Another important Schur process is the infinite hexagon with 1×k periodic weightings. The infinite hexagon is a nonregular hexagon whose vertical side is first sent to infinity either from above or from below, see, e.g., Ref. 27, fig. 14, for an illustration. For more examples of other interesting tiling models that fall in the Schur process class, see, e.g.,^8^. Uniform lozenge tilings of the finite hexagon (such as $H_{n}$) do not belong to the Schur class, but have been studied using other techniques based on some connections with Hahn polynomials:^1^ the limiting kernel in the bulk scaling regime has been established in Ref. 2 using a discrete RH problem, and in Ref. 3 using the approach developed in Ref. 28.

The doubly periodic Aztec diamond exhibits all three phases. It still defines a determinantal point process, but it falls outside of the Schur process class. However, Chhita and Young found in Ref. 29 a formula for the correlation kernel by performing an explicit inversion of the Kasteleyn matrix. This formula was further simplified in Ref. 30 and then used in Refs. 30, 31 to obtain fine asymptotic results on the fluctuations of the liquid–gas boundary as n→+∞. This same model was analyzed soon afterward in Ref. 20 via a different (and more general) method based on matrix valued OPs and a related RH problem. For the doubly periodic Aztec diamond, this RH problem is surprisingly simple in the sense that it can be solved explicitly for finite *n*. The analysis of Refs. 20, 29, 30 relies on the rather special integrable structure of the doubly periodic Aztec diamond. However, the approach of Ref. 20 applies to a much wider range of tiling models. Berggren and Duits^27^ have recently identified a whole class of tiling/path models for which it is possible to simplify significantly the formula of Ref. 20. Quite remarkably, their final expression for the kernel does not involve any RH problem or OPs, which simplifies substantially the saddle point analysis. Using the results from Ref. 27, Berggren in Ref. 44 recently studied the 2×k periodic Aztec diamond, for an arbitrary *k*. The class of models for which the formula from Ref. 27 applies roughly consists of the models with an infinite number of paths whose (possibly matrix valued) orthogonality weight has a Wiener–Hopf type factorization. This class of models contains the Schur class, but also (among others) the doubly periodic Aztec diamond and doubly periodic lozenge tilings of an infinite hexagon.

However, lozenge tiling models of the finite hexagon cannot be represented as models with infinitely many paths (as opposed to the Aztec diamond and the infinite hexagon). In particular, they do not belong to the class of models studied in Ref. 27 and thus the simplified formula from Ref. 27 cannot be used. This fact makes lozenge tiling models of the finite hexagon harder to analyze asymptotically (see also the comment in Ref. 27, beginning of section 6).

### 1.3 The figures

In addition to being in bijection with nonintersecting paths, lozenge tilings of the hexagon are also in bijection with *dimer coverings*, which are perfect matchings of a certain bipartite graph. We refer to Ref. 4 for more details on the correspondence with dimers (see also Ref. 10, fig. 1, for an illustration). The bijection with dimers is not used explicitly in this paper, but we do use it to generate the pictures via the shuffling algorithm.^32^

## 2 MODEL AND BACKGROUND

In this section, we present a lozenge tiling model with 2 × 2 periodic weightings. We also introduce the necessary material to invoke the double contour formula from Ref. 20 for the kernel. In particular, we present the relevant 2 × 2 matrix valued OPs and the associated 4 × 4 RH problem.

### 2.1 Affine transformation for certain figures of lozenge tilings

For the presentation of the model and the results, it is convenient to define the hexagon and the lozenges as in (2) and (3). However, for the purpose of presenting certain figures of lozenge tilings, it is more pleasant to modify the hexagon and the lozenges by the following simple transformation:
(4)
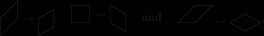



so that $H_{n}$ is mapped by this transformation to a hexagon whose six sides are of equal length. Above the definition (2) of $H_{n}$, we used the standard terminology and called $H_{n}$ “the regular hexagon”; note however that $H_{n}$ becomes truly regular only after applying the transformation (4). In the figures, we will assign the colors red, green, and yellow for the three lozenges in (4), from left to right, respectively.

### 2.2 Definition of the model

**FIGURE 3:**
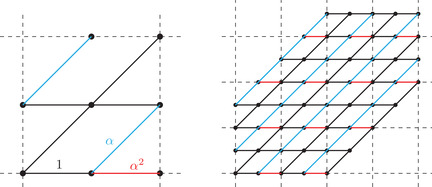
The black edges have weight 1, the cyan edges have weight α, and the red edges have weight α^2^

The regular hexagon $H_{n}$ has corners located at (0,0), (0, *n*), (n,2n), (2n,2n), (2n,n), and (*n*, 0). We normalize the lozenges such that they cover each a surface of area 1, and the vertices of the lozenges have integer coordinates. We recall that each lozenge tiling of $H_{n}$ gives rise, through (3), to a system of *n* nonintersecting paths. These paths live on the graph $G_{n}$, which is illustrated in Figure 2 (left) for n=4. The vertices of $G_{n}$ form a subset of $Z\times (Z+\frac{1}{2})$, and the bottom left vertex has coordinates $\left(0,\frac{1}{2}\right)$. We denote the paths by

(5) $$\begin{array}{c} p_{j}:\left\{0,1,\ldots ,2n\right\}\to \frac{1}{2}+Z,\;j=0,\ldots ,n-1, \end{array}$$

and they satisfy the initial positions $p_{j}\left(0\right)=j+\frac{1}{2}$ and ending positions $p_{j}\left(2n\right)=n+j+\frac{1}{2}$. The particular 2 × 2 periodic lozenge tiling model that we consider depends on a parameter α∈(0,1]. The weightings are defined on the 2 × 2 bottom left block of the lattice as shown in Figure 3 (left), and is then extended by periodicity as shown in Figure 3 (right). More formally, if $e=(\left(x_{1},y_{1}+\frac{1}{2}\right),\left(x_{2},y_{2}+\frac{1}{2}\right))$ is an edge of $G_{n}$, then

(6) $$\begin{array}{c} w_{e}=\left\{\begin{array}{cc} \alpha^{2}, & \text{if}\;x_{1}\;\text{is}\;\text{odd,}\;y_{1}=y_{2},\;\text{and}\;y_{1}\;\text{is}\;\text{even,}\\ \alpha , & \text{if}\;x_{1}+y_{1}\;\text{is}\;\text{odd,}\;\text{and}\;y_{2}=y_{1}+1,\\ 1 & \text{otherwise}. \end{array}\right. \end{array}$$

**FIGURE 4:**
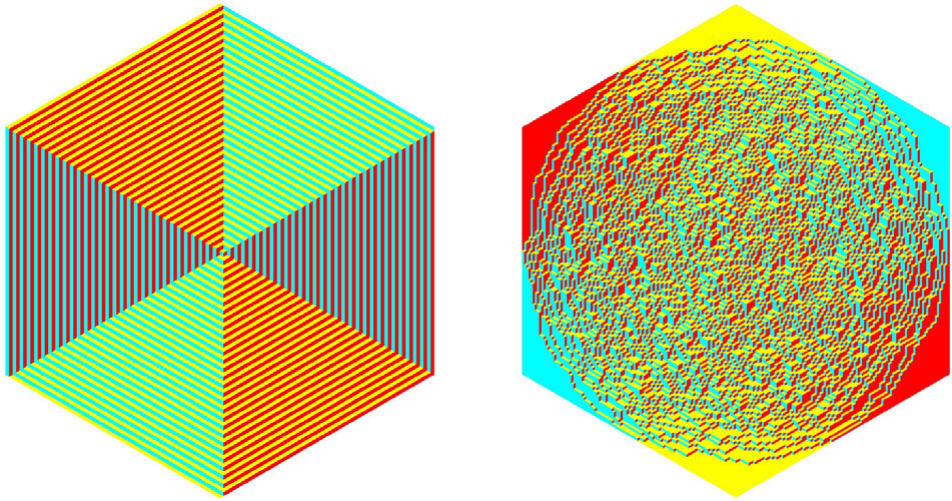
Two tilings taken at random accordingly to the measure induced by (6), for n=60 and $\alpha =5\times 10^{-4}$ (left), and n=100 and α=1 (right)

For any values of α∈(0,1], the weightings (6) are such that W(T)>0 for all T, and thus we have a well‐defined probability measure via (1). On the other hand, if α=0, then several edges have weights 0, and it is easy to see (e.g., from Figure 3 (right)) that W(T)=0 for all T. So in this case, (6) does not induce a probability measure, and this explains why we excluded α=0 in the definition of the model. If α=1, all tilings have the same weight, and we recover the uniform distribution. Proposition 1 states that for α<1, there is a particular tiling $T_{\max}$ that is more likely to appear than any other tiling. $T_{\max}$ is illustrated in Figure 4 (left) for n=60.

Proposition 1 Let α∈(0,1) and let n≥1 be an integer. There exists a unique tiling $T_{\max}$ of $H_{n}$ such that $W\left(T\right)\leq \alpha W\left(T_{\max}\right)$ for all $T\neq T_{\max}$. Furthermore,

$$\begin{array}{c} W\left(T_{\max}\right)=\left\{\begin{array}{cc} \alpha^{\frac{n^{2}}{4}}, & \text{if}\;n\;\text{is}\;\text{even},\\ \alpha^{\frac{n^{2}-1}{4}}, & \text{if}\;n\;\text{is}\;\text{odd.} \end{array}\right. \end{array}$$

The proof of Proposition 1 is based from a careful inspection of $G_{n}$, and we omit the details.▪

**FIGURE 5:**
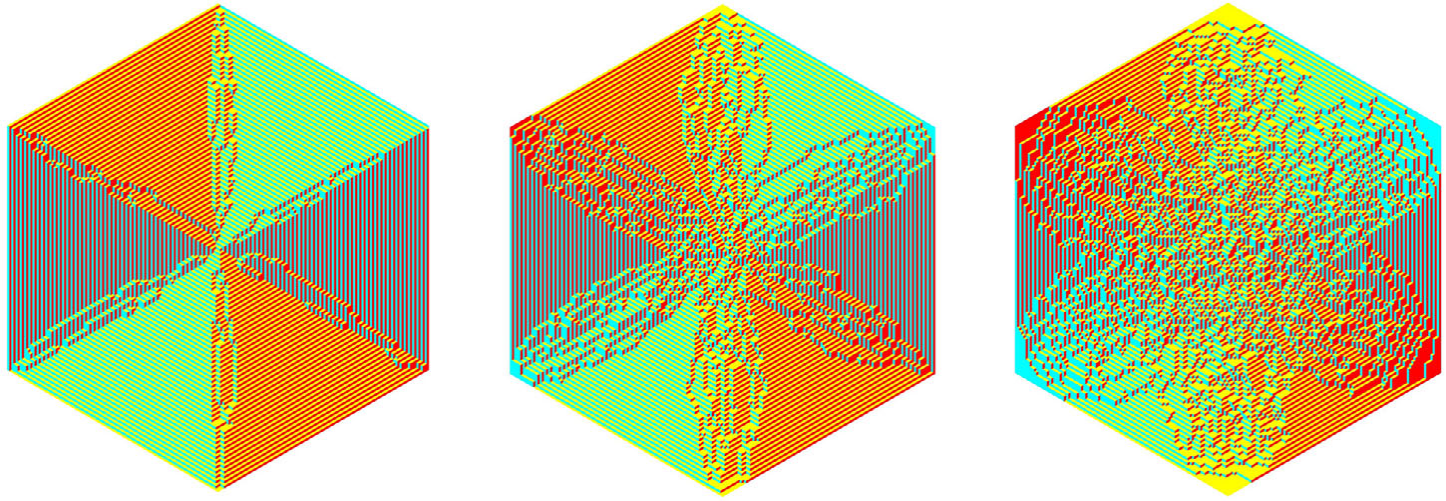
Three tilings taken at random accordingly to the measure induced by (6) with n=100 and α=0.01 (left), α=0.05 (middle), α=0.2 (right)

It follows from Proposition 1 that, as α→0, the randomness disappears because the tiling $T_{\max}$ becomes significantly more likely than any other tiling. Therefore, our model interpolates between the uniform measure over the tilings (for α=1) and a particular totally frozen tiling $T_{\max}$ (as α→0), see Figures 4 and 5. Intriguingly, these figures show similarities with the rectangle–triangle tiling of the hexagon obtained by Keating and Sridhar in Ref. 33, fig. 18.

Several tiling models in the literature (e.g., those considered in Refs. 34 and 21) are defined by weightings on the lozenges, instead of weightings on the edges. To ease possible comparisons with these models, we give an alternative definition of our model. The weight W(T) of a tiling T can alternatively be defined as

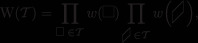



where *w* is the weight function over the lozenges given by
(7)
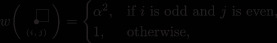

(8)
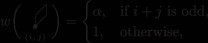



where α∈(0,1]. The above lozenge weightings depend only on the parity of *i* and *j*, and thus are periodic of period 2 in both directions. By using the correspondence (3) between lozenge tilings and nonintersecting paths, it is straightforward to verify that the weightings (7) and (8) define the same measure as the weightings (6).

### 2.3 Matrix valued OPs

It will be convenient for us to define $G_{\infty }$ as the graph whose vertex set is $Z\times (Z+\frac{1}{2})$, and whose edges are of the form $e=(\left(x_{1},y_{1}+\frac{1}{2}\right),\left(x_{2},y_{2}+\frac{1}{2}\right))$ with $x_{2}=x_{1}+1$ and $y_{2}-y_{1}\in \left\{0,1\right\}$. The weighting (6) was defined on the edges of $G_{n}$, but it can be straightforwardly extended to the edges of $G_{\infty }$. We follow the notations of Ref. 20, eq. (57), and denote $T_{x,x+1}\left(y_{1},y_{2}\right)$ for the weight associated to the edge $e=(\left(x,y_{1}+\frac{1}{2}\right),\left(x+1,y_{2}+\frac{1}{2}\right))$ of $G_{\infty }$. This weight can be obtained from (6) and only depends on the parity of *x*. If *x* is even, it is given by

(9) $$\begin{array}{c} T_{x,x+1}\left(y_{1},y_{2}\right)=\left\{\begin{array}{cc} 1 & \text{if}\;y_{2}=y_{1},\\ 1 & \text{if}\;y_{2}=y_{1}+1\;\mathrm{and}\;y_{1}\;\text{is}\;\text{even},\\ \alpha & \text{if}\;y_{2}=y_{1}+1\;\mathrm{and}\;y_{1}\;\text{is}\;\text{odd},\\ 0 & \text{otherwise}, \end{array}\right. \end{array}$$

while if *x* is odd, we have

(10) $$\begin{array}{c} T_{x,x+1}\left(y_{1},y_{2}\right)=\left\{\begin{array}{cc} \alpha^{2} & \text{if}\;y_{2}=y_{1},\;\;\mathrm{and}\;y_{1}\;\text{is}\;\text{even},\\ 1 & \text{if}\;y_{2}=y_{1},\;\;\mathrm{and}\;y_{1}\;\text{is}\;\text{odd},\\ \alpha & \text{if}\;y_{2}=y_{1}+1\;\mathrm{and}\;y_{1}\;\text{is}\;\text{even},\\ 1 & \text{if}\;y_{2}=y_{1}+1\;\mathrm{and}\;y_{1}\;\text{is}\;\text{odd},\\ 0 & \text{otherwise}. \end{array}\right. \end{array}$$

For each x∈Z, $T_{x,x+1}$ is periodic of period 2, namely, $T_{x,x+1}\left(y_{1}+2,y_{2}+2\right)=T_{x,x+1}\left(y_{1},y_{2}\right)$ for all $y_{1},y_{2}\in Z$. The weightings $T_{x,x+1}$ can be represented as two 2 × 2 block Toeplitz matrices (one for *x* even, and one for *x* odd) that are infinite in both directions. These two infinite matrices can be encoded in two 2 × 2 matrix symbols $A_{x,x+1}\left(z\right)$, whose entries ${\left(A_{x,x+1}\left(z\right)\right)}_{i+1,j+1}$, 0≤i,j≤1, are given by

$$\begin{array}{c} {\left(A_{x,x+1}\left(z\right)\right)}_{i+1,j+1}=T_{x,x+1}\left(i,j\right)+zT_{x,x+1}\left(i,j+2\right). \end{array}$$

More explicitly, this gives

(11) $$A_{x,x+1}\left(z\right)=\left\{\begin{array}{cc} \left(\begin{array}{cc} 1 & 1\\ \alpha z & 1 \end{array}\right), & \text{if}\;x\;\text{is}\;\text{even},\\ \left(\begin{array}{cc} \alpha^{2} & \alpha \\ z & 1 \end{array}\right), & \text{if}\;x\;\text{is}\;\text{odd}, \end{array}\right.$$

and we can retrieve the entries of $T_{x,x+1}$ from its symbol by

$$\begin{array}{c} \left(\begin{array}{cc} T_{x,x+1}\left(2y_{1},2y_{2}\right) & \;T_{x,x+1}\left(2y_{1},2y_{2}+1\right)\\ T_{x,x+1}\left(2y_{1}+1,2y_{2}\right) & \;T_{x,x+1}\left(2y_{1}+1,2y_{2}+1\right) \end{array}\right)=\frac{1}{2\pi i}\int_{\gamma }A_{x,x+1}\left(z\right)z^{y_{1}-y_{2}}\frac{dz}{z}, \end{array}$$

where γ is any closed contour going around 0 once in the positive direction. The symbol associated to $G_{n}$ is then obtained by taking the following product (see Ref. 20, eq. (4.9)):

$$\begin{array}{c} A_{0,2n}\left(z\right)=\prod_{x=0}^{2n-1}A_{x,x+1}\left(z\right)=A{\left(z\right)}^{n}, \end{array}$$

where

(12) $$\begin{array}{c} A\left(z\right):=\left(\begin{array}{cc} 1 & \;1\\ \alpha z & \;1 \end{array}\right)\left(\begin{array}{cc} \alpha^{2} & \;\alpha \\ z & \;1 \end{array}\right)=\left(\begin{array}{cc} \alpha^{2}+z & \;1+\alpha \\ (1+\alpha^{3})z & \;1+\alpha^{2}z \end{array}\right). \end{array}$$

To limit the length and technicalities of the paper, from now we take the size of the hexagon even, i.e., n=2N, where *N* is a positive integer. This is made for convenience; the case of odd integer *n* could also be analyzed in a similar way, but then a discussion on the partity of *n* is needed. Because n=2N, following Ref. 20, eq. (4.12), the relevant orthogonality weight to consider is

(13) $$\begin{array}{c} \frac{A{\left(z\right)}^{2N}}{z^{2N}}. \end{array}$$

We consider two families ${\left\{P_{j}\right\}}_{j\geq 0}$ and ${\left\{Q_{j}\right\}}_{j\geq 0}$ of 2 × 2 matrix valued OPs defined by

(14) $$\begin{array}{cccc} & P_{j}\left(z\right)=z^{j}I_{2}+O\left(z^{j-1}\right),\;\text{as}\;z\to \infty \\ & \frac{1}{2\pi i}\int_{\gamma }P_{j}\left(z\right)\frac{A{\left(z\right)}^{2N}}{z^{2N}}z^{k}dz=0_{2}, & & k=0,\ldots ,j-1, \end{array}$$

and

(15) $$\begin{array}{cccc} & \frac{1}{2\pi i}\int_{\gamma }Q_{j}\left(z\right)\frac{A{\left(z\right)}^{2N}}{z^{2N}}z^{j}dz=-I_{2},\\ & \frac{1}{2\pi i}\int_{\gamma }Q_{j}\left(z\right)\frac{A{\left(z\right)}^{2N}}{z^{2N}}z^{k}dz=0_{2}, & & k=0,\ldots ,j-1, \end{array}$$

where 0_2_ denotes the 2 × 2 zero matrix, *I*_2_ is the identity matrix, and γ is, as before, a closed contour surrounding 0 once in the positive direction. Because the weight (13) is not Hermitian, there is no guarantee that the above OPs exist for every *j*. However, it follows from Ref. 20, lemma 4.8 and eq. (4.32), that $P_{N}$ and $Q_{N-1}$ exist.

### 2.4 The 4 × 4 RH problem for *Y*

RH problems for scalar OPs have been introduced by Fokas, Its, and Kitaev in Ref. 35. Here, we need the generalization of this result for matrix valued OPs, which can be found in Refs. 36, 37, 38. Consider the 4 × 4 matrix valued function Y(z)=Y(z;α,N) defined by

(16) $$Y\left(z\right)=\left(\begin{array}{cc} P_{N}\left(z\right) & \frac{1}{2\pi i}\int_{\gamma }P_{N}\left(s\right)\frac{A^{2N}\left(s\right)}{s^{2N}}\frac{ds}{s-z}\\ Q_{N-1}\left(z\right) & \;\frac{1}{2\pi i}\int_{\gamma }Q_{N-1}\left(s\right)\frac{A^{2N}\left(s\right)}{s^{2N}}\frac{ds}{s-z} \end{array}\right),\;z\in C\setminus \gamma .$$

The matrix *Y* is characterized as the unique solution to the following RH problem.

#### RH problem for *Y*

(a) $Y:C\setminus \gamma \to C^{4\times 4}$ is analytic.
(b) The limits of Y(z) as *z* approaches γ from inside and outside exist, are continuous on γ and are denoted by $Y_{+}$ and $Y_{-}$, respectively. Furthermore, they are related by
  (17) $$Y_{+}\left(z\right)=Y_{-}\left(z\right)\left(\begin{array}{cc} I_{2} & \;\frac{A^{2N}\left(z\right)}{z^{2N}}\\ 0_{2} & \;I_{2} \end{array}\right),\;\;\text{for}\;z\in \gamma .$$
(c) As z→∞, we have $Y\left(z\right)=\left(I_{4}+O\left(z^{-1}\right)\right)\left(\begin{array}{cc} z^{N}I_{2} & \;0_{2}\\ 0_{2} & \;z^{-N}I_{2} \end{array}\right)$.

### 2.5 Double contour formula from Ref. 20 for the kernel

As mentioned in the Introduction, the point process obtained by putting points on the paths, as shown in (3), is determinantal. We let *K* denote the associated kernel. By definition of determinantal point processes, for integers k≥1, and $x_{1},\ldots ,x_{k},y_{1},\ldots ,y_{k}$ with $\left(x_{i},y_{i}\right)\neq \left(x_{j},y_{j}\right)$ if i≠j we have

(18) $$\begin{array}{c} P\left[\begin{array}{c} p_{0},\ldots ,p_{2N-1}\;\text{go}\;\text{through}\;\text{each}\;\text{of}\;\text{the}\;\text{points}\\ \left(x_{1},y_{1}+\frac{1}{2}\right),\ldots ,\left(x_{k},y_{k}+\frac{1}{2}\right) \end{array}\right]=\det{\left[K(x_{i},y_{i},x_{j},y_{j})\right]}_{i,j=1}^{k}. \end{array}$$

The following proposition follows after specifying the general result (Ref. 20, theorem 4.7) to our situation.^1^

Proposition 2 *(from Ref. 20)* Let α∈(0,1]. For integers $x_{1},x_{2}\in \left\{1,2,\ldots ,4N-1\right\}$ and $y_{1},y_{2}\in Z$, we have

(19) $$\begin{array}{ccc} {\left[K(x_{1},2y_{1}+j,x_{2},2y_{2}+i)\right]}_{i,j=0}^{1} & & =-\frac{\chi_{x_{1}>x_{2}}}{2\pi i}\int_{\gamma }A_{x_{2},x_{1}}\left(z\right)z^{y_{2}-y_{1}}\frac{dz}{z}\\ & & \;+\frac{1}{{\left(2\pi i\right)}^{2}}\int_{\gamma }\int_{\gamma }\frac{A_{x_{2},4N}\left(w\right)}{w^{2N-y_{2}}}R^{Y}\left(w,z\right)\frac{A_{0,x_{1}}\left(z\right)}{z^{y_{1}+1}}dzdw, \end{array}$$

where $A_{a,b}$ is defined by

$$\begin{array}{c} A_{a,b}\left(z\right)=\prod_{x=a}^{b-1}A_{x,x+1}\left(z\right),\;b>a, \end{array}$$

and $R^{Y}$ is given by

(20) $$\begin{array}{c} R^{Y}\left(w,z\right)=\frac{1}{z-w}\left(\begin{array}{cc} 0_{2} & \;I_{2} \end{array}\right)Y^{-1}\left(w\right)Y\left(z\right)\left(\begin{array}{c} I_{2}\\ 0_{2} \end{array}\right). \end{array}$$

As particular cases of the above, we obtain the following formulas.

Corollary 1 Let α∈(0,1]. For integers x∈{1,…,2N−1} and y∈Z, we have

(21) $$\begin{array}{cc} & {\left[K(2x,2y+j,2x,2y+i)\right]}_{i,j=0}^{1}=\frac{1}{{\left(2\pi i\right)}^{2}}\int_{\gamma }\int_{\gamma }\frac{A{\left(w\right)}^{2N-x}}{w^{2N-y}}R^{Y}\left(w,z\right)\frac{A{\left(z\right)}^{x}}{z^{y+1}}dzdw \end{array}$$

and

(22) $$\begin{array}{cc} & {\left[K(2x+1,2y+j,2x+1,2y+i)\right]}_{i,j=0}^{1}=\\ & \frac{1}{{\left(2\pi i\right)}^{2}}\int_{\gamma }\int_{\gamma }\left(\begin{array}{cc} \alpha^{2} & \;\alpha \\ w & \;1 \end{array}\right)\frac{A{\left(w\right)}^{2N-x-1}}{w^{2N-y}}R^{Y}\left(w,z\right)\frac{A{\left(z\right)}^{x}}{z^{y+1}}\left(\begin{array}{cc} 1 & \;1\\ \alpha z & \;1 \end{array}\right)dzdw. \end{array}$$

This simply follows from

$$\begin{array}{cccc} & A_{0,2x}\left(z\right)=A{\left(z\right)}^{x}, & & A_{2x,4N}\left(w\right)=A{\left(w\right)}^{2N-x},\\ & A_{0,2x+1}\left(z\right)=A{\left(z\right)}^{x}\left(\begin{array}{cc} 1 & \;1\\ \alpha z & \;1 \end{array}\right), & & A_{2x+1,4N}\left(w\right)=\left(\begin{array}{cc} \alpha^{2} & \;\alpha \\ w & \;1 \end{array}\right)A{\left(w\right)}^{2N-x-1}, \end{array}$$

where we have used (11) and (12).▪

From Ref. 20, lemma 4.6, $R^{Y}\left(w,z\right)$ is the unique bivariate polynomial of degree ≤N−1 in both variables *w* and *z*, which satisfies the following reproducing property:

(23) $$\frac{1}{2\pi i}\int_{\gamma }P\left(w\right)\frac{A^{2N}\left(w\right)}{w^{2N}}R^{Y}\left(w,z\right)dw=P\left(z\right),$$

for every 2 × 2 matrix valued polynomial *P* of degree ≤N−1. Because it satisfies (23), $R^{Y}\left(w,z\right)$ is called a reproducing kernel.

## 3 STATEMENT OF RESULTS

The new double contour formula for the kernel in terms of scalar OPs is stated in Theorem 1. In this formula, the large *N* behavior of the integrand is roughly $e^{N\Xi }$, for a certain phase function Ξ, which in our case is defined on a two‐sheeted Riemann surface $R_{\alpha }$. The restriction of Ξ on the first and second sheet are denoted by Φ and Ψ, respectively. The saddle points are the solutions ζ∈C for which either $\Phi^{\prime }\left(\zeta \right)=0$ or $\Psi^{\prime }\left(\zeta \right)=0$. In the liquid region, Proposition 3 states that there is a unique saddle, denoted *s*, lying in the upper half plane. This saddle plays an important role in our analysis, and some of its properties are stated in Propositions 4 and 5. The limiting densities for the lozenges in the liquid region are given explicitly in terms of *s* in Theorem 2.

Remark 1 If α=1, our model reduces to the uniform measure and the kernel can be expressed in terms of scalar‐valued OPs. However, our approach is based on the formulas (21) and (22), and even though these formulas are still valid for α=1, this case requires a special attention (because of a different branch cut structure in the analysis). Because the limiting densities for the lozenges in this case are already well‐known,^39^ from now we will assume that α∈(0,1) to avoid unnecessary discussions.

### 3.1 New formula for the kernel in terms of scalar OPs

We define the scalar weight *W* by

(24) $$W\left(\zeta \right)={\left(\frac{\left(\zeta -\alpha c\right)\left(\zeta -\alpha c^{-1}\right)}{\zeta \left(\zeta -c\right)\left(\zeta -c^{-1}\right)}\right)}^{2N},\;\;\text{where}\;\;c=\sqrt{\frac{\alpha }{1-\alpha +\alpha^{2}}},$$

and consider the following 2 × 2 RH problem.

#### RH problem for *U*

(a) $U:C\setminus \gamma_{C}\to C^{2\times 2}$ is analytic, where $\gamma_{C}$ is a closed curve surrounding *c* and $c^{-1}$ once in the positive direction, but not surrounding 0.
(b) The limits of U(ζ) as ζ approaches $\gamma_{C}$ from inside and outside exist, are continuous on $\gamma_{C}$ and are denoted by $U_{+}$ and $U_{-}$, respectively. Furthermore, they are related by
  (25) $$U_{+}\left(\zeta \right)=U_{-}\left(\zeta \right)\left(\begin{array}{cc} 1 & \;W(\zeta )\\ 0 & \;1 \end{array}\right),\;\;\text{for}\;\zeta \in \gamma_{C}.$$
(c) As ζ→∞, we have $U\left(\zeta \right)=\left(I_{2}+O\left(\zeta^{-1}\right)\right)\left(\begin{array}{cc} \zeta^{2N} & 0\\ 0 & \zeta^{-2N} \end{array}\right)$.

It is known^35^ that the solution *U* to the above RH problem is unique (provided it exists), and can be expressed in terms of scalar‐valued OPs as follows:

$$\begin{array}{c} U\left(\zeta \right)=\left(\begin{array}{cc} p_{2N}\left(\zeta \right) & \;\frac{1}{2\pi i}\int_{\gamma_{C}}\frac{p_{2N}\left(\xi \right)W\left(\xi \right)}{\xi -\zeta }d\xi \\ q_{2N-1}\left(\zeta \right) & \;\frac{1}{2\pi i}\int_{\gamma_{C}}\frac{q_{2N-1}\left(\xi \right)W\left(\xi \right)}{\xi -\zeta }d\xi \end{array}\right),\;\zeta \in C\setminus \gamma_{C}, \end{array}$$

where $p_{2N}$ and $q_{2N-1}$ are polynomials of degree 2*N* and 2N−1, respectively, satisfying the following conditions:

(26) $$\begin{array}{cccc} & p_{2N}\left(\zeta \right)=\zeta^{2N}+O\left(\zeta^{2N-1}\right),\;\text{as}\;\zeta \to \infty ,\\ & \frac{1}{2\pi i}\int_{\gamma_{C}}p_{2N}\left(\zeta \right)W\left(\zeta \right)\zeta^{k}d\zeta =0, & & k=0,\ldots ,2N-1, \end{array}$$

and

(27) $$\begin{array}{cccc} & \frac{1}{2\pi i}\int_{\gamma_{C}}q_{2N-1}\left(\zeta \right)W\left(\zeta \right)\zeta^{2N-1}d\zeta =-1,\\ & \frac{1}{2\pi i}\int_{\gamma_{C}}q_{2N-1}\left(\zeta \right)W\left(\zeta \right)\zeta^{k}d\zeta =0, & & k=0,\ldots ,2N-2. \end{array}$$

The reproducing kernel $R^{U}$ is defined by

(28) $$R^{U}\left(\omega ,\zeta \right)=\frac{1}{\zeta -\omega }\left(\begin{array}{cc} 0 & 1 \end{array}\right)U^{-1}\left(\omega \right)U\left(\zeta \right)\left(\begin{array}{c} 1\\ 0 \end{array}\right).$$

Now, we state our first main result.

Theorem 1 For x∈{1,…,2N−1}, y∈Z, and $\varepsilon_{x}\in \left\{0,1\right\}$, we have

(29) $$\begin{array}{cc} & {\left[K(2x+\varepsilon_{x},2y+j,2x+\varepsilon_{x},2y+i)\right]}_{i,j=0}^{1}=\frac{1}{{\left(2\pi i\right)}^{2}}\int_{\gamma_{C}}\int_{\gamma_{C}}H_{K}\left(\omega ,\zeta ;\varepsilon_{x}\right)\\ & W\left(\omega \right)R^{U}\left(\omega ,\zeta \right)\frac{\omega^{N+x-y}}{\zeta^{N+x-y}}\frac{{\left(\omega -c\right)}^{y}{\left(\omega -c^{-1}\right)}^{y}}{{\left(\zeta -c\right)}^{y}{\left(\zeta -c^{-1}\right)}^{y}}\frac{{\left(\zeta -\alpha c\right)}^{x}{\left(\zeta -\alpha c^{-1}\right)}^{x}}{{\left(\omega -\alpha c\right)}^{x}{\left(\omega -\alpha c^{-1}\right)}^{x}}d\zeta d\omega , \end{array}$$

where $\gamma_{C}$ is a closed curve surrounding *c* and $c^{-1}$ once in the positive direction that does not go around 0, and where $H_{K}\left(\omega ,\zeta ;0\right)$ and $H_{K}\left(\omega ,\zeta ;1\right)$ are given by

(30) $$\begin{array}{cc} & H_{K}\left(\omega ,\zeta ;0\right)=\left(\begin{array}{cc} \frac{1}{\zeta -c} & \;\frac{c(1-\alpha )}{\alpha \left(\zeta -c\right)\left(\zeta -c^{-1}\right)}\\ \frac{\alpha }{\left(1-\alpha \right)c^{2}\omega }\frac{\omega -c}{\zeta -c} & \;\frac{\omega -c}{c\omega \left(\zeta -c\right)\left(\zeta -c^{-1}\right)} \end{array}\right), \end{array}$$

(31) $$\begin{array}{cc} & H_{K}\left(\omega ,\zeta ;1\right)=\left(\begin{array}{cc} \frac{c(\zeta -\alpha c)}{\zeta (\zeta -c)(\omega -\alpha c)} & \;\frac{\left(1-\alpha )c(\zeta -\alpha c\right)}{\left(\zeta -c\right)\left(\zeta -c^{-1}\right)\left(\omega -\alpha c\right)}\\ \frac{\left(\zeta -\alpha c)(\omega -c\right)}{\left(1-\alpha )\zeta (\zeta -c)(\omega -\alpha c\right)} & \;\frac{\left(\zeta -\alpha c)(\omega -c\right)}{\left(\zeta -c\right)\left(\zeta -c^{-1}\right)\left(\omega -\alpha c\right)} \end{array}\right). \end{array}$$

Remark 2 Theorem 1 is proved in Section 6. It is based on an unpublished idea of A. Kuijlaars that matrix valued OPs in a genus zero situation can be reduced to scalar orthogonality. In our case, the scalar orthogonality appears in (26) and (27) and a main part of the proof of Theorem 1 consists of relating the matrix valued reproducing kernel $R^{Y}$ from (20) to the scalar reproducing kernel $R^{U}$ from (28).

### 3.2 The rational function Q

The function Q is a meromorphic function that appears in the equilibrium problem associated to the varying weight *W*. Its explicit expression is obtained after solving a nonlinear system of five equations with five unknowns. Here, we just state the formula for Q, and refer to Section 8 for a more constructive approach. We define Q as follows:

(32) $$\begin{array}{c} Q\left(\zeta \right)=\frac{{\left(\zeta -r_{1}\right)}^{2}{\left(\zeta -r_{2}\right)}^{2}{\left(\zeta -r_{3}\right)}^{2}\left(\zeta -r_{+}\right)\left(\zeta -r_{-}\right)}{4\zeta^{2}{\left(\zeta -\alpha c\right)}^{2}{\left(\zeta -\alpha c^{-1}\right)}^{2}{\left(\zeta -c\right)}^{2}{\left(\zeta -c^{-1}\right)}^{2}}, \end{array}$$

where *c* is given by (24), *r*_1_, *r*_2_, and *r*_3_ are given by

(33) $$\begin{array}{cccccc} & r_{1}=-\sqrt{\alpha }, & & r_{2}=\sqrt{\alpha }\frac{\alpha c+\sqrt{\alpha }}{c+\sqrt{\alpha }}, & & r_{3}=\sqrt{\alpha }\frac{c+\sqrt{\alpha }}{\alpha c+\sqrt{\alpha }}, \end{array}$$

and $r_{+}$ and $r_{-}$ are given by

(34) $$\begin{array}{cccc} & r_{+}=c\;\left(\frac{1+\alpha }{2}+i\sqrt{3}\frac{1-\alpha }{2}\right), & & r_{-}=c\;\left(\frac{1+\alpha }{2}-i\sqrt{3}\frac{1-\alpha }{2}\right). \end{array}$$

The zero $r_{+}$ of Q lies in the upper half plane, $r_{-}=\bar{r_{+}}$, and the other zeros and poles of Q are real. Furthermore, for all α∈(0,1), they are ordered as follows:

(35) $$\begin{array}{c} r_{1}<0<\alpha c<r_{2}<\alpha c^{-1}<c<r_{3}<c^{-1}. \end{array}$$

### 3.3 Lozenge probabilities

The densities for the three types of lozenges at a point (x,y), x,y∈{0,1,…,4N}, are denoted by
(36)
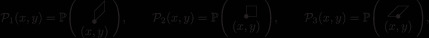



and satisfy $\sum_{j=1}^{3}P_{j}\left(x,y\right)=1$. Because our model is 2 × 2 periodic, $P_{1}\left(x,y\right)$, $P_{2}\left(x,y\right)$, and $P_{3}\left(x,y\right)$ depend crucially on the parity of *x* and *y*, and it is convenient to consider the following matrices:

(37) $$\begin{array}{c} P_{j}\left(x,y\right)=\left(\begin{array}{cc} P_{j}\left(2x,2y+1\right) & \;P_{j}\left(2x+1,2y+1\right)\\ P_{j}\left(2x,2y\right) & P_{j}\left(2x+1,2y\right) \end{array}\right),\;j=1,2,3, \end{array}$$

where x,y∈{0,1,…,2N−1}. Let ${\left\{\left(x_{N},y_{N}\right)\right\}}_{N\geq 1}$ be a sequence satisfying

(38) $$\begin{array}{cc} & \left\{\begin{array}{c} \frac{x_{N}}{N}=1+\xi +o\left(1\right),\\ \frac{y_{N}}{N}=1+\eta +o\left(1\right), \end{array}\right.\;\text{as}\;N\to +\infty , \end{array}$$

where the point (ξ,η) lies in the hexagon

(39) $$H=\left\{(\xi ,\eta )|-1\leq \xi \leq 1,\;-1\leq \eta \leq 1,\;-1\leq \eta -\xi \leq 1\right\}.$$

In Theorem 2, we give explicit expressions for

(40) $$\begin{array}{c} \lim_{N\to +\infty }P_{j}\left(x_{N},y_{N}\right),\;j=1,2,3, \end{array}$$

in case (ξ,η) belongs to the liquid region $L_{\alpha }\subset H$.

### 3.4 Saddle points and the liquid region

For each (ξ,η)∈H, there are in total eight saddles for the double contour integral (29), which are the solutions to the algebraic equation

(41) $$\begin{array}{c} {\left[\frac{\xi -\eta }{2}\frac{1}{\zeta }-\frac{\xi }{2}\left(\frac{1}{\zeta -\alpha c}+\frac{1}{\zeta -\alpha c^{-1}}\right)+\frac{\eta }{2}\left(\frac{1}{\zeta -c}+\frac{1}{\zeta -c^{-1}}\right)\right]}^{2}=Q\left(\zeta \right), \end{array}$$

where Q(ζ) is given by (32). Following the previous works,^10^, ^21^, ^40^, ^41^, ^42^ we define the liquid region as the subset of H for which there exists a saddle lying in the upper half‐plane $C^{+}=\left\{\zeta \in C:\text{Im}\;\zeta >0\right\}$. Proposition 3 states that there is a unique such saddle (whenever it exists), which is denoted by s(ξ,η;α). This saddle plays a particular role in the analysis of Section 11 and appears in the final formulas for the limiting densities (40).

Proposition 3 Let $\left(\xi ,\eta \right)\in H^{o}$ (the interior set of H). Then, there exists at most one solution ζ=s(ξ,η;α) to (41) in $C^{+}=\left\{\zeta \in C|\text{Im}\;\zeta >0\right\}$.

Definition 1 We define the liquid region $L_{\alpha }\subset H$ by

(42) $$L_{\alpha }=\left\{\left(\xi ,\eta \right)\in H^{o}|\text{there}\;\text{exists}\;a\;\text{solution}\;\zeta =s\left(\xi ,\eta ;\alpha \right)\in C^{+}\;\text{to}\;\left(41\right)\right\}$$

and we define the map $s:L_{\alpha }\to C^{+}$ by (ξ,η)↦s(ξ,η;α).

It is clear from (32) and (41) that $\left(0,0\right)\in L_{\alpha }$ and $s\left(0,0;\alpha \right)=r_{+}$ for all α∈(0,1). We now describe some properties of (ξ,η)↦s(ξ,η;α). Consider the following three circles:

$$\begin{array}{cc} & \gamma_{0}=\left\{\zeta \in C:|\zeta \right|=R_{0}\},\;\gamma_{\alpha }=\{\zeta \in C:|\zeta -\alpha c^{-1}|=R_{\alpha }\},\;\gamma_{1}=\{\zeta \in C:|\zeta -c^{-1}\left|=R_{1}\right\}, \end{array}$$

where $R_{0}=\sqrt{\alpha }$, $R_{\alpha }=\left(1-\alpha \right)\sqrt{\alpha }$, and $R_{1}=\frac{1-\alpha }{\sqrt{\alpha }}$ (see also Figure 10). It is a direct computation to verify that

(43) $$\begin{array}{c} r_{+},r_{-},r_{2}\in \gamma_{1},\;r_{+},r_{-},r_{3}\in \gamma_{\alpha },\;\;\text{and}\;\;r_{+},r_{-},r_{1}\in \gamma_{0}. \end{array}$$

In particular, we can write

$$\begin{array}{c} r_{\pm }=c^{-1}+R_{1}e^{\pm i\theta_{1}}=\alpha c^{-1}+R_{\alpha }e^{\pm i\theta_{\alpha }}=R_{0}e^{\pm i\theta_{0}}, \end{array}$$

for certain angles $\theta_{1}\in \left(\frac{2\pi }{3},\pi \right)$, $\theta_{\alpha }\in \left(\frac{\pi }{3},\frac{2\pi }{3}\right)$, and $\theta_{0}\in \left(0,\frac{\pi }{3}\right)$. We also define

(44) $$\begin{array}{cc} & \Sigma_{1}=\{\zeta \in C:|\zeta -c^{-1}\left|=R_{1},\;\arg z\in \left(-\theta_{1},\theta_{1}\right)\right\}\subset \gamma_{1}, \end{array}$$

(45) $$\begin{array}{cc} & \Sigma_{\alpha }=\{\zeta \in C:|\zeta -\alpha c^{-1}\left|=R_{\alpha },\;\arg z\in \left(-\pi ,-\theta_{\alpha }\right)\cup \left(\theta_{\alpha },\pi \right]\right\}\subset \gamma_{\alpha }, \end{array}$$

(46) $$\begin{array}{cc} & \Sigma_{0}=\left\{\zeta \in C:|\zeta \right|=R_{0},\;\arg z\in \left(-\theta_{0},\theta_{0}\right)\}\subset \gamma_{0}. \end{array}$$

**FIGURE 6:**
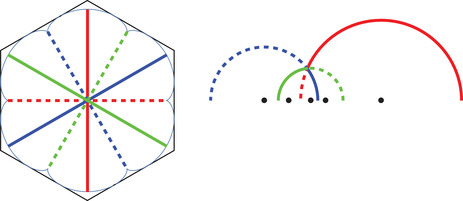
On the left, we draw for α=0.4 the parts of the lines ξ=0 (red), $\eta =\frac{\xi }{2}$ (dashed red), η=ξ (blue), η=−ξ (dashed blue), η=0 (green) and η=2ξ (dashed green) that are in the liquid region. On the right, we draw the corresponding location of s(ξ,η;α) in the upper half plane. The black dots are, from left to right, 0, αc, $\alpha c^{-1}$, *c* and $c^{-1}$

The following proposition is illustrated in Figure 6.

Remark 3 For a given set *A*, the notation $\bar{A}$ stands for the closure of *A*.

Proposition 4 The map (ξ,η)↦s(ξ,η;α) satisfies s(−ξ,−η;α)=s(ξ,η;α), and

(a) it maps $\left\{\xi =0\right\}\cap L_{\alpha }$ onto $\bar{\Sigma_{1}}\cap C^{+}$,
(b) it maps $\left\{\eta =\frac{\xi }{2}\right\}\cap L_{\alpha }$ onto $\left(\gamma_{1}\setminus \Sigma_{1}\right)\cap C^{+}$,
(c) it maps $\left\{\eta =\xi \right\}\cap L_{\alpha }$ onto $\bar{\Sigma_{0}}\cap C^{+}$,
(d) it maps $\left\{\eta =-\xi \right\}\cap L_{\alpha }$ onto $\left(\gamma_{0}\setminus \Sigma_{0}\right)\cap C^{+}$,
(e) it maps $\left\{\eta =0\right\}\cap L_{\alpha }$ onto $\bar{\Sigma_{\alpha }}\cap C^{+}$,
(f) it maps $\left\{\eta =2\xi \right\}\cap L_{\alpha }$ onto $\left(\gamma_{\alpha }\setminus \Sigma_{\alpha }\right)\cap C^{+}$.

By definition, the saddles lie in the complex plane. We show here that they can be naturally projected on a Riemann surface. Define $Q{\left(\zeta \right)}^{1/2}$ with a branch cut joining $r_{-}$ to $r_{+}$ along Σ_1_, such that $Q{\left(\zeta \right)}^{1/2}∼\frac{1}{2\zeta }$ as ζ→∞, and denote the associated Riemann surface by $R_{\alpha }$:

$$\begin{array}{c} R_{\alpha }:=\left\{\left(\zeta ,w\right)\in C^{2}:w^{2}=Q\left(\zeta \right)\right\}. \end{array}$$

This is a two‐sheeted covering of the ζ‐plane glued along Σ_1_, and the sheets are ordered such that $w=Q{\left(\zeta \right)}^{1/2}$ on the first sheet and $w=-Q{\left(\zeta \right)}^{1/2}$ on the second sheet. For each solution ζ to (41), there exists a *w* satisfying $w^{2}=Q\left(\zeta \right)$, and such that

(47) $$\begin{array}{c} \frac{\xi -\eta }{2}\frac{1}{\zeta }-\frac{\xi }{2}\left(\frac{1}{\zeta -\alpha c}+\frac{1}{\zeta -\alpha c^{-1}}\right)+\frac{\eta }{2}\left(\frac{1}{\zeta -c}+\frac{1}{\zeta -c^{-1}}\right)=w. \end{array}$$

Definition 2 The map (ξ,η)↦w(ξ,η;α) is defined by $w{\left(\xi ,\eta ;\alpha \right)}^{2}=Q\left(s\left(\xi ,\eta ;\alpha \right)\right)$, such that (47) holds with ζ=s(ξ,η;α) and w=w(ξ,η;α).

Proposition 5 The map (ξ,η)↦(s(ξ,η;α),w(ξ,η;α)) is a diffeomorphism from $L_{\alpha }$ to

(48) $$\begin{array}{c} R_{\alpha }^{+}:=\left\{\left(\zeta ,w\right)\in R_{\alpha }|\text{Im}\;\zeta >0\right\}. \end{array}$$

It maps the left half $L_{\alpha }^{l}=\left\{\left(\xi ,\eta \right)\in L_{\alpha }|\xi <0\right\}$ to the upper half‐plane of the first sheet of $R_{\alpha }$, and it maps $L_{\alpha }^{r}=\left\{\left(\xi ,\eta \right)\in L_{\alpha }|\xi >0\right\}$ to the upper half‐plane of the second sheet. Moreover, its inverse (s,w)↦(ξ,η)=(ξ(s,w;α),η(s,w;α)) is explicitly given by

(49) $$\begin{array}{c} \left(\begin{array}{c} \xi \\ \eta \end{array}\right)={\left(\begin{array}{cc} \text{Re}\;\left(\frac{-\left(s-\alpha \right)\left(s+\alpha \right)\left(s-c\right)\left(s-\frac{1}{c}\right)}{\left(s-\alpha c\right)\left(s-\frac{\alpha }{c}\right)\left(s-1\right)\left(s+1\right)}\right) & 1\\ \text{Im}\;\left(\frac{-\left(s-\alpha \right)\left(s+\alpha \right)\left(s-c\right)\left(s-\frac{1}{c}\right)}{\left(s-\alpha c\right)\left(s-\frac{\alpha }{c}\right)\left(s-1\right)\left(s+1\right)}\right) & 0 \end{array}\right)}^{-1}\left(\begin{array}{c} \text{Re}\;\left(\frac{2s\left(s-c\right)\left(s-\frac{1}{c}\right)}{\left(s-1)(s+1\right)}w\right)\\ \text{Im}\;\left(\frac{2s\left(s-c\right)\left(s-\frac{1}{c}\right)}{\left(s-1)(s+1\right)}w\right) \end{array}\right). \end{array}$$

##### Description of the liquid region

After clearing the denominator in (41), we get

(50) $$\begin{array}{c} {\left(\zeta -r_{1}\right)}^{2}{\left(\zeta -r_{2}\right)}^{2}{\left(\zeta -r_{3}\right)}^{2}\left(\zeta -r_{+}\right)\left(\zeta -r_{-}\right)=\\ {\left[\left(\zeta -1\right)\left(\zeta +1\right)\left(\zeta -\alpha c\right)\left(\zeta -\frac{\alpha }{c}\right)\eta -\left(\zeta -\alpha \right)\left(\zeta +\alpha \right)\left(\zeta -c\right)\left(\zeta -\frac{1}{c}\right)\xi \right]}^{2}. \end{array}$$

**FIGURE 7:**
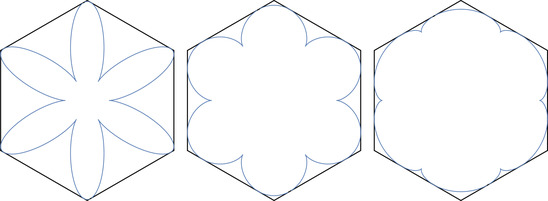
The curve $\partial L_{\alpha }$ with α=0.04, α=0.2, and α=0.4 (from left to right)

Because (50) is invariant under the map (ξ,η)↦(−ξ,−η), we conclude that $L_{\alpha }$ is symmetric with respect to the origin. Also, this equation has real coefficients, so s(ξ,η;α) and $\bar{s(\xi ,\eta ;\alpha )}$ are both solutions whenever $\left(\xi ,\eta \right)\in L_{\alpha }$. At the boundary $\partial L_{\alpha }$ of the liquid region, s(ξ,η;α) and $\bar{s(\xi ,\eta ;\alpha )}$ coalesce in the real line, so $\partial L_{\alpha }$ is part of the zero set of the discriminant of (50) (whose expression is too long to be written down). The curve $\partial L_{\alpha }$ is tangent to the hexagon at 12 points and possesses six cusp points. The tangent points can be obtained by letting $s\to s_{*}\in \left\{0,\alpha c,\alpha c^{-1},c,c^{-1},\infty \right\}$ in (49), and the cusp points by letting $s\to s_{*}\in \left\{r_{1},r_{2},r_{3}\right\}$ in (49) (see also Figure 6). Figure 7 illustrates $\partial L_{\alpha }$ for different values of α (and has been generated using (49)). Denote $F_{\alpha ,j}$, j=1,…,6 for the regions shown in Figure 9 (left). They are disjoint from each other and from $L_{\alpha }$, and are symmetric under (ξ,η)↦(−ξ,−η). As we will see, these regions are frozen (or semifrozen). From Propositions 4 and 5, we already infer the following:

(51a) $$\begin{array}{cccc} & s,\bar{s}\to s_{*}\in \left(0,\alpha c\right), & & \text{as}\;\left(\xi ,\eta \right)\to \left(\xi^{*},\eta^{*}\right)\in \partial L_{\alpha }\cap \partial F_{1,\alpha }, \end{array}$$

(51b) $$\begin{array}{cccc} & s,\bar{s}\to s_{*}\in \left(\alpha c^{-1},c\right), & & \text{as}\;\left(\xi ,\eta \right)\to \left(\xi^{*},\eta^{*}\right)\in \partial L_{\alpha }\cap \partial F_{2,\alpha }, \end{array}$$

(51c) $$\begin{array}{cccc} & s,\bar{s}\to s_{*}\in \left(c^{-1},+\infty \right), & & \text{as}\;\left(\xi ,\eta \right)\to \left(\xi^{*},\eta^{*}\right)\in \partial L_{\alpha }\cap \partial F_{3,\alpha }, \end{array}$$

(51d) $$\begin{array}{cccc} & s,\bar{s}\to s_{*}\in \left(\alpha c,\alpha c^{-1}\right), & & \text{as}\;\left(\xi ,\eta \right)\to \left(\xi^{*},\eta^{*}\right)\in \partial L_{\alpha }\cap \partial F_{4,\alpha }, \end{array}$$

(51e) $$\begin{array}{cccc} & s,\bar{s}\to s_{*}\in \left(-\infty ,0\right), & & \text{as}\;\left(\xi ,\eta \right)\to \left(\xi^{*},\eta^{*}\right)\in \partial L_{\alpha }\cap \partial F_{5,\alpha }, \end{array}$$

(51f) $$\begin{array}{cccc} & s,\bar{s}\to s_{*}\in \left(c,c^{-1}\right), & & \text{as}\;\left(\xi ,\eta \right)\to \left(\xi^{*},\eta^{*}\right)\in \partial L_{\alpha }\cap \partial F_{6,\alpha }. \end{array}$$

### 3.5 Limiting densities in the liquid region

**FIGURE 8:**
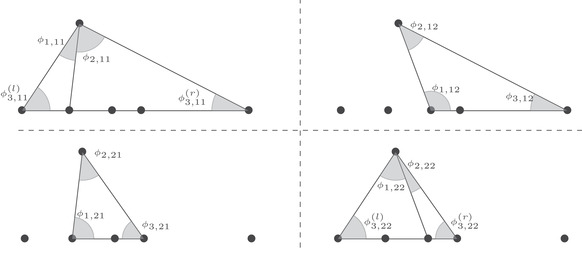
In each of the four quadrants, the five collinear dots represent, from left to right, the points 0, αc, $\alpha c^{-1}$, *c*, and $c^{-1}$. The other dot represents s(ξ,η;α). The figures are made for α=0.4, ξ=−0.325, and η=0.256

Theorem 2 states that the limits (40) are expressed in terms of the angles shown in Figure 8.

Theorem 2 Let $\{{\left(x_{N},y_{N}\right\}}_{N\geq 1}$ be a sequence satisfying (38) with $\left(\xi ,\eta \right)\in L_{\alpha }$. We obtain the following limits:

(52) $$\begin{array}{cc} & \lim_{N\to \infty }P_{1}\left(x_{N},y_{N}\right)=\frac{1}{\pi }\left(\begin{array}{cc} \arg(s-\alpha c)-\arg(s) & \arg(s-\alpha c^{-1})\\ \arg(s-\alpha c) & \arg(s-\alpha c^{-1})-\arg s \end{array}\right), \end{array}$$

(53) $$\begin{array}{cc} & \lim_{N\to \infty }P_{2}\left(x_{N},y_{N}\right)=\frac{1}{\pi }\left(\begin{array}{cc} \arg\left(s-c^{-1}\right)-\arg\left(s-\alpha c\right) & \;\arg\left(s-c^{-1}\right)-\arg\left(s-\alpha c^{-1}\right)\\ \arg(s-c)-\arg(s-\alpha c) & \;\arg\left(s-c\right)-\arg\left(s-\alpha c^{-1}\right) \end{array}\right), \end{array}$$

(54) $$\begin{array}{cc} & \lim_{N\to \infty }P_{3}\left(x_{N},y_{N}\right)=\frac{1}{\pi }\left(\begin{array}{cc} \pi -\arg\left(s-c^{-1}\right)+\arg\left(s\right) & \pi -\arg(s-c^{-1})\\ \pi -\arg(s-c) & \pi -\arg(s-c)+\arg(s) \end{array}\right). \end{array}$$

These limits can equivalently be stated as follows:

$$\begin{array}{cc} & \lim_{N\to \infty }P_{1}\left(x_{N},y_{N}\right)=\frac{1}{\pi }\left(\begin{array}{cc} \phi_{1,11} & \;\phi_{1,12}\\ \phi_{1,21} & \;\phi_{1,22} \end{array}\right),\\ & \lim_{N\to \infty }P_{2}\left(x_{N},y_{N}\right)=\frac{1}{\pi }\left(\begin{array}{cc} \phi_{2,11} & \;\phi_{2,12}\\ \phi_{2,21} & \;\phi_{2,22} \end{array}\right),\\ & \lim_{N\to \infty }P_{3}\left(x_{N},y_{N}\right)=\frac{1}{\pi }\left(\begin{array}{cc} \phi_{3,11}^{\left(l\right)}+\phi_{3,11}^{\left(r\right)} & \;\phi_{3,12}\\ \phi_{3,21} & \;\phi_{3,22}^{\left(l\right)}+\phi_{3,22}^{\left(r\right)} \end{array}\right), \end{array}$$

where $\phi_{k,ij}$, 1≤i,j,k≤2, and $\phi_{3,11}^{\left(l\right)}$, $\phi_{3,11}^{\left(r\right)}$, ϕ_3, 12_, ϕ_3, 21_, $\phi_{3,22}^{\left(l\right)}$, and $\phi_{3,22}^{\left(r\right)}$ are the angles represented in Figure 8.

By combining (51) with Theorem 2, we obtain the following immediate corollary:

Corollary 2 Let $\{{\left(x_{N},y_{N}\right\}}_{N\geq 1}$ be a sequence satisfying (38) with $\left(\xi ,\eta \right)\in L_{\alpha }$. We have

$$\begin{array}{cccc} & \lim_{N\to \infty }P_{j}\left(x_{N},y_{N}\right)\to \left\{\left(\begin{array}{cc} 1 & \;1\\ 1 & \;1 \end{array}\right),\left(\begin{array}{cc} 0 & \;0\\ 0 & \;0 \end{array}\right),\left(\begin{array}{cc} 0 & \;0\\ 0 & \;0 \end{array}\right)\right\} & & \text{as}\;\left(\xi ,\eta \right)\to \left(\xi^{*},\eta^{*}\right)\in \partial L_{\alpha }\cap \partial F_{1,\alpha },\\ & \lim_{N\to \infty }P_{j}\left(x_{N},y_{N}\right)\to \left\{\left(\begin{array}{cc} 0 & \;0\\ 0 & \;0 \end{array}\right),\left(\begin{array}{cc} 1 & \;1\\ 1 & \;1 \end{array}\right),\left(\begin{array}{cc} 0 & \;0\\ 0 & \;0 \end{array}\right)\right\} & & \text{as}\;\left(\xi ,\eta \right)\to \left(\xi^{*},\eta^{*}\right)\in \partial L_{\alpha }\cap \partial F_{2,\alpha },\\ & \lim_{N\to \infty }P_{j}\left(x_{N},y_{N}\right)\to \left\{\left(\begin{array}{cc} 0 & \;0\\ 0 & \;0 \end{array}\right),\left(\begin{array}{cc} 0 & \;0\\ 0 & \;0 \end{array}\right),\left(\begin{array}{cc} 1 & \;1\\ 1 & \;1 \end{array}\right)\right\} & & \text{as}\;\left(\xi ,\eta \right)\to \left(\xi^{*},\eta^{*}\right)\in \partial L_{\alpha }\cap \partial F_{3,\alpha },\\ & \lim_{N\to \infty }P_{j}\left(x_{N},y_{N}\right)\to \left\{\left(\begin{array}{cc} 0 & \;1\\ 0 & \;1 \end{array}\right),\left(\begin{array}{cc} 1 & \;0\\ 1 & \;0 \end{array}\right),\left(\begin{array}{cc} 0 & \;0\\ 0 & \;0 \end{array}\right)\right\} & & \text{as}\;\left(\xi ,\eta \right)\to \left(\xi^{*},\eta^{*}\right)\in \partial L_{\alpha }\cap \partial F_{4,\alpha },\\ & \lim_{N\to \infty }P_{j}\left(x_{N},y_{N}\right)\to \left\{\left(\begin{array}{cc} 0 & \;1\\ 1 & \;0 \end{array}\right),\left(\begin{array}{cc} 0 & \;0\\ 0 & \;0 \end{array}\right),\left(\begin{array}{cc} 1 & \;0\\ 0 & \;1 \end{array}\right)\right\} & & \text{as}\;\left(\xi ,\eta \right)\to \left(\xi^{*},\eta^{*}\right)\in \partial L_{\alpha }\cap \partial F_{5,\alpha },\\ & \lim_{N\to \infty }P_{j}\left(x_{N},y_{N}\right)\to \left\{\left(\begin{array}{cc} 0 & \;0\\ 0 & \;0 \end{array}\right),\left(\begin{array}{cc} 1 & \;1\\ 0 & \;0 \end{array}\right),\left(\begin{array}{cc} 0 & \;0\\ 1 & \;1 \end{array}\right)\right\} & & \text{as}\;\left(\xi ,\eta \right)\to \left(\xi^{*},\eta^{*}\right)\in \partial L_{\alpha }\cap \partial F_{6,\alpha }, \end{array}$$

where the three matrices inside each brackets correspond, from left to right, to j=1,2,3.

**FIGURE 9:**
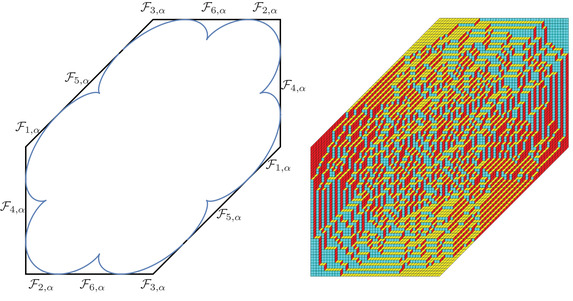
The six frozen regions for α=0.3, and a tiling of size n=48

From Figure 9 (right), it transpires that the regions $F_{j,\alpha }$, j=1,2,3 are frozen, and that $F_{j,\alpha }$, j=4,5,6 are semifrozen. More precisely, let (x,y)∈{0,…,2N−1} be such that $\left(\xi ,\eta \right)=\left(\frac{x}{N}-1,\frac{y}{N}-1\right)\in F_{j,\alpha }$, j∈{1,…,6}. In Figure 9 (right), we observe that

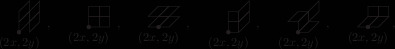



depending on whether $\left(\xi ,\eta \right)\in F_{j,\alpha }$, j=1,…,6, respectively. Corollary 2 describes the situation at the boundary of the liquid region, and is consistent with these observations.

### 3.6 Outline of the rest of the paper

The proofs of Propositions 3, 4, and 5 are rather direct and are presented in Section 4. In Section 5, we follow an idea of Ref. 20 and perform an eigendecomposition of the matrix valued weight. The eigenvalues and eigenvectors are naturally related to a two‐sheeted Riemann surface M. The proof of Theorem 1 is given in Section 6, and relies on the fact that M is of genus 0. The proof of Theorem 2 is done via a saddle point analysis in Section 11, after considerable preparations have been carried out in Sections 7, 10:

- In Section 7, we use Theorem 1 to find double contour formulas for the lozenges in terms of scalar OPs. We also use the symmetry in our model to conclude that it is sufficient to prove Theorem 2 for the lower left quadrant of the liquid region.
- In Section 9, we will perform a Deift/Zhou^22^ steepest descent analysis on the RH problem for *U*. This analysis goes via a series of transformations U↦T↦S↦R. The first transformation U↦T uses a so‐called *g*‐function, which is obtained in Section 8.
- The functions Φ and Ψ denote the restrictions of the phase function Ξ to the first and second sheets of $R_{\alpha }$ and play a central role in the large *N* analysis of the kernel. In Section 10, we study the level set $N_{\Phi }=\left\{\zeta \in C:\text{Re}\;\Phi \left(\zeta \right)=\text{Re}\;\Phi \left(s\right)\right\}$, which is of crucial importance to find the contour deformations that we need to consider for the saddle point analysis.

As mentioned in Remark 1, we will always assume that α∈(0,1), even though it will not be written explicitly.

## 4 PROOFS OF PROPOSITIONS 3, 4(a), and 5

### 4.1 Proof of Proposition 3

By (50), the saddles are the zeros of the polynomial *M* given by

$$\begin{array}{c} M\left(\zeta \right)={\left(\zeta -r_{1}\right)}^{2}{\left(\zeta -r_{2}\right)}^{2}{\left(\zeta -r_{3}\right)}^{2}\left(\zeta -r_{+}\right)\left(\zeta -r_{-}\right)-\\ {\left[\left(\zeta -1\right)\left(\zeta +1\right)\left(\zeta -\alpha c\right)\left(\zeta -\frac{\alpha }{c}\right)\eta -\left(\zeta -\alpha \right)\left(\zeta +\alpha \right)\left(\zeta -c\right)\left(\zeta -\frac{1}{c}\right)\xi \right]}^{2}. \end{array}$$

Because the coefficients of *M* are real, Proposition 3 follows if *M* has at least six zeros on the real line. This can be proved by a direct inspection of the values of M(ζ) at $\zeta =-\infty ,r_{1},0,\alpha c,r_{2},\frac{\alpha }{c},c,r_{3},c^{-1},+\infty$:

$$\begin{array}{cc} & M\left(r_{1}\right)=-\frac{\alpha }{c^{2}}{\left(1-\alpha \right)}^{2}{\left(c+\sqrt{\alpha }\right)}^{2}{\left(\alpha c+\sqrt{\alpha }\right)}^{2}{\left(\eta +\xi \right)}^{2},\;M\left(0\right)=\alpha^{4}\left(1-{\left(\eta -\xi \right)}^{2}\right),\\ & M\left(\alpha c\right)={\left(1-\alpha \right)}^{8}c^{8}\left(1-\xi^{2}\right),\;M\left(r_{2}\right)=-\frac{\alpha {\left(1-\alpha \right)}^{10}c^{8}}{{\left(c+\sqrt{\alpha }\right)}^{8}}{\left(\left(1+\alpha^{2}\right)c+\sqrt{\alpha }\left(1+\alpha \right)\right)}^{2}{\left(\xi -2\eta \right)}^{2},\\ & M\left(\alpha c^{-1}\right)=\alpha^{4}{\left(1-\alpha \right)}^{8}\left(1-\xi^{2}\right),\;M\left(c\right)={\left(1-\alpha \right)}^{8}c^{8}\left(1-\eta^{2}\right),\\ & M\left(r_{3}\right)=-\frac{\alpha {\left(1-\alpha \right)}^{10}c^{8}}{{\left(\alpha c+\sqrt{\alpha }\right)}^{8}}{\left(\left(1+\alpha^{2}\right)c+\sqrt{\alpha }\left(1+\alpha \right)\right)}^{2}{\left(\eta -2\xi \right)}^{2},\;M\left(c^{-1}\right)=\frac{{\left(1-\alpha \right)}^{8}}{\alpha^{4}}\left(1-\eta^{2}\right). \end{array}$$

Because $\left(\xi ,\eta \right)\in H^{o}$, where

$$\begin{array}{c} H^{o}=\left\{(\xi ,\eta )|-1<\xi <1,\;-1<\eta <1,\;-1<\eta -\xi <1\right\}, \end{array}$$

the leading coefficients of *M* are $1-{\left(\xi -\eta \right)}^{2}>0$. We conclude the following:

1. if η≠−ξ, *M* has at least one simple root on $\left(-\infty ,r_{1}\right)$ and at least one simple root on (*r*_1_, 0),
2. if $\eta \neq \frac{\xi }{2}$, *M* has at least one simple root on $\left(\alpha c,r_{2}\right)$ and at least one simple root on $\left(r_{2},\frac{\alpha }{c}\right)$,
3. if η≠2ξ, *M* has at least one simple root on $\left(c,r_{3}\right)$ and at least one simple root on $\left(r_{3},\frac{1}{c}\right)$.

Finally, other computations show that $M^{\prime }\left(r_{1}\right)=0$ if η=−ξ, that $M^{\prime }\left(r_{2}\right)=0$ if $\eta =\frac{\xi }{2}$ and that $M^{\prime }\left(r_{3}\right)=0$ if η=2ξ. So *M* has at least 6 real zeros (counting multiplicities) for each $\left(\xi ,\eta \right)\in H^{o}$.

### 4.2 Proof of Propositions 4 and 5

We start with the proof of Proposition 5. By rearranging the terms in (47), we see that the saddles are the solutions to

$$\begin{array}{c} \left[\frac{1}{2\zeta }-\frac{1}{2}\left(\frac{1}{\zeta -\alpha c}+\frac{1}{\zeta -\alpha c^{-1}}\right)\right]\xi +\left[-\frac{1}{2\zeta }+\frac{1}{2}\left(\frac{1}{\zeta -c}+\frac{1}{\zeta -c^{-1}}\right)\right]\eta =w, \end{array}$$

where *w* satisfies $w^{2}=Q\left(\zeta \right)$. This can be rewritten as

(55) $$\begin{array}{c} \frac{-\left(\zeta -\alpha \right)\left(\zeta +\alpha \right)\left(\zeta -c\right)\left(\zeta -\frac{1}{c}\right)}{\left(\zeta -\alpha c\right)\left(\zeta -\frac{\alpha }{c}\right)\left(\zeta -1\right)\left(\zeta +1\right)}\xi +\eta =\frac{2\zeta \left(\zeta -c\right)\left(\zeta -\frac{1}{c}\right)}{\left(\zeta -1)(\zeta +1\right)}w. \end{array}$$

Taking the real and imaginary parts of (55), and recalling that ξ,η∈R, we get

(56) $$\begin{array}{c} \left(\begin{array}{cc} \text{Re}\;\left(\frac{-\left(\zeta -\alpha \right)\left(\zeta +\alpha \right)\left(\zeta -c\right)\left(\zeta -\frac{1}{c}\right)}{\left(\zeta -\alpha c\right)\left(\zeta -\frac{\alpha }{c}\right)\left(\zeta -1\right)\left(\zeta +1\right)}\right) & 1\\ \text{Im}\;\left(\frac{-\left(\zeta -\alpha \right)\left(\zeta +\alpha \right)\left(\zeta -c\right)\left(\zeta -\frac{1}{c}\right)}{\left(\zeta -\alpha c\right)\left(\zeta -\frac{\alpha }{c}\right)\left(\zeta -1\right)\left(\zeta +1\right)}\right) & 0 \end{array}\right)\left(\begin{array}{c} \xi \\ \eta \end{array}\right)=\left(\begin{array}{c} \text{Re}\;\left(\frac{2\zeta \left(\zeta -c\right)\left(\zeta -\frac{1}{c}\right)}{\left(\zeta -1)(\zeta +1\right)}w\right)\\ \text{Im}\;\left(\frac{2\zeta \left(\zeta -c\right)\left(\zeta -\frac{1}{c}\right)}{\left(\zeta -1)(\zeta +1\right)}w\right) \end{array}\right). \end{array}$$

Because

$$\begin{array}{c} \frac{-\left(\zeta -\alpha \right)\left(\zeta +\alpha \right)\left(\zeta -c\right)\left(\zeta -\frac{1}{c}\right)}{\left(\zeta -\alpha c\right)\left(\zeta -\frac{\alpha }{c}\right)\left(\zeta -1\right)\left(\zeta +1\right)}=-1+\frac{a_{1}}{\zeta -1}+\frac{a_{2}}{\zeta +1}+\frac{a_{3}}{\zeta -\alpha c}+\frac{a_{4}}{\zeta -\frac{\alpha }{c}}, \end{array}$$

with $a_{1},a_{2},a_{3},a_{4}>0$, we have

(57) $$\begin{array}{c} \text{Im}\;\frac{-\left(\zeta -\alpha \right)\left(\zeta +\alpha \right)\left(\zeta -c\right)\left(\zeta -\frac{1}{c}\right)}{\left(\zeta -\alpha c\right)\left(\zeta -\frac{\alpha }{c}\right)\left(\zeta -1\right)\left(\zeta +1\right)}<0,\;\;\text{for}\;\text{Im}\;\zeta >0. \end{array}$$

Thus, the 2 × 2 matrix at the left‐hand side of (56) is invertible, and we get (49). This shows that (ξ,η)↦(s(ξ,η;α),w(ξ,η;α)) is a bijection from $L_{\alpha }$ to $R_{\alpha }^{+}$. This mapping is clearly differentiable, and therefore it is a diffeomorphism. Replacing (s,w)↦(s,−w) in the right‐hand side of (49), we see that the left‐hand side becomes (ξ,η)↦(−ξ,−η). This implies the symmetry s(ξ,η;α)=s(−ξ,−η;α). It remains to prove that $\left(\xi ,\eta \right)\in L_{\alpha }^{l}$ is mapped to a point (s(ξ,η;α),w(ξ,η;α)) lying in the upper half plane of the first sheet. The proof of this claim is splitted in the next two lemmas.

Lemma 1 We have $\text{Im}\;(\frac{2\zeta \left(\zeta -c\right)\left(\zeta -c^{-1}\right)}{\left(\zeta -1)(\zeta +1\right)}Q{\left(\zeta \right)}^{1/2})=0$ if and only if $\zeta \in R\cup \bar{\Sigma_{1}}$.

Consider the function *f* defined by

$$\begin{array}{c} f\left(\zeta \right):=\frac{{\left(\zeta -r_{1}\right)}^{2}{\left(\zeta -r_{2}\right)}^{2}{\left(\zeta -r_{3}\right)}^{2}\left(\zeta -r_{+}\right)\left(\zeta -r_{-}\right)}{{\left(\zeta -1\right)}^{2}{\left(\zeta +1\right)}^{2}{\left(\zeta -\alpha c\right)}^{2}{\left(\zeta -\alpha c^{-1}\right)}^{2}}. \end{array}$$

By the fundamental theorem, for each x∈[0,+∞), there are eight solutions ζ∈C to f(ζ)=x. The claim follows if we show that all these solutions lie on $R\cup \Sigma_{1}$. First, note that the function *f* is positive on the real line, has poles at $-1,\alpha c,\alpha c^{-1},1$, and zeros at $r_{1},r_{2},r_{3}$. Because $-1<r_{1}<\alpha c<r_{2}<\alpha c^{-1}<r_{3}<1$, the equation f(ζ)=x has at least six real solutions (counting multiplicities) for each x∈[0,+∞). Furthermore, f(ζ)→1 as ζ→±∞, *f* has a local minimum at $c^{-1}+R_{1}$, and $f(c^{-1}+R_{1}e^{it})<1$. Therefore, f(ζ)=x has eight solutions on R for each $x\in [f\left(c^{-1}+R_{1}\right),+\infty )$. It remains to show that there are two solutions on $\bar{\Sigma_{1}}$ whenever $x\in [0,f\left(c^{-1}+R_{1}\right)]$. Writing $\zeta =c^{-1}+R_{1}e^{it}\in \gamma_{1}$, t∈[−π,π], some computations show that

$$\begin{array}{c} f\left(c^{-1}+R_{1}e^{it}\right)=\frac{2\left(\cos t-\cos\theta_{1}\right){\left(\cos t+\frac{\alpha^{2}+\left(2-\alpha \right)\sqrt{1-\alpha +\alpha^{2}}}{2(1-\alpha )}\right)}^{2}\cos^{2}\left(\frac{t}{2}\right)}{{\left(\cos t+\frac{\sqrt{1-\alpha +\alpha^{2}}}{1-\alpha }\right)}^{2}{\left(\cos t+\frac{2-\alpha +\alpha^{2}}{2\sqrt{1-\alpha +\alpha^{2}}}\right)}^{2}}. \end{array}$$

So $t\mapsto f(c^{-1}+R_{1}e^{it})$ is even, positive, and decreases from $f(c^{-1}+R_{1})$ to 0 as *t* increases from 0 to θ_1_, which finishes the proof.▪

Lemma 2 Let $\left(s,w\right)\in R_{\alpha }^{+}$ such that $w=Q{\left(s\right)}^{1/2}$ (i.e., (s,w) is in the first sheet). Then, ξ=ξ(s,w;α)<0.

Using (49) together with (57), we infer that ξ has the same sign as

(58) $$-\text{Im}\;\left(\frac{2s\left(s-c\right)\left(s-c^{-1}\right)}{\left(s-1)(s+1\right)}w\right).$$

By Lemma 1, (58) is 0 if and only if $s\in \bar{\Sigma_{1}}$, which implies that the sign of (58) is constant for $s\in C^{+}\setminus \bar{\Sigma_{1}}$. From the expansion

$$\begin{array}{c} -\frac{2s\left(s-c\right)\left(s-c^{-1}\right)}{\left(s-1)(s+1\right)}w=1+\frac{a}{s}+O\left(s^{-2}\right),\;\text{as}\;s\to \infty , \end{array}$$

where a>0, we conclude that (58) is negative for all *s* sufficiently large and lying in $C^{+}$, and the claim follows.▪

This finishes the proof of Proposition 5 and Proposition 4(a). In principle, it is also possible to use (49) to prove parts (b)–(f) of Proposition 4, but it leads to more involved analysis. However, by rearranging the terms in (55), we can find other expressions than (49) for the mapping (s,w)↦(ξ,η) that lead to simpler proofs of (b)–(f). We only sketch the proof of (e). First, we rewrite (55) as

$$\begin{array}{c} \xi +\frac{-\left(\zeta -1\right)\left(\zeta +1\right)\left(\zeta -\alpha c\right)\left(\zeta -\alpha c^{-1}\right)}{\left(\zeta -\alpha \right)\left(\zeta +\alpha \right)\left(\zeta -c\right)\left(\zeta -c^{-1}\right)}\eta =\frac{-2\zeta \left(\zeta -\alpha c\right)\left(\zeta -\alpha c^{-1}\right)}{\left(\zeta -\alpha )(\zeta +\alpha \right)}w, \end{array}$$

which implies

$$\begin{array}{c} \left(\begin{array}{cc} 1 & \text{Re}\;\left(\frac{-\left(\zeta -1\right)\left(\zeta +1\right)\left(\zeta -\alpha c\right)\left(\zeta -\alpha c^{-1}\right)}{\left(\zeta -\alpha \right)\left(\zeta +\alpha \right)\left(\zeta -c\right)\left(\zeta -c^{-1}\right)}\right)\\ 0 & \text{Im}\;\left(\frac{-\left(\zeta -1\right)\left(\zeta +1\right)\left(\zeta -\alpha c\right)\left(\zeta -\alpha c^{-1}\right)}{\left(\zeta -\alpha \right)\left(\zeta +\alpha \right)\left(\zeta -c\right)\left(\zeta -c^{-1}\right)}\right) \end{array}\right)\left(\begin{array}{c} \xi \\ \eta \end{array}\right)=\left(\begin{array}{c} \text{Re}\;\left(\frac{-2\zeta \left(\zeta -\alpha c\right)\left(\zeta -\alpha c^{-1}\right)}{\left(\zeta -\alpha )(\zeta +\alpha \right)}w\right)\\ \text{Im}\;\left(\frac{-2\zeta \left(\zeta -\alpha c\right)\left(\zeta -\alpha c^{-1}\right)}{\left(\zeta -\alpha )(\zeta +\alpha \right)}w\right) \end{array}\right). \end{array}$$

Next, we verify that

$$\begin{array}{c} \text{Im}\;\left(\frac{-\left(\zeta -1\right)\left(\zeta +1\right)\left(\zeta -\alpha c\right)\left(\zeta -\alpha c^{-1}\right)}{\left(\zeta -\alpha \right)\left(\zeta +\alpha \right)\left(\zeta -c\right)\left(\zeta -c^{-1}\right)}\right)>0,\;\text{for}\;\text{Im}\;\zeta >0, \end{array}$$

which implies that η=η(ζ,w;α) has the same sign as

$$\begin{array}{c} \text{Im}\;\left(\frac{-2\zeta \left(\zeta -\alpha c\right)\left(\zeta -\alpha c^{-1}\right)}{\left(\zeta -\alpha )(\zeta +\alpha \right)}w\right). \end{array}$$

Finally, in a similar way as in Lemma 1, we show that this quantity is 0 if and only if $\zeta \in R\cup \Sigma_{\alpha }$, which proves part (e). We omit the proofs of parts (b), (c), (d), and (f).

## 5 ANALYSIS OF THE RH PROBLEM FOR *Y*

To describe the behavior of *Y* as N→+∞, one needs to control the 2 × 2 upper right block of the jumps, which is $A{\left(z\right)}^{2N}z^{-2N}$. To do this, we follow an idea of Duits and Kuijlaars^20^ and proceed with the eigendecomposition of *A*. Then, we use this factorization to perform a first transformation Y↦X on the RH problem.

### 5.1 Eigendecomposition of *A*

The matrix A(z) defined in (12) has the following eigenvalues:

(59) $$\lambda_{1,2}\left(z\right)=\frac{1+\alpha^{2}}{2}\left(1+z\right)\pm \frac{1-\alpha^{2}}{2}\sqrt{\left(z-z_{+}\right)\left(z-z_{-}\right)},\;z\in C\setminus \left[z_{-},z_{+}\right],$$

where the + and − signs read for λ_1_ and λ_2_, respectively, and $z_{+}$ and $z_{-}$ are given by

$$\begin{array}{c} z_{\pm }=\frac{-\left(1+\alpha^{2}\right)\pm 2\sqrt{\alpha (1-\alpha +\alpha^{2})}}{{\left(1-\alpha \right)}^{2}}, \end{array}$$

and satisfy $z_{-}<-1<z_{+}<0$ and $z_{+}z_{-}=1$. We define the square root $\sqrt{\left(z-z_{+}\right)\left(z-z_{-}\right)}$ such that it is analytic in $C\setminus [z_{-},z_{+}]$, with an asymptotic behavior at ∞ given by

$$\begin{array}{c} \sqrt{\left(z-z_{+}\right)\left(z-z_{-}\right)}=z+O\left(1\right),\;\text{as}\;z\to \infty . \end{array}$$

The eigenvectors of *A* are in the columns of the following matrix:

(60) $$\begin{array}{cc} E(z) & =\frac{1}{1+\alpha }\left(\begin{array}{cc} 1+\alpha & 1+\alpha \\ \lambda_{1}\left(z\right)-\left(\alpha^{2}+z\right) & \;\lambda_{2}\left(z\right)-\left(\alpha^{2}+z\right) \end{array}\right)\\ & =\left(\begin{array}{cc} 1 & 1\\ \frac{1-\alpha }{2}\left(1-z+\sqrt{\left(z-z_{+}\right)\left(z-z_{-}\right)}\right) & \frac{1-\alpha }{2}\left(1-z-\sqrt{\left(z-z_{+}\right)\left(z-z_{-}\right)}\right) \end{array}\right), \end{array}$$

and we have the factorization

(61) $$A\left(z\right)=E\left(z\right)\Lambda \left(z\right)E{\left(z\right)}^{-1},$$

where $\Lambda \left(z\right)=\text{diag}(\lambda_{1}\left(z\right),\lambda_{2}\left(z\right))$ is the matrix of eigenvalues. The matrix E(z) is analytic for $z\in C\setminus [z_{-},z_{+}]$, and satisfies

(62) $$\begin{array}{cccc} & E_{+}\left(z\right)=E_{-}\left(z\right)\sigma_{1}, & & z\in (z_{-},z_{+}), \end{array}$$

(63) $$\begin{array}{cccc} & E\left(z\right)=\left(\begin{array}{cc} 1 & 1\\ \frac{1-\alpha +\alpha^{2}}{1-\alpha }+O\left(z^{-1}\right) & \;-(1-\alpha )z+O(1) \end{array}\right) & & \;\text{as}\;z\to \infty , \end{array}$$

where $\sigma_{1}=\left(\begin{array}{cc} 0 & 1\\ 1 & 0 \end{array}\right)$.

### 5.2 First transformation Y↦X

The first transformation of the RH problem diagonalizes the 2 × 2 upper right block of the jumps, and is defined by

(64) $$X\left(z\right)=Y\left(z\right)\left(\begin{array}{cc} E(z) & \;0_{2}\\ 0_{2} & \;E(z) \end{array}\right).$$

Remark 4 By standard arguments,^43^ we have detY≡1. Note however that the Y↦X transformation does not preserve the unit determinant. Indeed, because $\det E\left(z\right)=-\left(1-\alpha \right)\sqrt{\left(z-z_{+}\right)\left(z-z_{-}\right)}$, we have $\det X\left(z\right)={\left(1-\alpha \right)}^{2}\left(z-z_{+}\right)\left(z-z_{-}\right)$.

Using the jumps for *E* given by (62), we verify that *X* satisfies the following RH problem.

#### RH problem for *X*

(a) $X:C\setminus \left(\gamma \cup \left[z_{-},z_{+}\right]\right)\to C^{4\times 4}$ is analytic, where we recall that γ is a closed contour surrounding 0 once in the positive direction.
(b) The jumps for *X* are given by
  (65) $$\begin{array}{cccc} & X_{+}\left(z\right)=X_{-}\left(z\right)\left(\begin{array}{cc} I_{2} & \;\frac{\Lambda^{2N}\left(z\right)}{z^{2N}}\\ 0_{2} & \;I_{2} \end{array}\right), & & \;\text{for}\;z\in \gamma \setminus Z, \end{array}$$
  (66) $$\begin{array}{cccc} & X_{+}\left(z\right)=X_{-}\left(z\right)\left(\begin{array}{cc} \sigma_{1} & \;0_{2}\\ 0_{2} & \;\sigma_{1} \end{array}\right), & & \;\text{for}\;z\in (z_{-},z_{+})\setminus Z, \end{array}$$
  where $Z:=\gamma \cap [z_{-},z_{+}]$. Depending on γ, Z can be the empty set, a finite set, or an infinite set. If Z contains one or several intervals, then on these intervals the jumps are
  $$\begin{array}{cc} & X_{+}\left(z\right)=X_{-}\left(z\right)\left(\begin{array}{cc} \sigma_{1} & \;0_{2}\\ 0_{2} & \;\sigma_{1} \end{array}\right)\left(\begin{array}{cc} I_{2} & \;\frac{\Lambda_{+}^{2N}\left(z\right)}{z^{2N}}\\ 0_{2} & \;I_{2} \end{array}\right)=X_{-}\left(z\right)\left(\begin{array}{cc} I_{2} & \;\frac{\Lambda_{-}^{2N}\left(z\right)}{z^{2N}}\\ 0_{2} & \;I_{2} \end{array}\right)\left(\begin{array}{cc} \sigma_{1} & \;0_{2}\\ 0_{2} & \;\sigma_{1} \end{array}\right). \end{array}$$
(c) As z→∞, we have $X\left(z\right)=\left(I_{4}+O\left(z^{-1}\right)\right)\left(\begin{array}{cc} z^{N}E\left(z\right) & 0_{2}\\ 0_{2} & z^{-N}E\left(z\right) \end{array}\right)$. As $z\to z_{-}$ or as $z\to z_{+}$, $X\left(z\right)=O\left(1\right)\left(\begin{array}{cc} E(z) & 0_{2}\\ 0_{2} & E(z) \end{array}\right)$.

## 6 PROOF OF THEOREM 1

First, we use the factorization of *A* obtained in (61) together with the transformation Y↦X given by (64), to rewrite the formulas (21) and (22) as follows:

(67) $$\begin{array}{cc} & {\left[K(2x+\varepsilon_{x},2y+j,2x+\varepsilon_{x},2y+i)\right]}_{i,j=0}^{1}=\frac{1}{{\left(2\pi i\right)}^{2}}\int_{\gamma }\int_{\gamma }{\left(\begin{array}{cc} \alpha^{2} & \;\alpha \\ w & \;1 \end{array}\right)}^{\varepsilon_{x}}\\ & \;\times E\left(w\right)\frac{\Lambda {\left(w\right)}^{2N-x-\varepsilon_{x}}}{w^{2N-y}}R^{X}\left(w,z\right)\frac{\Lambda {\left(z\right)}^{x}}{z^{y+1}}E{\left(z\right)}^{-1}{\left(\begin{array}{cc} 1 & \;1\\ \alpha z & \;1 \end{array}\right)}^{\varepsilon_{x}}dzdw, \end{array}$$

where $R^{X}$ is given by

(68) $$R^{X}\left(w,z\right)=E^{-1}\left(w\right)R^{Y}\left(w,z\right)E\left(z\right)=\frac{1}{z-w}\left(\begin{array}{cc} 0_{2} & I_{2} \end{array}\right)X^{-1}\left(w\right)X\left(z\right)\left(\begin{array}{c} I_{2}\\ 0_{2} \end{array}\right).$$

The property (23) of $R^{Y}$ implies the following reproducing property for $R^{X}$:

(69) $$\frac{1}{2\pi i}\int_{\gamma }P\left(w\right)E\left(w\right)\frac{\Lambda {\left(w\right)}^{2N}}{w^{2N}}R^{X}\left(w,z\right)dw=P\left(z\right)E\left(z\right),$$

for every 2 × 2 matrix valued polynomial *P* of degree ≤N−1.

Now, we introduce the Riemann surface M associated to the eigenvalues and eigenvectors of *A*. This Riemann surface is of genus 0 and therefore there is a one‐to‐one map between it and the Riemann sphere (called the ζ‐plane).

### 6.1 The Riemann surface M and the ζ‐plane

The Riemann surface M is defined by

(70) $$M=\{\left(z,y\right)\in C\times C:y^{2}=\left(z-z_{+}\right)\left(z-z_{-}\right)\},$$

and has genus zero. We represent it as a two‐sheeted covering of the *z*‐plane glued along $\left[z_{-},z_{+}\right]$. On the first sheet we require y=z+O(1) as z→∞, and on the second sheet we require y=−z+O(1) as z→∞. To shorten the notations, a point (z,y) lying on the Riemann surface will simply be denoted by *z* when there is no confusion, that is, we will omit the *y*‐coordinate. If we want to emphasize that the point (z,y) is on the *j*‐th sheet, j∈{1,2}, then we will use the notation $z^{\left(j\right)}$. With this convention, the two points at infinity are denoted by ∞^(1)^ and ∞^(2)^. The function *y* satisfies

(71) $$\begin{array}{cccc} & y\left(\frac{1}{\alpha^{\left(2\right)}}\right)=-\frac{1+\alpha^{2}}{\alpha (1-\alpha )}, & & y\left(\alpha^{\left(2\right)}\right)=-\frac{1+\alpha^{2}}{1-\alpha },\\ & y(0^{\left(2\right)})=-1, & & y(0^{\left(1\right)})=1. \end{array}$$

The functions $\lambda_{1}\left(z\right)$ and $\lambda_{2}\left(z\right)$ are defined on the *z*‐plane (see (59)), and together they define a function λ on M as follows:

(72) $$\lambda \left((z,y)\right)=\left\{\begin{array}{cc} \lambda_{1}\left(z\right), & \;\text{if}\;(z,y)\;\text{is}\;\text{on}\;\text{the}\;\text{first}\;\text{sheet},\\ \lambda_{2}\left(z\right), & \;\text{if}\;(z,y)\;\text{is}\;\text{on}\;\text{the}\;\mathrm{second}\;\text{sheet}. \end{array}\right.$$

This is a meromorphic function on M with two simple poles at ∞^(1)^ and ∞^(2)^ and no other poles. Using (71), we verify that λ has two simple zeros at α^(2)^ and $\frac{1}{\alpha^{\left(2\right)}}$, and because M has genus 0, λ has no other zeros. From (62), the matrix *E* can also be extended to the full Riemann surface as follows:

$$\begin{array}{cc} E\left((z,y)\right) & =\left(\begin{array}{cc} 1 & 1\\ \frac{1-\alpha }{2}\left(1-z+y\right) & \;\frac{1-\alpha }{2}\left(1-z-y\right) \end{array}\right)\\ & =\left\{\begin{array}{cc} E(z), & \text{if}\;(z,y)\;\text{is}\;\text{on}\;\text{the}\;\text{first}\;\text{sheet},\\ E\left(z\right)\sigma_{1}, & \text{if}\;(z,y)\;\text{is}\;\text{on}\;\text{the}\;\mathrm{second}\;\text{sheet}. \end{array}\right. \end{array}$$

The function ζ=ζ(z), defined by

(73) $$\zeta =\;\frac{2z-(z_{+}+z_{-})+2y}{z_{+}-z_{-}},$$

is a conformal and bijective map from M to the Riemann sphere. The first sheet of M is mapped by (73) to the subset {ζ∈C∪{∞}:|ζ|>1} of the ζ‐plane, and the second sheet is mapped to {ζ∈C∪{∞}:|ζ|<1}. The inverse function z=z(ζ) is given by

(74) $$z=\frac{z_{+}+z_{-}}{2}+\frac{z_{+}-z_{-}}{4}\left(\zeta +\zeta^{-1}\right),$$

where *z* is on the first sheet if |ζ|>1 and on the second sheet if |ζ|<1. By definition, the above function z(ζ) vanishes at ζ(0^(1)^) and ζ(0^(2)^). Because it has simple poles at ζ=0 and ζ=∞, and because $z\left(\zeta \right)=\frac{z_{+}-z_{-}}{4}\zeta +O\left(1\right)$ as ζ→∞, (74) can be rewritten as

(75) $$z=\frac{z_{+}-z_{-}}{4\zeta }\left(\zeta -\zeta \left(0^{\left(1\right)}\right)\right)\left(\zeta -\zeta \left(0^{\left(2\right)}\right)\right).$$

The functions z(ζ) and ζ(z) satisfy

$$\begin{array}{cccccccc} & z\left(1\right)=z_{+}, & & z\left(-1\right)=z_{-}, & & z\left(\infty \right)=\infty^{\left(1\right)}, & & z\left(0\right)=\infty^{\left(2\right)},\\ & \zeta (z_{+})=1, & & \zeta (z_{-})=-1, & & \zeta (\infty^{\left(1\right)})=\infty , & & \zeta (\infty^{\left(2\right)})=0. \end{array}$$

Also, we note that as z∈M, Imz=0, $z\notin (z_{-},z_{+})$, follows the straight line segments $\left[\infty^{\left(1\right)},z_{-}\right]$, $\left[z_{-},\infty^{\left(2\right)}\right]$, $\left[\infty^{\left(2\right)},z_{+}\right]$, $\left[z_{+},\infty^{\left(1\right)}\right]$, the function ζ(z) increases from −∞ to +∞. In particular, we have

$$\begin{array}{c} \zeta \left(z_{-}\right)<\zeta \left(\infty^{\left(2\right)}\right)<\zeta \left(\frac{1}{\alpha^{\left(2\right)}}\right)<\zeta \left(\alpha^{\left(2\right)}\right)<\zeta \left(0^{\left(2\right)}\right)<\zeta \left(z_{+}\right)<\zeta \left(0^{\left(1\right)}\right). \end{array}$$

The following identities will be useful later, and can be verified by direct computations:

(76) $$\begin{array}{cc} & y=\frac{z_{+}-z_{-}}{4}\left(\zeta -\zeta^{-1}\right),\;\frac{dz}{y}=\frac{d\zeta }{\zeta }, \end{array}$$

(77) $$\begin{array}{cc} & \lambda =\frac{z_{+}-z_{-}}{4\zeta }\left(\zeta -\zeta \left(\frac{1}{\alpha^{\left(2\right)}}\right)\right)\left(\zeta -\zeta \left(\alpha^{\left(2\right)}\right)\right), \end{array}$$

(78) $$\begin{array}{cc} & \frac{d\lambda }{dz}=\frac{\zeta^{2}-\alpha^{2}}{\zeta^{2}-1}, \end{array}$$

(79) $$\begin{array}{cc} & \frac{dz}{d\zeta }=\frac{z_{+}-z_{-}}{4\zeta }\left(\zeta -\zeta^{-1}\right). \end{array}$$

We define *c* by

$$\begin{array}{c} c=\frac{z_{+}-z_{-}}{-\left(z_{+}+z_{-}\right)+2\sqrt{z_{+}z_{-}}}=\sqrt{\frac{\alpha }{1-\alpha +\alpha^{2}}}<1. \end{array}$$

From straightforward calculations using (73), we have

$$\begin{array}{cccc} & \zeta (\frac{1}{\alpha^{\left(2\right)}})=\alpha c, & & \zeta \left(\alpha^{\left(2\right)}\right)=\alpha c^{-1},\\ & \zeta (0^{\left(2\right)})=c, & & \zeta \left(0^{\left(1\right)}\right)=c^{-1}, \end{array}$$

and

(80) $$\begin{array}{c} \lambda \left(z\right)-\alpha^{2}-z=\frac{1+\alpha^{3}}{1-\alpha }\frac{\zeta -c}{\zeta }. \end{array}$$

### 6.2 The reproducing kernel $R^{M}$

For $w^{\left(j\right)}$ on the *j*‐th sheet of M and $z^{\left(k\right)}$ on the *k*‐th sheet, we define $R^{M}\left(w^{\left(j\right)},z^{\left(k\right)}\right)$ by

(81) $$R^{M}\left(w^{\left(j\right)},z^{\left(k\right)}\right)=y\left(w^{\left(j\right)}\right)e_{j}^{T}R^{X}\left(w,z\right)e_{k},$$

where $e_{1}=\left(\begin{array}{c} 1\\ 0 \end{array}\right)$ and $e_{2}=\left(\begin{array}{c} 0\\ 1 \end{array}\right)$. Note that $R^{M}:M_{*}\times M_{*}\to C$ is scalar valued, with $M_{*}=M\setminus \left\{\infty^{\left(1\right)},\infty^{\left(2\right)}\right\}$. It is convenient for us to consider formal sums of points on M, which are called *divisors* in the literature. More precisely, a divisor *D* can be written in the form

$$\begin{array}{c} D=\sum_{j=1}^{k}n_{j}z_{j},\;k\geq 1,\;n_{j}\in Z,\;z_{j}\in M, \end{array}$$

and we say that D≥0 if $n_{1},\ldots ,n_{k}\geq 0$. The divisor of a nonzero meromorphic function *f* on M is defined by

$$\begin{array}{c} \text{div}\left(f\right):=n_{1}z_{1}+\cdots +n_{k_{1}}z_{k_{1}}-n_{k_{1}+1}z_{k_{1}+1}-\cdots -n_{k_{2}}z_{k_{2}}, \end{array}$$

where $z_{1},\ldots ,z_{k_{1}}$ are the zeros of *f* of multiplicities $n_{1},\ldots ,n_{k_{1}}$, respectively, and $z_{k_{1}+1},\ldots ,z_{k_{2}}$ are the poles of *f* of order $n_{k_{1}+1},\ldots ,n_{k_{2}}$, respectively. Given a divisor *D*, we define L(−D) as the vector space of meromorphic functions on M given by

$$\begin{array}{c} L(-D)=\{f:\text{div}(f)\geq -D\;\text{or}\;f\equiv 0\}. \end{array}$$

The following divisors will play an important role:

$$\begin{array}{cc} & D_{N}=\left(N-1\right)\cdot \infty^{\left(1\right)}+N\cdot \infty^{\left(2\right)},\\ & D_{N}^{*}=N\cdot \infty^{\left(1\right)}+\left(N-1\right)\cdot \infty^{\left(2\right)}. \end{array}$$

Thus, $L_{N}:=L\left(-D_{N}\right)$ is the vector space of meromorphic functions on M, with poles at ∞^(1)^ and ∞^(2)^ only, such that the pole at ∞^(1)^ is of order at most N−1, and the pole at ∞^(2)^ is of order at most *N*. Similarly we define $L_{N}^{*}=L\left(-D_{N}^{*}\right)$. From the Riemann–Roch theorem, we have

$$\begin{array}{c} \text{dim}\;L_{N}=\text{dim}\;L_{N}^{*}=2N, \end{array}$$

because there is no holomorphic differential (other than the zero differential) on a Riemann surface of genus 0.

Lemma 3 We have

(a) $z\mapsto R^{M}\left(w,z\right)\in L_{N}$ for every $w\in M_{*}$,
(b) $w\mapsto R^{M}\left(w,z\right)\in L_{N}^{*}$ for every $z\in M_{*}$,
(c) $R^{M}$ is a reproducing kernel for $L_{N}$ in the sense that
  (82) $$\frac{1}{2\pi i}\int_{\gamma_{M}}f\left(w\right)\frac{\lambda^{2N}\left(w\right)}{w^{2N}}R^{M}\left(w,z\right)\frac{dw}{y(w)}=f\left(z\right)$$
  for every $f\in L_{N}$, where $\gamma_{M}$ is a closed contour surrounding once 0^(1)^ and 0^(2)^ on the Riemann surface M in the positive direction (in particular $\gamma_{M}$ visits both sheets).

Using the definitions of $R^{M}$ and $R^{X}$ given by (81) and (68), we can rewrite $R^{M}$ as

(83) $$R^{M}\left(w,z\right)=\frac{\sum_{j=1}^{4}g_{j}\left(w\right)f_{j}\left(z\right)}{z-w},\;w,z\in M_{*},$$

where

$$f_{j}\left(z\right)=\left\{\begin{array}{cc} X_{j1}\left(z\right), & \;\text{if}\;z=z^{\left(1\right)},\\ X_{j2}\left(z\right), & \;\text{if}\;z=z^{\left(2\right)}, \end{array}\right.$$

and

(84) $$\begin{array}{c} g_{j}\left(w\right)=y\left(w\right)\left\{\begin{array}{cc} {\left(X^{-1}\right)}_{3j}\left(w\right), & \;\text{if}\;w=w^{\left(1\right)},\\ {\left(X^{-1}\right)}_{4j}\left(w\right), & \;\text{if}\;w=w^{\left(2\right)}. \end{array}\right. \end{array}$$

From properties (a) and (b) of the RH problem for *X*, the functions $f_{j}$ are analytic in $M_{*}$. By combining the large *z* asymptotics of E(z) (given by (63)) with property (c) of the RH problem for *X*, we obtain

$$\begin{array}{c} X\left(z\right)\left(\begin{array}{c} I_{2}\\ 0_{2} \end{array}\right)=\left(\begin{array}{cc} O(z^{N}) & \;O(z^{N})\\ O(z^{N}) & \;O(z^{N+1})\\ O(z^{N-1}) & \;O(z^{N})\\ O(z^{N-1}) & \;O(z^{N}) \end{array}\right),\;\text{as}\;z\to \infty , \end{array}$$

from which we conclude that the functions $f_{j}$'s have poles of order at most *N* at ∞^(1)^ and at most N+1 at ∞^(2)^. Therefore, we have shown that

$$\begin{array}{c} f_{j}\in L\left(-\left(D_{N}+\infty^{\left(1\right)}+\infty^{\left(2\right)}\right)\right),\;j=1,2,3,4. \end{array}$$

The numerator in (83) is, for each fixed $w\in M_{*}$ a linear combination of the functions $f_{j}$, so belong to $L(-\left(D_{N}+\infty^{\left(1\right)}+\infty^{\left(2\right)}\right))$ as a function of *z*. By definitions of $R^{M}$ and $R^{X}$, the numerator vanishes for $z=w^{\left(1\right)}$ and for $z=w^{\left(2\right)}$. Thus, the division by z−w in (83) does not introduce any poles, but it reduces the order of the poles at ∞^(1)^ and ∞^(2)^ by one, and therefore $z\mapsto R^{M}\left(w,z\right)\in L_{N}$ as claimed in part (a). Now, we turn to the proof of part (b). First, we note that

$$\begin{array}{c} E{\left(w\right)}^{-1}=\frac{-\frac{1}{1-\alpha }}{\sqrt{\left(w-z_{+}\right)\left(w-z_{-}\right)}}\left(\begin{array}{cc} \frac{1-\alpha }{2}\left(1-w-\sqrt{\left(w-z_{+}\right)\left(w-z_{-}\right)}\right) & -1\\ -\frac{1-\alpha }{2}\left(1-w+\sqrt{\left(w-z_{+}\right)\left(w-z_{-}\right)}\right) & 1 \end{array}\right). \end{array}$$

Therefore, because detY≡1, by using condition (c) of the RH problem for *X*, we have

$$\begin{array}{c} X^{-1}\left(w\right)=\left(\begin{array}{cc} E^{-1}\left(w\right) & 0_{2}\\ 0_{2} & \;E^{-1}\left(w\right) \end{array}\right)\left(\begin{array}{cc} O(1) & O(1)\\ O(1) & O(1) \end{array}\right)\;\text{as}\;w\to z_{*}\in \left\{z_{+},z_{-}\right\}, \end{array}$$

and we conclude from (84) that the functions $g_{j}$ are also analytic in $M_{*}$. On the other hand, by using the asymptotics $Y\left(w\right)=I_{4}+O\left(w^{-1}\right)$ as w→∞ together with the fact that detY≡1, we can obtain asymptotics for $X^{-1}\left(w\right)$ as w→∞ using (64). After some simple computations, we get

$$y\left(w\right)\left(\begin{array}{cc} 0_{2} & I_{2} \end{array}\right)X^{-1}\left(w\right)=\left(\begin{array}{cccc} O(w^{N}) & \;O(w^{N}) & \;O(w^{N+1}) & \;O(w^{N})\\ O(w^{N-1}) & \;O(w^{N-1}) & \;O(w^{N}) & \;O(w^{N}) \end{array}\right),$$

from which it follows that

$$\begin{array}{c} g_{j}\in L\left(-\left(D_{N}^{*}+\infty^{\left(1\right)}+\infty^{\left(2\right)}\right)\right),\;j=1,2,3,4. \end{array}$$

We conclude the proof of part (b) as in part (a), by noting that $R^{M}\left(w,z\right)$ in (83) has no pole at z=w (on any sheet). Finally, let us take $P\left(w\right)=p\left(w\right)e_{1}^{T}=p\left(w\right)\left(\begin{array}{cc} 1 & 0 \end{array}\right)$ in (69), with *p* a scalar polynomial satisfying degp≤N−1. Because $e_{1}^{T}E\left(w\right)=\left(\begin{array}{cc} 1 & 1 \end{array}\right)=e_{1}^{T}+e_{2}^{T}$, it gives

$$\begin{array}{c} p\left(z\right)\left(\begin{array}{cc} 1 & 1 \end{array}\right)=\frac{1}{2\pi i}\int_{\gamma }p\left(w\right)\left(e_{1}^{T}+e_{2}^{T}\right)\frac{\Lambda {\left(w\right)}^{2N}}{w^{2N}}R^{X}\left(w,z\right)dw. \end{array}$$

By multiplying the above from the right by $e_{k}$, we obtain

$$\begin{array}{cc} p(z)= & \;\frac{1}{2\pi i}\int_{\gamma }p\left(w\right)\frac{\lambda_{1}{\left(w\right)}^{2N}}{w^{2N}}e_{1}^{T}R^{X}\left(w,z\right)e_{k}dw+\frac{1}{2\pi i}\int_{\gamma }p\left(w\right)\frac{\lambda_{2}{\left(w\right)}^{2N}}{w^{2N}}e_{2}^{T}R^{X}\left(w,z\right)e_{k}dw. \end{array}$$

We denote γ^(1)^ and γ^(2)^ for the projections of γ on the first and second sheets of M, respectively. Using (81), the above can be rewritten as

$$\begin{array}{cc} p(z)= & \;\frac{1}{2\pi i}\int_{\gamma^{\left(1\right)}}p\left(w\right)\frac{\lambda {\left(w\right)}^{2N}}{w^{2N}}R^{M}\left(w,z^{\left(k\right)}\right)\frac{dw}{y(w)}+\frac{1}{2\pi i}\int_{\gamma^{\left(2\right)}}p\left(w\right)\frac{\lambda {\left(w\right)}^{2N}}{w^{2N}}R^{M}\left(w,z^{\left(k\right)}\right)\frac{dw}{y(w)}, \end{array}$$

for every z∈C and for any k∈{1,2}. The two integrals combine to one integral over a contour $\gamma_{M}$ surrounding both 0^(1)^ and 0^(2)^ on M in the positive direction, and thus

(85) $$\begin{array}{c} p\left(z\right)=\frac{1}{2\pi i}\int_{\gamma_{M}}p\left(w\right)\frac{\lambda {\left(w\right)}^{2N}}{w^{2N}}R^{M}\left(w,z\right)\frac{dw}{y(w)},\;\text{deg}\;p\leq N-1, \end{array}$$

for every $z\in M_{*}$. Let us now take $P\left(w\right)=p\left(w\right)e_{2}^{T}=p\left(w\right)\left(\begin{array}{cc} 0 & 1 \end{array}\right)$ in (69), and note that

$$e_{2}^{T}E\left(w\right)=\frac{1}{1+\alpha }\left(\begin{array}{cc} \lambda_{1}\left(w\right)-\alpha^{2}-w & \lambda_{2}\left(w\right)-\alpha^{2}-w \end{array}\right).$$

The two above entries together define the meromorphic function $w\in M\mapsto \frac{1}{1+\alpha }\left(\lambda \left(w\right)-\alpha^{2}-w\right)$ on M. By proceeding in a similar way as for (85), we obtain this time

$$\begin{array}{c} p\left(z\right)\left(\lambda \left(z\right)-\left(\alpha^{2}+z\right)\right)=\frac{1}{2\pi i}\int_{\gamma_{M}}p\left(w\right)\left(\lambda \left(w\right)-\left(\alpha^{2}+w\right)\right)\frac{\lambda {\left(w\right)}^{2N}}{w^{2N}}R^{M}\left(w,z\right)\frac{dw}{y(w)}, \end{array}$$

for all scalar polynomials *p* with degp≤N−1 and for all $z\in M_{*}$. Therefore, for any function *f* in the form

$$\begin{array}{c} f\left(z\right)=p_{1}\left(z\right)+p_{2}\left(z\right)\left(\lambda \left(z\right)-\alpha^{2}-z\right) \end{array}$$

with *p*_1_, *p*_2_ two polynomials of degree ≤N−1, we have proved that

$$\begin{array}{c} f\left(z\right)=\frac{1}{2\pi i}\int_{\gamma_{M}}f\left(w\right)\frac{\lambda {\left(w\right)}^{2N}}{w^{2N}}R^{M}\left(w,z\right)\frac{dw}{y(w)}. \end{array}$$

Let $L:=\{f:f\left(z\right)=p_{1}\left(z\right)+p_{2}\left(z\right)\left(\lambda \left(z\right)-\alpha^{2}-z\right)\;\text{with}\;p_{1},p_{2}\;\text{two}\;\text{polynomials}\;\text{of}\;\text{degree}\;\leq N-1\}$. Because $z\mapsto \lambda -\alpha^{2}-z$ has a simple pole at ∞^(2)^ (and no other poles), we conclude that $L\subseteq L_{N}$. Note also that $\text{dim}\;L=\text{dim}\;L_{N}=2N$, and thus we have $L=L_{N}$. This finishes the proof.▪

### 6.3 The reproducing kernel $R^{U}$

To ease the notations, we define z=z(ζ) and w=w(ω) by

(86) $$\begin{array}{cc} & z=\frac{z_{+}+z_{-}}{2}+\frac{z_{+}-z_{-}}{4}\left(\zeta +\zeta^{-1}\right),\;\;\;\zeta \in C\cup \left\{\infty \right\},\;z\in M, \end{array}$$

(87) $$\begin{array}{cc} & w=\frac{z_{+}+z_{-}}{2}+\frac{z_{+}-z_{-}}{4}\left(\omega +\omega^{-1}\right),\;\omega \in C\cup \left\{\infty \right\},\;w\in M, \end{array}$$

with the same convention as in (74), that is, *z* (resp. *w*) is on the first sheet if |ζ|>1 (resp. |ω|>1), and on the second sheet if |ζ|<1 (resp. |ω|<1). We define $R^{U}$ in terms of $R^{M}$ as follows:

(88) $$R^{U}\left(\omega ,\zeta \right)=\omega^{N-1}\zeta^{N}R^{M}\left(w\left(\omega \right),z\left(\zeta \right)\right).$$

Proposition 6 Let *W* and *c* be defined as in (24). $R^{U}$ is a bivariate polynomial of degree ≤2N−1 in both ω and ζ. It satisfies

(89) $$\frac{1}{2\pi i}\int_{\gamma_{C}}p\left(\omega \right)W\left(\omega \right)R^{U}\left(\omega ,\zeta \right)d\omega =p\left(\zeta \right)$$

for every scalar polynomial *p* of degree ≤2N−1, where $\gamma_{C}$ is a closed curve in the complex plane going around *c* and $c^{-1}$ once in the positive direction, but not going around 0.

From part (a) of Lemma 3, for each $w\in M_{*}$, the function $z\mapsto R^{M}\left(w,z\right)$ is meromorphic on M, with a pole of order at most N−1 at ∞^(1)^ and a pole of order at most *N* at ∞^(2)^. Because $z\left(0\right)=\infty^{\left(2\right)}$ and $z\left(\infty \right)=\infty^{\left(1\right)}$, we conclude that for each ω∈C, the function $\zeta \mapsto R^{M}\left(w\left(\omega \right),z\left(\zeta \right)\right)$ is meromorphic on C∪{∞}, with a pole of order at most N−1 at ∞ and another pole of order at most *N* at 0. Therefore, for each ω∈C, the function $\zeta \mapsto R^{U}\left(\omega ,\zeta \right)$ is a polynomial of degree at most 2N−1. From part (b) of Lemma 3, we conclude similarly that for each ζ∈C, the function $\omega \mapsto R^{U}\left(\omega ,\zeta \right)$ is a polynomial of degree at most 2N−1. So we have proved that $R^{U}$ is a bivariate polynomial of degree ≤2N−1 in both ω and ζ.

Now, we turn to the proof of (89). It can be directly verified from (87) (see also (75)) that $\omega \left(0^{\left(1\right)}\right)=c^{-1}$, $\omega (0^{\left(2\right)})=c$, $\left(\partial_{\omega }w\right)\left(c^{-1}\right)>0$, and $\left(\partial_{\omega }w\right)\left(c\right)<0$. In particular, the map w↦ω(w) is conformal in small neighborhoods of 0^(1)^ and 0^(2)^. Because conformal maps preserve orientation, the curve $\gamma_{M}$ which surrounds both 0^(1)^ and 0^(2)^ once in the positive direction, is mapped by w↦ω(w) onto a curve $\gamma_{C}$ on the complex plane, which surrounds *c* and $c^{-1}$ once in the positive direction. Furthermore, because $\omega (\infty^{\left(2\right)})=0$, the curve $\gamma_{C}$ does not surround 0. By changing variables (w,z)↦(ω,ζ) in (82), and by using (75), (76), and (77), we obtain

$$\begin{array}{ccc} f(z(\zeta )) & = & \frac{1}{2\pi i}\int_{\gamma_{C}}f\left(w\left(\omega \right)\right)\frac{\lambda^{2N}\left(w\left(\omega \right)\right)}{w{\left(\omega \right)}^{2N}}R^{M}\left(w\left(\omega \right),z\left(\zeta \right)\right)\frac{dw(\omega )}{y(w(\omega ))}\\ & = & \frac{1}{2\pi i}\int_{\gamma_{C}}f\left(w\left(\omega \right)\right){\left(\frac{\left(\omega -\alpha c\right)\left(\omega -\alpha c^{-1}\right)}{\left(\omega -c\right)\left(\omega -c^{-1}\right)}\right)}^{2N}R^{M}\left(w\left(\omega \right),z\left(\zeta \right)\right)\frac{d\omega }{\omega }, \end{array}$$

for every $f\in L_{N}$. Because $f\in L_{N}$, the function ζ↦f(z(ζ)) is meromorphic on the Riemann sphere, with a pole of degree at most *N* at ζ=0 and a pole of degree at most N−1 at ζ=∞. In other words, $\zeta \mapsto \zeta^{N}f\left(z\left(\zeta \right)\right)=:p\left(\zeta \right)$ is a polynomial of degree at most 2N−1. By multiplying the above equality by $\zeta^{N}$, we thus have

$$p\left(\zeta \right)=\frac{1}{2\pi i}\int_{\gamma_{C}}\frac{p(\omega )}{\omega^{N}}{\left(\frac{\left(\omega -\alpha c\right)\left(\omega -\alpha c^{-1}\right)}{\left(\omega -c\right)\left(\omega -c^{-1}\right)}\right)}^{2N}R^{M}\left(w\left(\omega \right),z\left(\zeta \right)\right)\zeta^{N}\frac{d\omega }{\omega }.$$

We obtain the claim after substituting (88) in the above expression.▪

Now, we prove formula (28), which expresses $R^{U}$ in terms of the solution *U* to the 2 × 2 RH problem presented in Section 3.1.

Proposition 7 The reproducing kernel $R^{U}$ defined by (88) can be rewritten in terms of *U* as follows:

(90) $$R^{U}\left(\omega ,\zeta \right)=\frac{1}{\zeta -\omega }\left(\begin{array}{cc} 0 & 1 \end{array}\right)U^{-1}\left(\omega \right)U\left(\zeta \right)\left(\begin{array}{c} 1\\ 0 \end{array}\right).$$

By Ref. 20, lemma 4.6(c), there is a unique bivariate polynomial $R^{U}$ of degree ≤2N−1 in both ω and ζ which satisfies (89). Therefore, it suffices to first replace $R^{U}$ in the left‐hand side of (89) by

$$\begin{array}{c} \frac{1}{\zeta -\omega }\left(\begin{array}{cc} 0 & 1 \end{array}\right)U^{-1}\left(\omega \right)U\left(\zeta \right)\left(\begin{array}{c} 1\\ 0 \end{array}\right), \end{array}$$

and then to verify that (89) still holds with this replacement. The rest of the proof goes exactly as in the proof of Ref. 20, proposition 4.9, so we omit it.▪

### 6.4 Proof of formula (29)

Now, using the results of Sections 6.1, 6.2, and 6.3, we give a proof for formula (29). From (21) and (22), for x∈{1,…,2N−1}, y∈Z, and $\varepsilon_{x}\in \left\{0,1\right\}$, we have

(91) $$\begin{array}{cc} & {\left[K(2x+\varepsilon_{x},2y+j,2x+\varepsilon_{x},2y+i)\right]}_{i,j=0}^{1}\\ & =\frac{1}{{\left(2\pi i\right)}^{2}}\int_{\gamma }\int_{\gamma }{\left(\begin{array}{cc} \alpha^{2} & \alpha \\ w & 1 \end{array}\right)}^{\varepsilon_{x}}\frac{A{\left(w\right)}^{2N-x-\varepsilon_{x}}}{w^{2N-y}}R^{Y}\left(w,z\right)\frac{A{\left(z\right)}^{x}}{z^{y+1}}{\left(\begin{array}{cc} 1 & 1\\ \alpha z & 1 \end{array}\right)}^{\varepsilon_{x}}dzdw, \end{array}$$

where γ is a closed contour surrounding 0 once in the positive direction. The proof consists of using the successive transformations $R^{Y}\mapsto R^{X}\mapsto R^{M}\mapsto R^{U}$. We first use the eigendecomposition 61 of *A* and the $R^{Y}\mapsto R^{X}$ transformation given in (68) to rewrite (91) as

(92) $$\begin{array}{cc} & {\left[K(2x+\varepsilon_{x},2y+j,2x+\varepsilon_{x},2y+i)\right]}_{i,j=0}^{1}\\ & =\frac{1}{{\left(2\pi i\right)}^{2}}\int_{\gamma }\int_{\gamma }{\left(\begin{array}{cc} \alpha^{2} & \alpha \\ w & 1 \end{array}\right)}^{\varepsilon_{x}}E\left(w\right)\frac{\Lambda {\left(w\right)}^{2N-x-\varepsilon_{x}}}{w^{2N-y}}R^{X}\left(w,z\right)\frac{\Lambda {\left(z\right)}^{x}}{z^{y+1}}E{\left(z\right)}^{-1}{\left(\begin{array}{cc} 1 & 1\\ \alpha z & 1 \end{array}\right)}^{\varepsilon_{x}}dzdw. \end{array}$$

Using (60), we can write E(w) and $E{\left(z\right)}^{-1}$ as

(93) $$\begin{array}{cc} E(w) & \;=\left(\begin{array}{cc} 1 & 1\\ \frac{\lambda \left(w^{\left(1\right)}\right)-\alpha^{2}-w}{1+\alpha } & \;\frac{\lambda \left(w^{\left(2\right)}\right)-\alpha^{2}-w}{1+\alpha } \end{array}\right),\;w\in C, \end{array}$$

(94) $$\begin{array}{cc} E{\left(z\right)}^{-1} & \;=\frac{1}{1-\alpha }\left(\begin{array}{cc} \frac{(1+\alpha^{3})z}{y\left(z^{\left(1\right)}\right)\left(\lambda \left(z^{\left(1\right)}\right)-\alpha^{2}-z\right)} & \frac{1}{y(z^{\left(1\right)})}\\ \frac{(1+\alpha^{3})z}{y\left(z^{\left(2\right)}\right)\left(\lambda \left(z^{\left(2\right)}\right)-\alpha^{2}-z\right)} & \frac{1}{y(z^{\left(2\right)})} \end{array}\right),\;z\in C, \end{array}$$

where we have also used the relation

$$\left(\lambda_{1}-\alpha^{2}-z\right)\left(\lambda_{2}-\alpha^{2}-z\right)=-\left(1+\alpha \right)\left(1+\alpha^{3}\right)z$$

to obtain (94). The identities (93) and (94) allow to rewrite the integrand of (92) by noting that

$$\begin{array}{cc} & E\left(w\right)\frac{\Lambda {\left(w\right)}^{2N-x-\varepsilon_{x}}}{w^{2N-y}}R^{X}\left(w,z\right)\frac{\Lambda {\left(z\right)}^{x}}{z^{y+1}}E{\left(z\right)}^{-1}=\sum_{j,k=1}^{2}\left(\begin{array}{c} 1\\ \frac{\lambda \left(w^{\left(j\right)}\right)-\alpha^{2}-w}{1+\alpha } \end{array}\right)\lambda {\left(w^{\left(j\right)}\right)}^{2N-x-\varepsilon_{x}}\\ & \times e_{j}^{T}\frac{y\left(w^{\left(j\right)}\right)R^{X}\left(w,z\right)}{w^{2N-y}z^{y+1}}e_{k}\lambda {\left(z^{\left(k\right)}\right)}^{x}\left(\begin{array}{cc} \frac{(1+\alpha^{3})z}{\left(1-\alpha \right)(\lambda \left(z^{\left(k\right)}\right)-\alpha^{2}-z)} & \frac{1}{1-\alpha } \end{array}\right)\frac{1}{y\left(w^{\left(j\right)}\right)y\left(z^{\left(k\right)}\right)}. \end{array}$$

Therefore, using also the $R^{X}\mapsto R^{M}$ transformation given by (81), we obtain

$$\begin{array}{cc} & {\left[K(2x+\varepsilon_{x},2y+j,2x+\varepsilon_{x},2y+i)\right]}_{i,j=0}^{1}=\frac{1}{{\left(2\pi i\right)}^{2}}\int_{\gamma_{M}}\int_{\gamma_{M}}{\left(\begin{array}{cc} \alpha^{2} & \alpha \\ w & 1 \end{array}\right)}^{\varepsilon_{x}}\left(\begin{array}{c} 1\\ \frac{\lambda \left(w\right)-\alpha^{2}-w}{1+\alpha } \end{array}\right)\\ & \lambda {\left(w\right)}^{2N-x-\varepsilon_{x}}\frac{R^{M}\left(w,z\right)}{w^{2N-y}z^{y+1}}\lambda {\left(z\right)}^{x}\left(\begin{array}{cc} \frac{(1+\alpha^{3})z}{\left(1-\alpha \right)(\lambda \left(z\right)-\alpha^{2}-z)} & \frac{1}{1-\alpha } \end{array}\right){\left(\begin{array}{cc} 1 & 1\\ \alpha z & 1 \end{array}\right)}^{\varepsilon_{x}}\frac{dzdw}{y(w)y(z)}, \end{array}$$

where $\gamma_{M}$ is a closed contour surrounding once 0^(1)^ and 0^(2)^ on M in the positive direction. By performing the change of variables w=w(ω) and z=z(ζ) as in (86) and (87), using the factorization (75) and (77), the identity (76), and also the $R^{M}\mapsto R^{U}$ transformation given by (88), we get

(95) $$\begin{array}{cc} & {\left[K(2x+\varepsilon_{x},2y+j,2x+\varepsilon_{x},2y+i)\right]}_{i,j=0}^{1}=\frac{1}{{\left(2\pi i\right)}^{2}}\int_{\gamma_{C}}\int_{\gamma_{C}}{\left(\begin{array}{cc} \alpha^{2} & \alpha \\ w & 1 \end{array}\right)}^{\varepsilon_{x}}\left(\begin{array}{c} 1\\ \frac{\lambda \left(w\right)-\alpha^{2}-w}{1+\alpha } \end{array}\right)\\ & {\left(\frac{\left(\omega -\alpha c\right)\left(\omega -\alpha c^{-1}\right)}{\omega \left(\omega -c\right)\left(\omega -c^{-1}\right)}\right)}^{2N}R^{U}\left(\omega ,\zeta \right)\frac{\omega^{N}w^{y}\lambda {\left(z\right)}^{x}}{\zeta^{N+1}z^{y+1}\lambda {\left(w\right)}^{x+\varepsilon_{x}}}\left(\begin{array}{cc} \frac{(1+\alpha^{3})z}{\left(1-\alpha \right)(\lambda \left(z\right)-\alpha^{2}-z)} & \frac{1}{1-\alpha } \end{array}\right){\left(\begin{array}{cc} 1 & 1\\ \alpha z & 1 \end{array}\right)}^{\varepsilon_{x}}d\zeta d\omega , \end{array}$$

where $\gamma_{C}$ is a closed curve surrounding *c* and $c^{-1}$ once in the positive direction, such that it does not surround 0. On the other hand, using again (75) and (77), we have

$$\begin{array}{c} \frac{w^{y}\lambda {\left(z\right)}^{x}}{z^{y}\lambda {\left(w\right)}^{x}}=\frac{{\left(\omega -c\right)}^{y}{\left(\omega -c^{-1}\right)}^{y}}{{\left(\zeta -c\right)}^{y}{\left(\zeta -c^{-1}\right)}^{y}}\frac{{\left(\zeta -\alpha c\right)}^{x}{\left(\zeta -\alpha c^{-1}\right)}^{x}}{{\left(\omega -\alpha c\right)}^{x}{\left(\omega -\alpha c^{-1}\right)}^{x}}\frac{\omega^{x-y}}{\zeta^{x-y}}. \end{array}$$

By using the definition (24) of *W*, we can rewrite (95) as

(96) $$\begin{array}{cc} & {\left[K(2x+\varepsilon_{x},2y+j,2x+\varepsilon_{x},2y+i)\right]}_{i,j=0}^{1}=\frac{1}{{\left(2\pi i\right)}^{2}}\int_{\gamma_{C}}\int_{\gamma_{C}}H_{K}\left(\omega ,\zeta ;\varepsilon_{x}\right)\\ & W\left(\omega \right)R^{U}\left(\omega ,\zeta \right)\frac{\omega^{N+x-y}}{\zeta^{N+x-y}}\frac{{\left(\omega -c\right)}^{y}{\left(\omega -c^{-1}\right)}^{y}}{{\left(\zeta -c\right)}^{y}{\left(\zeta -c^{-1}\right)}^{y}}\frac{{\left(\zeta -\alpha c\right)}^{x}{\left(\zeta -\alpha c^{-1}\right)}^{x}}{{\left(\omega -\alpha c\right)}^{x}{\left(\omega -\alpha c^{-1}\right)}^{x}}d\zeta d\omega , \end{array}$$

where $H_{K}\left(\omega ,\zeta ;\varepsilon_{x}\right)$ is defined for ω,ζ∈C and $\varepsilon_{x}\in \left\{0,1\right\}$ by

(97) $$\begin{array}{cc} H_{K}\left(\omega ,\zeta ;\varepsilon_{x}\right)= & \;\frac{1}{\zeta z\lambda {\left(w\right)}^{\varepsilon_{x}}}{\left(\begin{array}{cc} \alpha^{2} & \alpha \\ w & 1 \end{array}\right)}^{\varepsilon_{x}}\left(\begin{array}{c} 1\\ \frac{1-\alpha +\alpha^{2}}{1-\alpha }\frac{\omega -c}{\omega } \end{array}\right)\left(\begin{array}{cc} \frac{\alpha }{c{\left(1-\alpha \right)}^{2}}\left(\zeta -c^{-1}\right) & \frac{1}{1-\alpha } \end{array}\right){\left(\begin{array}{cc} 1 & 1\\ \alpha z & 1 \end{array}\right)}^{\varepsilon_{x}}. \end{array}$$

Using the identities

$$\begin{array}{cccccc} & z=\frac{\alpha \left(\zeta -c\right)\left(\zeta -c^{-1}\right)}{c{\left(1-\alpha \right)}^{2}\zeta }, & & w=\frac{\alpha \left(\omega -c\right)\left(\omega -c^{-1}\right)}{c{\left(1-\alpha \right)}^{2}\omega }, & & \lambda \left(w\right)=\frac{\alpha \left(\omega -\alpha c\right)\left(\omega -\alpha c^{-1}\right)}{c{\left(1-\alpha \right)}^{2}\omega }, \end{array}$$

it is a simple computation to verify that (97) can be rewritten as (30) and (31). This finishes the proof.

## 7 LOZENGE PROBABILITIES

This section is about the lozenge probabilities $P_{j}\left(x,y\right)$, j=1,2,3, defined in (37). In Section 7.1, we use Theorem 1 to find double contour formulas for $P_{j}\left(x,y\right)$, j=1,2,3, in terms of $R^{U}$. In the rest of this section, we follow Ref. 21, section 7, and use the symmetries in our model to restrict our attention to the lower left part $\eta \leq \frac{\xi }{2}\leq 0$ of the liquid region for the proof of Theorem 2.

### 7.1 Double contour formulas

Formula (29) for the kernel can be rewritten as

(98) $$\begin{array}{c} {\left[K(2x+\varepsilon_{x},2y+j,2x+\varepsilon_{x},2y+i)\right]}_{i,j=0}^{1}\\ =\frac{1}{{\left(2\pi i\right)}^{2}}\int_{\gamma_{C}}\int_{\gamma_{C}}H_{K}\left(\omega ,\zeta ;\varepsilon_{x}\right)W\left(\omega \right)R^{U}\left(\omega ,\zeta \right)\frac{\omega^{N}}{\zeta^{N}}q{\left(\omega ,\zeta \right)}^{y}\tilde{q}{\left(\omega ,\zeta \right)}^{x}d\zeta d\omega , \end{array}$$

where *q* and $\tilde{q}$ are given by

(99) $$\begin{array}{cc} & q\left(\omega ,\zeta \right):=\frac{\zeta \left(\omega -c\right)\left(\omega -c^{-1}\right)}{\omega \left(\zeta -c\right)\left(\zeta -c^{-1}\right)},\;\tilde{q}\left(\omega ,\zeta \right)=\frac{\omega \left(\zeta -\alpha c\right)\left(\zeta -\alpha c^{-1}\right)}{\zeta \left(\omega -\alpha c\right)\left(\omega -\alpha c^{-1}\right)}. \end{array}$$

The double contour formulas for $P_{j}\left(x,y\right)$, j=1,2,3, are obtained via a series of lemmas. Let us first recall that the paths $p_{j}:\left\{0,1,\ldots ,4N\right\}\to Z+\frac{1}{2}$, j=0,…,2N−1 are defined in (5) via (3). We define the height function $h:\left\{0,1,\ldots ,4N\right\}\times Z\to N_{\geq 0}$ by

(100) $$\begin{array}{c} h\left(x,y\right)=\#\{j|p_{j}\left(x\right)<y\}. \end{array}$$

Lemma 4 below is identical to Ref. 21, lemma 7.2, and allows to recover the lozenges from the height function.

Lemma 4 For x∈{0,1,…,4N} and y∈Z, the following identities hold:
(101)
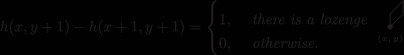

(102)
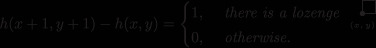

(103)
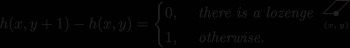

This is an immediate consequence of (3) and (100).▪

The next lemma establishes a double integral formula for the expectation value of the height function.

Lemma 5 For x∈{1,2,…,2N−1}, y∈Z, and $\varepsilon_{x},\varepsilon_{y}\in \left\{0,1\right\}$, we have

(104) $$\begin{array}{c} E\left[h\left(2x+\varepsilon_{x},2y+\varepsilon_{y}\right)\right]=\frac{1}{{\left(2\pi i\right)}^{2}}\int_{{\tilde{\gamma }}_{C}}d\zeta \int_{\gamma_{C}}\frac{d\omega }{q(\omega ,\zeta )-1}R^{U}\left(\omega ,\zeta \right)W\left(\omega \right)\\ \times \frac{\omega^{N}}{\zeta^{N}}q{\left(\omega ,\zeta \right)}^{y}\tilde{q}{\left(\omega ,\zeta \right)}^{x}\left(q{\left(\omega ,\zeta \right)}^{\varepsilon_{y}}H_{K}{\left(\omega ,\zeta ;\varepsilon_{x}\right)}_{11}+H_{K}{\left(\omega ,\zeta ;\varepsilon_{x}\right)}_{22}\right), \end{array}$$

where $\gamma_{C}$ is a closed curve surrounding both *c* and $c^{-1}$, but not surrounding 0, and ${\tilde{\gamma }}_{C}$ is a deformation of $\gamma_{C}$ lying in the bounded region delimited by $\gamma_{C}$, such that |q(ζ,ω)|>1 whenever $\zeta \in {\tilde{\gamma }}_{C}$ and $\omega \in \gamma_{C}$.

Let $X(\tilde{x},\tilde{y})$ be the random variable that counts the number of paths going through the point $\left(\tilde{x},\tilde{y}\right)$, $\tilde{x},\tilde{y}\in \left\{0,1,\ldots ,4N\right\}$. Because $X\left(\tilde{x},\tilde{y}\right)\in \left\{0,1\right\}$, we have $P\left(X\left(\tilde{x},\tilde{y}\right)=1\right)=E\left(X\left(\tilde{x},\tilde{y}\right)\right)$. Also, note that the identity (18) with k=1 is equivalent to $P\left(X\left(\tilde{x},\tilde{y}\right)=1\right)=K\left(\tilde{x},\tilde{y},\tilde{x},\tilde{y}\right)$. Thus, by definition (100) of *h*, we get

(105) $$\begin{array}{cc} E[h\left(2x+\varepsilon_{x},2y\right)] & =\sum_{k<y}\left[K\left(2x+\varepsilon_{x},2k,2x+\varepsilon_{x},2k\right)+K\left(2x+\varepsilon_{x},2k+1,2x+\varepsilon_{x},2k+1\right)\right]\\ & =\sum_{k<y}\text{Tr}{\left[K(2x+\varepsilon_{x},2k+j,2x+\varepsilon_{x},2k+i)\right]}_{i,j=0}^{1}. \end{array}$$

Let us define ${\tilde{\gamma }}_{C}:=C\left(c,r\right)\cup C\left(c^{-1},r\right)$, where C(a,r) denotes a circle oriented positively centered at *a* of radius *r*. We see from (99) that |q(ω,ζ)|→+∞ as ζ tends to *c* or $c^{-1}$. Thus, by choosing *r* sufficiently small, we can make sure that ${\tilde{\gamma }}_{C}$ lies in the interior region of $\gamma_{C}$, and that

$$\begin{array}{c} \left|q(\omega ,\zeta )\right|>1+\varepsilon ,\;\text{for}\;\text{all}\;\zeta \in {\tilde{\gamma }}_{C}\;\text{and}\;\omega \in \gamma_{C}, \end{array}$$

for a certain ε>0. Therefore, uniformly for $\zeta \in {\tilde{\gamma }}_{C}$ and $\omega \in \gamma_{C}$, we have

(106) $$\begin{array}{cc} & \sum_{k<y}q{\left(\omega ,\zeta \right)}^{k}=\frac{q{\left(\omega ,\zeta \right)}^{y}}{q(\omega ,\zeta )-1}. \end{array}$$

The statement (104) with $\varepsilon_{y}=0$ follows after combining (98), (105), and (106). Then, (104) with $\varepsilon_{y}=1$ follows from

$$\begin{array}{c} E\left[h\left(2x+\varepsilon_{x},2y+1\right)\right]=E\left[h\left(2x+\varepsilon_{x},2y\right)\right]+K\left(2x+\varepsilon_{x},2y,2x+\varepsilon_{x},2y\right). \end{array}$$

▪

The double contour formulas for $P_{j}$, j=1,2,3 are stated in the following proposition.

Proposition 8 For x∈{1,2,…,2N−1} and y∈Z, we have

(107) $$\begin{array}{cc} & P_{1}\left(x,y\right)=\frac{1}{{\left(2\pi i\right)}^{2}}\int_{\gamma_{C}}\int_{\gamma_{C}}H_{1}\left(\omega ,\zeta \right)W\left(\omega \right)R^{U}\left(\omega ,\zeta \right)\frac{\omega^{N}}{\zeta^{N}}q{\left(\omega ,\zeta \right)}^{y}\tilde{q}{\left(\omega ,\zeta \right)}^{x}d\zeta d\omega , \end{array}$$

(108) $$\begin{array}{cc} & P_{2}\left(x,y\right)=\frac{1}{{\left(2\pi i\right)}^{2}}\int_{\gamma_{C}}\int_{\gamma_{C}}H_{2}\left(\omega ,\zeta \right)W\left(\omega \right)R^{U}\left(\omega ,\zeta \right)\frac{\omega^{N}}{\zeta^{N}}q{\left(\omega ,\zeta \right)}^{y}\tilde{q}{\left(\omega ,\zeta \right)}^{x}d\zeta d\omega , \end{array}$$

(109) $$\begin{array}{cc} & P_{3}\left(x,y\right)=\left(\begin{array}{cc} 1 & \;1\\ 1 & \;1 \end{array}\right)-\frac{1}{{\left(2\pi i\right)}^{2}}\int_{\gamma_{C}}\int_{\gamma_{C}}H_{3}\left(\omega ,\zeta \right)W\left(\omega \right)R^{U}\left(\omega ,\zeta \right)\frac{\omega^{N}}{\zeta^{N}}q{\left(\omega ,\zeta \right)}^{y}\tilde{q}{\left(\omega ,\zeta \right)}^{x}d\zeta d\omega , \end{array}$$

where *H*_1_, *H*_2_, and *H*_3_ are given by

(110) $$\begin{array}{cc} & H_{1}\left(\omega ,\zeta \right)=\left(\begin{array}{cc} \frac{\alpha c\left(\omega -c\right)\left(\omega -c^{-1}\right)}{\left(\zeta -c\right)\left(\zeta -c^{-1}\right)\omega \left(\omega -\alpha c\right)} & \frac{\left(\zeta -\alpha c\right)\left(\omega -c\right)\left(\omega -c^{-1}\right)}{\left(\zeta -c\right)\left(\zeta -c^{-1}\right)\left(\omega -\alpha c\right)\left(\omega -\alpha c^{-1}\right)}\\ \frac{\omega -c}{\left(\zeta -c)(\omega -\alpha c\right)} & \frac{\alpha (\zeta -\alpha c)(\omega -c)}{c\zeta \left(\zeta -c\right)\left(\omega -\alpha c\right)\left(\omega -\alpha c^{-1}\right)} \end{array}\right), \end{array}$$

(111) $$\begin{array}{cc} & H_{2}\left(\omega ,\zeta \right)=\left(\begin{array}{cc} \frac{c(1-\alpha )(\omega -c)}{\alpha \left(\zeta -c\right)\left(\zeta -c^{-1}\right)\left(\omega -\alpha c\right)} & \frac{\left(1-\alpha )(\zeta -\alpha c)(\omega -c\right)}{c\left(\zeta -c\right)\left(\zeta -c^{-1}\right)\left(\omega -\alpha c\right)\left(\omega -\alpha c^{-1}\right)}\\ \frac{(1-\alpha )c}{\left(\zeta -c)(\omega -\alpha c\right)} & \frac{\alpha c(1-\alpha )(\zeta -\alpha c)\omega }{\zeta \left(\zeta -c\right)\left(\omega -\alpha c\right)\left(\omega -\alpha c^{-1}\right)} \end{array}\right), \end{array}$$

(112) $$\begin{array}{cc} & H_{3}\left(\omega ,\zeta \right)=\left(\begin{array}{cc} \frac{\omega -c}{c\omega \left(\zeta -c\right)\left(\zeta -c^{-1}\right)} & \frac{\left(\zeta -\alpha c)(\omega -c\right)}{\left(\zeta -c\right)\left(\zeta -c^{-1}\right)\left(\omega -\alpha c\right)}\\ \frac{1}{\zeta -c} & \frac{c(\zeta -\alpha c)}{\zeta (\zeta -c)(\omega -\alpha c)} \end{array}\right). \end{array}$$

Recall that $P_{j}$, j=1,2,3 are defined by (36). By (18), for $\varepsilon_{x},\varepsilon_{y}\in \left\{0,1\right\}$, we have

$$\begin{array}{cc} & P_{3}\left(2x+\varepsilon_{x},2y+\varepsilon_{y}\right)=1-K\left(2x+\varepsilon_{x},2y+\varepsilon_{y},2x+\varepsilon_{x},2y+\varepsilon_{y}\right). \end{array}$$

Noting that

$$\begin{array}{c} H_{3}\left(\omega ,\zeta \right)=\left(\begin{array}{cc} H_{K}{\left(\omega ,\zeta ;0\right)}_{22} & H_{K}{\left(\omega ,\zeta ;1\right)}_{22}\\ H_{K}{\left(\omega ,\zeta ;0\right)}_{11} & H_{K}{\left(\omega ,\zeta ;1\right)}_{11} \end{array}\right)=\left(\begin{array}{cc} \frac{\omega -c}{c\omega \left(\zeta -c\right)\left(\zeta -c^{-1}\right)} & \frac{\left(\zeta -\alpha c)(\omega -c\right)}{\left(\zeta -c\right)\left(\zeta -c^{-1}\right)\left(\omega -\alpha c\right)}\\ \frac{1}{\zeta -c} & \frac{c(\zeta -\alpha c)}{\zeta (\zeta -c)(\omega -\alpha c)} \end{array}\right), \end{array}$$

formula (109) follows by combining (37) with (98). The proof of (107) and (108) requires more work and relies on Lemmas 4 and 5. First, we note the following direct consequences of (104):

(113) $$\begin{array}{cc} & E\left[h\left(2x+\varepsilon_{x},2y+1+\varepsilon_{y}\right)\right]=\frac{1}{{\left(2\pi i\right)}^{2}}\int_{{\tilde{\gamma }}_{C}}d\zeta \int_{\gamma_{C}}\frac{d\omega }{q(\omega ,\zeta )-1}R^{U}\left(\omega ,\zeta \right)W\left(\omega \right)\\ & \;\times \frac{\omega^{N}}{\zeta^{N}}q{\left(\omega ,\zeta \right)}^{y}\tilde{q}{\left(\omega ,\zeta \right)}^{x}\left(q\left(\omega ,\zeta \right)H_{K}{\left(\omega ,\zeta ;\varepsilon_{x}\right)}_{11}+q{\left(\omega ,\zeta \right)}^{\varepsilon_{y}}H_{K}{\left(\omega ,\zeta ;\varepsilon_{x}\right)}_{22}\right), \end{array}$$

(114) $$\begin{array}{cc} & E\left[h\left(2x+1+\varepsilon_{x},2y+1+\varepsilon_{y}\right)\right]=\frac{1}{{\left(2\pi i\right)}^{2}}\int_{{\tilde{\gamma }}_{C}}d\zeta \int_{\gamma_{C}}\frac{d\omega }{q(\omega ,\zeta )-1}R^{U}\left(\omega ,\zeta \right)W\left(\omega \right)\\ & \;\times \frac{\omega^{N}}{\zeta^{N}}q{\left(\omega ,\zeta \right)}^{y}\tilde{q}{\left(\omega ,\zeta \right)}^{x+\varepsilon_{x}}\left(q\left(\omega ,\zeta \right)H_{K}{\left(\omega ,\zeta ;1-\varepsilon_{x}\right)}_{11}+q{\left(\omega ,\zeta \right)}^{\varepsilon_{y}}H_{K}{\left(\omega ,\zeta ;1-\varepsilon_{x}\right)}_{22}\right). \end{array}$$

Using (101), (113), and (114), we get

(115) $$\begin{array}{cc} & P_{1}\left(2x+\varepsilon_{x},2y+\varepsilon_{y}\right)=E\left[h\left(2x+\varepsilon_{x},2y+1+\varepsilon_{y}\right)\right]-E\left[h\left(2x+1+\varepsilon_{x},2y+1+\varepsilon_{y}\right)\right]\\ & =\frac{1}{{\left(2\pi i\right)}^{2}}\int_{{\tilde{\gamma }}_{C}}d\zeta \int_{\gamma_{C}}\frac{d\omega }{q(\omega ,\zeta )-1}R^{U}\left(\omega ,\zeta \right)W\left(\omega \right)\frac{\omega^{N}}{\zeta^{N}}q{\left(\omega ,\zeta \right)}^{y}\tilde{q}{\left(\omega ,\zeta \right)}^{x}\\ & \;\times \left(q\left(\omega ,\zeta \right)H_{K}{\left(\omega ,\zeta ;\varepsilon_{x}\right)}_{11}-q\left(\omega ,\zeta \right)\tilde{q}{\left(\omega ,\zeta \right)}^{\varepsilon_{x}}H_{K}{\left(\omega ,\zeta ;1-\varepsilon_{x}\right)}_{11}\right.\\ & \;\left.+\;q\;{\left(\omega ,\zeta \right)}^{\varepsilon_{y}}H_{K}{\left(\omega ,\zeta ;\varepsilon_{x}\right)}_{22}-q{\left(\omega ,\zeta \right)}^{\varepsilon_{y}}\tilde{q}{\left(\omega ,\zeta \right)}^{\varepsilon_{x}}H_{K}{\left(\omega ,\zeta ;1-\varepsilon_{x}\right)}_{22}\right). \end{array}$$

It is a direct computation to verify that the integrand has no pole at ζ=ω for any $\varepsilon_{x},\varepsilon_{y}\in \left\{0,1\right\}$, so that ${\tilde{\gamma }}_{C}$ can be deformed back to $\gamma_{C}$. We obtain (107) after writing (115) in the matrix form (37). Finally, using (102), (104), and (114), we get

$$\begin{array}{cc} & P_{2}\left(2x+\varepsilon_{x},2y+\varepsilon_{y}\right)=E\left[h\left(2x+1+\varepsilon_{x},2y+1+\varepsilon_{y}\right)\right]-E\left[h\left(2x+\varepsilon_{x},2y+\varepsilon_{y}\right)\right]\\ & =\frac{1}{{\left(2\pi i\right)}^{2}}\int_{{\tilde{\gamma }}_{C}}d\zeta \int_{\gamma_{C}}\frac{d\omega }{q(\omega ,\zeta )-1}R^{U}\left(\omega ,\zeta \right)W\left(\omega \right)\frac{\omega^{N}}{\zeta^{N}}q{\left(\omega ,\zeta \right)}^{y}\tilde{q}{\left(\omega ,\zeta \right)}^{x}\\ & \;\times \left(q\left(\omega ,\zeta \right)\tilde{q}{\left(\omega ,\zeta \right)}^{\varepsilon_{x}}H_{K}{\left(\omega ,\zeta ;1-\varepsilon_{x}\right)}_{11}-q{\left(\omega ,\zeta \right)}^{\varepsilon_{y}}H_{K}{\left(\omega ,\zeta ;\varepsilon_{x}\right)}_{11}\right.\\ & \;\left.+\;q{\left(\omega ,\zeta \right)}^{\varepsilon_{y}}\tilde{q}{\left(\omega ,\zeta \right)}^{\varepsilon_{x}}H_{K}{\left(\omega ,\zeta ;1-\varepsilon_{x}\right)}_{22}-H_{K}{\left(\omega ,\zeta ;\varepsilon_{x}\right)}_{22}\right). \end{array}$$

Another direct computation shows that the integrand has no pole at ζ=ω for any $\varepsilon_{x},\varepsilon_{y}\in \left\{0,1\right\}$, so that ${\tilde{\gamma }}_{C}$ can be deformed back to $\gamma_{C}$. The formula (108) is then obtained by rewriting the above in the matrix form (37).▪

### 7.2 Symmetries

Let H(ω,ζ) be a 2 × 2 meromorphic function in both ζ and ω, whose only possible poles in each variable are at 0, αc, $\alpha c^{-1}$, *c*, and $c^{-1}$. Furthermore, we assume that all the poles of *H* are of order 1 and that H(ω,ζ) is bounded as ζ and/or ω tend to ∞. For x∈{1,2,…,2N−1} and y∈Z, we define

(116) $$\begin{array}{c} I\left(x,y;H\right)=\frac{1}{{\left(2\pi i\right)}^{2}}\int_{\gamma_{C}}\int_{\gamma_{C}}H\left(\omega ,\zeta \right)W\left(\omega \right)R^{U}\left(\omega ,\zeta \right)\frac{\omega^{N}}{\zeta^{N}}q{\left(\omega ,\zeta \right)}^{y}\tilde{q}{\left(\omega ,\zeta \right)}^{x}d\zeta d\omega . \end{array}$$

Because the poles of *H* are of order at most 1, recalling (24), the only poles of the integrand are at 0, *c*, and $c^{-1}$, in both the ζ and ω variables. The following star‐operation will play an important role for a symmetry property of I:

(117) $$\begin{array}{c} \zeta^{*}=c^{-1}+\frac{R_{1}^{2}}{\zeta -c^{-1}}\;\text{where}\;R_{1}=\frac{1-\alpha }{\sqrt{\alpha }}. \end{array}$$

Let γ_1_ be the circle centered at $c^{-1}$ of radius *R*_1_. The star‐operation maps γ_1_ into itself, but reverses the orientation. Furthermore, it satisfies ${\left(\zeta^{*}\right)}^{*}=\zeta$ for all ζ∈C∪{∞}. We start by proving some symmetries for $R^{U}$.

Lemma 6 The reproducing kernel $R^{U}$ satisfies two symmetries.

(a) We have
  (118) $$\begin{array}{c} R^{U}\left(\omega ,\zeta \right)=R^{U}\left(\zeta ,\omega \right),\;\omega ,\zeta \in C. \end{array}$$
(b) We have
  (119) $$R^{U}\left(\omega^{*},\zeta^{*}\right)=\frac{R_{1}^{4N-2}R^{U}\left(\omega ,\zeta \right)}{{\left(\omega -c^{-1}\right)}^{2N-1}{\left(\zeta -c^{-1}\right)}^{2N-1}},\;\omega ,\zeta \in C\setminus \left\{c^{-1}\right\}.$$

Because detU≡1, it follows from (28) that

(120) $$\begin{array}{c} R^{U}\left(\omega ,\zeta \right)=\frac{U_{11}\left(\omega \right)U_{21}\left(\zeta \right)-U_{11}\left(\zeta \right)U_{21}\left(\omega \right)}{\zeta -\omega }, \end{array}$$

from which we deduce (118). Now we prove (b). Note that the first column of *U* only contains polynomials, which are independent of the choice of the contour $\gamma_{C}$ that appears in the formulation of the RH problem for *U*. Therefore, $R^{U}$ is independent of the choice of $\gamma_{C}$ as well by (120). Because γ_1_ encloses both *c* and $c^{-1}$, and does not enclose 0, γ_1_ is a valid choice of contour. We use the freedom we have in the choice of $\gamma_{C}$ by letting *U* be the solution to the RH problem for *U* associated to the contour γ_1_. We can verify by direct computations that

(121) $$\begin{array}{c} W\left(\zeta^{*}\right)=\frac{{\left(\omega -c^{-1}\right)}^{4N}}{R_{1}^{4N}}W\left(\zeta \right), \end{array}$$

so that

$$\hat{U}\left(\zeta \right):=\left(\begin{array}{cc} R_{1}^{2N} & 0\\ 0 & -R_{1}^{-2N} \end{array}\right)U{\left(\frac{1}{c}\right)}^{-1}U\left(\zeta^{*}\right)\left(\begin{array}{cc} \frac{{\left(\zeta -c^{-1}\right)}^{2N}}{R_{1}^{2N}} & 0\\ 0 & -\frac{R_{1}^{2N}}{{\left(\zeta -c^{-1}\right)}^{2N}} \end{array}\right)$$

also satisfies the conditions of the RH problem for *U*. By uniqueness of the solution of this RH problem, we infer that $U\left(\zeta \right)=\hat{U}\left(\zeta \right)$. After replacing (ω,ζ) by $\left(\omega^{*},\zeta^{*}\right)$ in (28) and using the relations $\hat{U}\left(\zeta \right)=U\left(\zeta \right)$ and $\frac{\zeta -\omega }{\zeta^{*}-\omega^{*}}=-\frac{\left(\zeta -c^{-1}\right)\left(\omega -c^{-1}\right)}{R_{1}^{2}}$, we obtain (119).▪

Proposition 9 The double integral I(x,y;H) satisfies two symmetries.

(a) The following (x,y)↦(2N−x,2N−y) symmetry holds:
  (122) $$\begin{array}{cc} I(2N-x,2N-y;H) & =I(x,y;\hat{H}), \end{array}$$
  with
  (123) $$\hat{H}\left(\omega ,\zeta \right)=H\left(\zeta ,\omega \right).$$
(b) The following (x,y)↦(x,N+x−y) symmetry holds:
  (124) $$\begin{array}{cc} I(x,N+x-y;H) & =I(x,y;\tilde{H}) \end{array}$$
  with
  (125) $$\tilde{H}\left(\omega ,\zeta \right)=\frac{R_{1}^{2}H\left(\omega^{*},\zeta^{*}\right)}{\left(\omega -c^{-1}\right)\left(\zeta -c^{-1}\right)}.$$

(a) From (99), we verify that

(126) $$\begin{array}{c} \frac{\omega^{N}}{\zeta^{N}}q{\left(\omega ,\zeta \right)}^{2N-y}\tilde{q}{\left(\omega ,\zeta \right)}^{2N-x}=\frac{\zeta^{N}}{\omega^{N}}\frac{W(\zeta )}{W(\omega )}q{\left(\zeta ,\omega \right)}^{y}\tilde{q}{\left(\zeta ,\omega \right)}^{x}. \end{array}$$

Replacing (x,y) in (116) by (2N−x,2N−y), and then using (126), we get

(127) $$\begin{array}{c} I\left(2N-x,2N-y;H\right)=\frac{1}{{\left(2\pi i\right)}^{2}}\int_{\gamma_{C}}\int_{\gamma_{C}}W\left(\zeta \right)R^{U}\left(\omega ,\zeta \right)\frac{\zeta^{N}}{\omega^{N}}q{\left(\zeta ,\omega \right)}^{y}\tilde{q}{\left(\zeta ,\omega \right)}^{x}H\left(\omega ,\zeta \right)d\zeta d\omega . \end{array}$$

Recalling (118), the identity (122) follows after interchanging variables in (127).

(b) Note that γ_1_ encloses both *c* and $c^{-1}$, and does not enclose 0, so we can (and do) deform $\gamma_{C}$ to γ_1_ in (116). We first replace (x,y) by (x,N+x−y) in (116), and then perform the change of variables $\zeta \mapsto \zeta^{*}$ and $\omega \mapsto \omega^{*}$. This gives

$$\begin{array}{c} I\left(x,N+x-y\right)=\frac{1}{{\left(2\pi i\right)}^{2}}\int_{\gamma_{1}}\int_{\gamma_{1}}W\left(\omega^{*}\right)R^{U}\left(\omega^{*},\zeta^{*}\right)\frac{\omega^{*\;N}}{\zeta^{*\;N}}q{\left(\omega^{*},\zeta^{*}\right)}^{N+x-y}\tilde{q}{\left(\omega^{*},\zeta \right)}^{x}H\left(\omega^{*},\zeta^{*}\right)d\zeta^{*}d\omega^{*}. \end{array}$$

It is a long but direct computation to verify that

$$\begin{array}{cccc} & q{\left(\omega^{*},\zeta^{*}\right)}^{N+x-y}=q{\left(\omega ,\zeta \right)}^{y-x-N}, & & \tilde{q}{\left(\omega^{*},\zeta^{*}\right)}^{x}=q{\left(\omega ,\zeta \right)}^{x}\tilde{q}{\left(\omega ,\zeta \right)}^{x},\\ & \frac{\omega^{*\;N}}{\zeta^{*\;N}}=\frac{{\left(\omega -c\right)}^{N}{\left(\zeta -c^{-1}\right)}^{N}}{{\left(\omega -c^{-1}\right)}^{N}{\left(\zeta -c\right)}^{N}}, & & H\left(\omega^{*},\zeta^{*}\right)d\zeta^{*}d\omega^{*}=\frac{H\left(\omega^{*},\zeta^{*}\right)R_{1}^{4}d\zeta d\omega }{{\left(\zeta -c^{-1}\right)}^{2}{\left(\omega -c^{-1}\right)}^{2}}. \end{array}$$

Recalling also (119) and (121), (124) follows by deforming back γ_1_ to the original contour $\gamma_{C}$ (in each variable).▪

We recall that s(ξ,η;α) is defined for $\left(\xi ,\eta \right)\in L_{\alpha }$ as the unique solution of (41) lying in the upper half‐plane, and that Q is defined by (32). These quantities will appear naturally in the analysis of the next sections. For now, we simply note the following symmetries for s(ξ,η;α).

Proposition 10 Let $\left(\xi ,\eta \right)\in L_{\alpha }$. Then also $\left(-\xi ,-\eta \right)\in L_{\alpha }$, $\left(\xi ,\xi -\eta \right)\in L_{\alpha }$ and

(128) $$\begin{array}{cc} s(-\xi ,-\eta ;\alpha ) & =s(\xi ,\eta ;\alpha ) \end{array}$$

(129) $$\begin{array}{cc} s(\xi ,\xi -\eta ;\alpha ) & ={\left(\bar{s(\xi ,\eta ;\alpha )}\right)}^{*}, \end{array}$$

where * denotes the star‐operation defined in (117).

The symmetry (128) is part of Proposition 4 and has already been proved in Section 4. It remains to prove (129). We define the function *f* as follows:

$$\begin{array}{c} f\left(\zeta ;\xi ,\eta \right)=-\frac{\xi -\eta }{2}\frac{1}{\zeta }+\frac{\xi }{2}\left(\frac{1}{\zeta -\alpha c}+\frac{1}{\zeta -\alpha c^{-1}}\right)-\frac{\eta }{2}\left(\frac{1}{\zeta -c}+\frac{1}{\zeta -c^{-1}}\right), \end{array}$$

so that (41) can be rewritten as

(130) $$\begin{array}{c} f{\left(\zeta ;\xi ,\eta \right)}^{2}=Q\left(\zeta \right). \end{array}$$

Note that both *f* and Q depend on α, even though this is not indicated in the notation. It is a long but direct computation to verify that

(131) $$\begin{array}{c} \frac{R_{1}^{4}}{{\left(\zeta -c^{-1}\right)}^{4}}Q\left(\zeta^{*}\right)=Q\left(\zeta \right),\;\;\text{and}\;\;-\frac{R_{1}^{2}}{{\left(\zeta -c^{-1}\right)}^{2}}f\left(\zeta^{*};\xi ,\eta \right)=f\left(\zeta ;\xi ,\xi -\eta \right). \end{array}$$

By definition of s(ξ,η;α), we have $f{\left(s\left(\xi ,\eta ;\alpha \right);\xi ,\eta \right)}^{2}=Q\left(s\left(\xi ,\eta ;\alpha \right)\right)$, so the symmetry (131) implies that

(132) $$\begin{array}{c} f{\left(s{\left(\xi ,\eta ;\alpha \right)}^{*};\xi ,\xi -\eta \right)}^{2}=Q\left(s{\left(\xi ,\eta ;\alpha \right)}^{*}\right). \end{array}$$

Because the star operation maps the upper half‐plane to the lower half‐plane, $s{\left(\xi ,\eta ;\alpha \right)}^{*}$ lies in the lower half‐plane. Therefore, applying the conjugate operation in (132), and noting that $\bar{f(\zeta )}=f\left(\bar{\zeta }\right)$ and $\bar{Q(\zeta )}=Q\left(\bar{\zeta }\right)$, we infer that $\left(\xi ,\xi -\eta \right)\in L_{\alpha }$ if and only if $\left(\xi ,\eta \right)\in L_{\alpha }$, and that (129) holds.▪

### 7.3 Preliminaries to the asymptotic analysis

Proposition 11 Let $\{{\left(x_{N},y_{N}\right\}}_{N\geq 1}$ be a sequence satisfying (38) with $\left(\xi ,\eta \right)\in L_{\alpha }$, such that $\eta \leq \frac{\xi }{2}\leq 0$. If (ξ,η) lies on the boundary of $\left\{\eta \leq \frac{\xi }{2}\leq 0\right\}$, then we assume furthermore that $\frac{y_{N}}{N}-1\leq \frac{1}{2}\left(\frac{x_{N}}{N}-1\right)\leq 0$ for all sufficiently large *N*. Let (ω,ζ)↦H(ω,ζ) be a 2 × 2 meromorphic function in both ζ and ω, whose only possible poles in each variable are at 0, αc, $\alpha c^{-1}$, *c*, and $c^{-1}$. Furthermore, we assume that all the poles of *H* are of order 1 and that H(ω,ζ) is bounded as ζ and/or ω tend to ∞. Then, $I(x_{N},y_{N};H)$ defined in (116) has the limit

(133) $$\lim_{N\to \infty }I\left(x_{N},y_{N};H\right)=\frac{1}{2\pi i}\int_{\bar{s}}^{s}H\left(\zeta ,\zeta \right)d\zeta ,$$

where s=s(ξ,η;α), and the integration path is from $\bar{s}$ to *s* and lies in $C\setminus (-\infty ,c^{-1}]$.

The proof of Proposition 11 will be given in Section 11, after considerable preparations have been carried out in Sections 8, 10.

Proposition 11 only covers the lower left quadrant $\eta \leq \frac{\xi }{2}\leq 0$ of the liquid region. The next lemma shows that this is sufficient.

Lemma 7 Assume Proposition 11 holds true. Then, the statement of Proposition 11 still holds without the assumption that $\eta \leq \frac{\xi }{2}\leq 0$, and without the assumption that $\frac{y_{N}}{N}-1\leq \frac{1}{2}\left(\frac{x_{N}}{N}-1\right)\leq 0$ for all sufficiently large *N*. That is, it holds for all $\left(\xi ,\eta \right)\in L_{\alpha }$.

If ${\left\{\left(x_{N},y_{N}\right)\right\}}_{N\geq 1}$ is a sequence satisfying (38) with $\left(\xi ,\eta \right)\in L_{\alpha }\cap \left\{\eta >\frac{\xi }{2}>0\right\}$, then ${\left\{\left(2N-x_{N},2N-y_{N}\right)\right\}}_{N\geq 1}$ satisfies (38) with (−ξ,−η) lying in the lower left quadrant of $L_{\alpha }$. Therefore, Proposition 11 applies to the sequence ${\left\{\left(2N-x_{N},2N-y_{N}\right)\right\}}_{N\geq 1}$, and we rely on the symmetries (122) and (128) to conclude

(134) $$\begin{array}{c} \lim_{N\to \infty }I\left(x_{N},y_{N};H\right)=\lim_{N\to \infty }I\left(2N-x_{N},2N-y_{N};\hat{H}\right)\\ =\frac{1}{2\pi i}\int_{\bar{s(-\xi ,-\eta ;\alpha )}}^{s(-\xi ,-\eta ;\alpha )}\hat{H}\left(\zeta ,\zeta \right)d\zeta =\frac{1}{2\pi i}\int_{\bar{s(\xi ,\eta ;\alpha )}}^{s(\xi ,\eta ;\alpha )}H\left(\zeta ,\zeta \right)d\zeta , \end{array}$$

where we have also used (123) for the last equality. Now, if ${\left\{\left(x_{N},y_{N}\right)\right\}}_{N\geq 1}$ is a sequence satisfying (38) with $\left(\xi ,\eta \right)\in L_{\alpha }\cap \left\{\eta >\frac{\xi }{2}<0\right\}$, then ${\left\{\left(x_{N},N+x_{N}-y_{N}\right)\right\}}_{N\geq 1}$ satisfies (38) with (ξ,ξ−η) lying in the lower left quadrant of $L_{\alpha }$, so that Proposition 11 applies. Using the symmetries (124) and (129), we arrive at

(135) $$\begin{array}{ccc} \lim_{N\to \infty }I\left(x_{N},y_{N};H\right) & = & \lim_{N\to \infty }I\left(x_{N},N+x_{N}-y_{N};\tilde{H}\right)=\frac{1}{2\pi i}\int_{\bar{s(\xi ,\xi -\eta ;\alpha )}}^{s(\xi ,\xi -\eta ;\alpha )}\tilde{H}\left(\zeta ,\zeta \right)d\zeta \\ & = & \frac{1}{2\pi i}\int_{s{\left(\xi ,\eta ;\alpha \right)}^{*}}^{{\bar{s(\xi ,\eta ;\alpha )}}^{*}}\frac{R_{1}^{2}H\left(\zeta^{*},\zeta^{*}\right)}{{\left(\zeta -c^{-1}\right)}^{2}}d\zeta =\frac{1}{2\pi i}\int_{\bar{s(\xi ,\eta ;\alpha )}}^{s(\xi ,\eta ;\alpha )}H\left(\zeta ,\zeta \right)d\zeta , \end{array}$$

where, for the last equality, we have applied the change of variables $\zeta \to \zeta^{*}$ stated in (117). The claim for the last quadrant $\left(\xi ,\eta \right)\in L_{\alpha }\cap \left\{\eta <\frac{\xi }{2}>0\right\}$ follows by combining (134) with (135). Finally, if ${\left\{\left(x_{N},y_{N}\right)\right\}}_{N\geq 1}$ satisfies (38) with $\eta =\frac{\xi }{2}$ and/or ξ=0, then we define a new sequence ${\left\{\left({\tilde{x}}_{N},{\tilde{y}}_{N}\right)\right\}}_{N\geq 1}$ as follows. For each *N*, $\left({\tilde{x}}_{N},{\tilde{y}}_{N}\right)$ is equal to

(136) $$\begin{array}{c} \left(x_{N},y_{N}\right),\;\left(2N-x_{N},2N-y_{N}\right),\;\left(x_{N},N+x_{N}-y_{N}\right)\;\;\text{or}\;\;\left(2N-x_{N},N-x_{N}+y_{N}\right), \end{array}$$

in such a way that $\frac{{\tilde{y}}_{N}}{N}-1\leq \frac{1}{2}\left(\frac{{\tilde{x}}_{N}}{N}-1\right)\leq 0$. There are four natural subsequences of ${\left\{\left({\tilde{x}}_{N},{\tilde{y}}_{N}\right)\right\}}_{N\geq 1}$, corresponding to the four sets of indices

$$\begin{array}{cccc} & A_{1}=\left\{N:\left({\tilde{x}}_{N},{\tilde{y}}_{N}\right)=\left(x_{N},y_{N}\right)\right\}, & & A_{2}=\left\{N:\left({\tilde{x}}_{N},{\tilde{y}}_{N}\right)=\left(2N-x_{N},2N-y_{N}\right)\right\},\\ & A_{3}=\left\{N:\left({\tilde{x}}_{N},{\tilde{y}}_{N}\right)=\left(x_{N},N+x_{N}-y_{N}\right)\right\}, & & A_{4}=\left\{N:\left({\tilde{x}}_{N},{\tilde{y}}_{N}\right)=\left(2N-x_{N},N-x_{N}+y_{N}\right)\right\}. \end{array}$$

If any of the four subsequences ${\left\{\left({\tilde{x}}_{N},{\tilde{y}}_{N}\right)\right\}}_{N\geq 1,N\in A_{j}}$, j=1,2,3,4 contains infinitely many elements, Proposition 11 applies and by (133), (134), and (135), we have

(137) $$\begin{array}{c} \lim_{\begin{array}{c} N\to \infty \\ N\in A_{j} \end{array}}I\left(x_{N},y_{N};H\right)=\lim_{\begin{array}{c} N\to \infty \\ N\in A_{j} \end{array}}I\left({\tilde{x}}_{N},{\tilde{y}}_{N};{\tilde{H}}_{j}\right)=\frac{1}{2\pi i}\int_{\bar{s(\xi ,\eta ;\alpha )}}^{s(\xi ,\eta ;\alpha )}H\left(\zeta ,\zeta \right)d\zeta , \end{array}$$

where $H_{j}\left(\omega ,\zeta \right)$, j=1,2,3,4 are equal to $H\left(\omega ,\zeta \right),\hat{H}\left(\omega ,\zeta \right),\tilde{H}\left(\omega ,\zeta \right)$, and $\tilde{H}\left(\zeta ,\omega \right)$, respectively. Because the right‐hand side of (137) is independent of *j*, this shows that

$$\begin{array}{c} \lim_{N\to \infty }I\left(x_{N},y_{N};H\right)=\frac{1}{2\pi i}\int_{\bar{s(\xi ,\eta ;\alpha )}}^{s(\xi ,\eta ;\alpha )}H\left(\zeta ,\zeta \right)d\zeta , \end{array}$$

which finishes the proof.▪

Proposition 12 Proposition 11 implies Theorem 2.

By (107)–(109) and (116), for x∈{1,2,…,2N−1} and y∈Z, we can write

$$\begin{array}{cc} & P_{j}\left(x,y\right)=I\left(x,y;H_{j}\right),\;j=1,2,\\ & P_{3}\left(x,y\right)=\left(\begin{array}{cc} 1 & 1\\ 1 & 1 \end{array}\right)-I\left(x,y;H_{3}\right), \end{array}$$

where the functions $H_{j}$, j=1,2,3, are defined in (110)–(112). Let $\{{\left(x_{N},y_{N}\right\}}_{N\geq 1}$ be a sequence satisfying (38) with $\left(\xi ,\eta \right)\in L_{\alpha }$. By Lemma 7, we do not need to assume $\eta \leq \frac{\xi }{2}\leq 0$ to invoke Proposition 11. Applying Proposition 11 with $H=H_{3}$, we obtain

(138) $$\begin{array}{c} \lim_{N\to \infty }P_{3}\left(x_{N},y_{N}\right)=\left(\begin{array}{cc} 1 & 1\\ 1 & 1 \end{array}\right)-\frac{1}{2\pi i}\int_{\bar{s}}^{s}H_{3}\left(\zeta ,\zeta \right)d\zeta . \end{array}$$

From (112), we see that

(139) $$\begin{array}{c} H_{3}\left(\zeta ,\zeta \right)=\left(\begin{array}{cc} \frac{1}{\zeta -c^{-1}}-\frac{1}{\zeta } & \frac{1}{\zeta -c^{-1}}\\ \frac{1}{\zeta -c} & \frac{1}{\zeta -c}-\frac{1}{\zeta } \end{array}\right), \end{array}$$

and because the path going from $\bar{s}$ to *s* does not cross $\left(-\infty ,c^{-1}\right]$, we get (54) after substituting (139) in (138) and carrying out the integration. Similarly, using (110) and (111), we have

$$\begin{array}{cc} & H_{1}\left(\zeta ,\zeta \right)=\left(\begin{array}{cc} \frac{1}{\zeta -\alpha c}-\frac{1}{\zeta } & \frac{1}{\zeta -\alpha c^{-1}}\\ \frac{1}{\zeta -\alpha c} & \frac{1}{\zeta -\alpha c^{-1}}-\frac{1}{\zeta } \end{array}\right)\;\;\text{and}\;\;H_{2}\left(\zeta ,\zeta \right)=\left(\begin{array}{cc} \frac{1}{\zeta -c^{-1}}-\frac{1}{\zeta -\alpha c} & \frac{1}{\zeta -c^{-1}}-\frac{1}{\zeta -\alpha c^{-1}}\\ \frac{1}{\zeta -c}-\frac{1}{\zeta -\alpha c} & \frac{1}{\zeta -c}-\frac{1}{\zeta -\alpha c^{-1}} \end{array}\right), \end{array}$$

and we obtain (52) and (53) after applying Proposition 11 with $H=H_{1}$ and $H=H_{2}$, respectively.▪

## 8 *g*‐FUNCTION

In Section 9, we will perform a Deift/Zhou^22^ steepest descent analysis on the RH problem for *U*. The first transformation U↦T consists of normalizing the RH problem and requires considerable preparation. This transformation uses a so‐called *g*‐function,^43^ which is of the form

(140) $$\begin{array}{c} g\left(\zeta \right)=\int_{\mathrm{supp}(\mu )}\log\left(\zeta -\xi \right)d\mu \left(\xi \right), \end{array}$$

where μ is a probability measure, dμ is its density, and suppμ is its (bounded and oriented) support. For any choice of μ, the *g*‐function satisfies

$$\begin{array}{cccc} & g\left(\zeta \right)=\log\left(\zeta \right)+O\left(\zeta^{-1}\right), & & \text{as}\;\zeta \to \infty , \end{array}$$

so that $U\left(\zeta \right)e^{-2Ng\left(\zeta \right)\sigma_{3}}$ is normalized at ∞ (with $\sigma_{3}=\text{diag}\left(1,-1\right)$), in the sense that $U\left(\zeta \right)e^{-2Ng\left(\zeta \right)\sigma_{3}}=I_{2}+O\left(\zeta^{-1}\right)$ as ζ→∞. Also, we note that in the definition of *U*, the contour $\gamma_{C}$ can be chosen arbitrarily, as long as it is a closed curve surrounding $c^{-1}$ and *c* once in the positive direction, which does not surround 0. However, to successfully perform an asymptotic analysis on the RH problem for *U*, we need to choose μ and $\gamma_{C}$ appropriately so that the jumps for *T* have “good properties.”

In this section, we find the key ingredients for the Y↦T transformation of Section 9, that is, we find a *g*‐function (built in terms of μ) and a relevant contour $\gamma_{C}$. Let us rewrite *W* as follows:

(141) $$W\left(\zeta \right)={\left(\frac{\left(\zeta -\alpha c\right)\left(\zeta -\alpha c^{-1}\right)}{\zeta \left(\zeta -c\right)\left(\zeta -c^{-1}\right)}\right)}^{2N}=e^{-2NV(\zeta )},$$

where the potential *V* is given by

(142) $$V\left(\zeta \right)=\log\zeta +\log\left(\zeta -c\right)+\log\left(\zeta -c^{-1}\right)-\log\left(\zeta -\alpha c\right)-\log\left(\zeta -\alpha c^{-1}\right)$$

and we take the principal branch for the logarithms. We require *g* and $\gamma_{C}$ to satisfy the following criteria (we define supp(μ) as an oriented open set for convenience):

(a) $\gamma_{C}$ is a closed curve surrounding $c^{-1}$ and *c* once in the positive direction, but not surrounding 0.
(b) $e^{g}$ is analytic in $C\setminus \bar{\mathrm{supp}(\mu )}$, where supp(μ) is an open oriented curve satisfying $\mathrm{supp}\left(\mu \right)\subset \gamma_{C}$.
(c) For ζ∈supp(μ), we let $g_{+}\left(\zeta \right)$ (resp. $g_{-}\left(\zeta \right)$) denote the limit of $g(\zeta^{\prime })$ as $\zeta^{\prime }\to \zeta$ from the left (resp. right) of supp(μ). Here “left” and “right” are with respect to the orientation of supp(μ). The *g*‐function (140) satisfies
  (143) $$\begin{array}{cccc} & g_{+}\left(\zeta \right)+g_{-}\left(\zeta \right)-V\left(\zeta \right)+\ell =0, & & \text{for}\;\zeta \in \mathrm{supp}(\mu ), \end{array}$$
  (144) $$\begin{array}{cccc} & \text{Re}\;\left(g_{+}\left(\zeta \right)+g_{-}\left(\zeta \right)-V\left(\zeta \right)+\ell \right)<0, & & \text{for}\;\zeta \in \gamma_{C}\setminus \bar{\mathrm{supp}(\mu )}, \end{array}$$
  (145) $$\begin{array}{cccc} & \text{Im}\;\left(g_{+}\left(\zeta \right)-\frac{V(\zeta )}{2}+\frac{\ell }{2}\right), & & \text{is}\;\mathrm{decreasing}\;\text{along}\;\mathrm{supp}(\mu ), \end{array}$$
  for some constant ℓ∈C, and where *V* is given by (142).

In approximation theory, the equality (143) together with the inequality (144) are usually referred to as the Euler–Lagrange variational conditions,^45^ and ℓ is the Euler–Lagrange constant. A measure μ satisfying (143) and (144) is called the equilibrium measure^45^ in the external field *V*, because it is the unique minimizer of

$$\begin{array}{c} \tilde{\mu }\mapsto \;\int \int \;\log\frac{1}{\left|s-t\right|}d\tilde{\mu }\left(s\right)d\tilde{\mu }\left(t\right)+\text{Re}\;\int V\left(s\right)d\tilde{\mu }\left(s\right) \end{array}$$

among all probability measures $\tilde{\mu }$ supported on supp(μ). Here, we require in addition that (145) is satisfied. This extra condition characterizes supp(μ) as a so‐called *S*‐curve.^46^, ^47^, ^48^, ^49^, ^50^, ^51^

### 8.1 Definition of Q and related computations

By taking the derivative in (143), we have

(146) $$\begin{array}{c} g_{+}^{\prime }\left(\zeta \right)+g_{-}^{\prime }\left(\zeta \right)-V^{\prime }\left(\zeta \right)=0,\;\zeta \in \mathrm{supp}\left(\mu \right), \end{array}$$

and by condition (b), $g^{\prime }$ is analytic in $C\setminus \bar{\mathrm{supp}(\mu )}$. Therefore, the function

(147) $$\begin{array}{c} Q\left(\zeta \right):={\left(g^{\prime }\left(\zeta \right)-\frac{V^{\prime }\left(\zeta \right)}{2}\right)}^{2} \end{array}$$

is meromorphic on C. By (142), we get

(148) $$\begin{array}{c} V^{\prime }\left(\zeta \right)=\frac{1}{\zeta }+\frac{1}{\zeta -c^{-1}}+\frac{1}{\zeta -c}-\frac{1}{\zeta -\alpha c^{-1}}-\frac{1}{\zeta -\alpha c}, \end{array}$$

from which we conclude that Q has a double zero at ∞, and double poles at 0, αc, $\alpha c^{-1}$, *c*, and $c^{-1}$. Because a meromorphic function on the Riemann sphere (genus 0) has as many poles as zeros, Q has eight other zeros. As ζ→∞, we have $g^{\prime }\left(\zeta \right)=\zeta^{-1}+O\left(\zeta^{-2}\right)$, from which we get $Q\left(\zeta \right)=2^{-2}\zeta^{-2}+O\left(\zeta^{-3}\right)$. Therefore, Q can be written in the form

(149) $$Q\left(\zeta \right)=\frac{\Pi (\zeta )}{4\zeta^{2}{\left(\zeta -\alpha c\right)}^{2}{\left(\zeta -\alpha c^{-1}\right)}^{2}{\left(\zeta -c\right)}^{2}{\left(\zeta -c^{-1}\right)}^{2}},$$

where Π is a monic polynomial of degree 8 which remains to be determined. If we assume that $g^{\prime }\left(\zeta \right)$ remains bounded for ζ∈C, then we can deduce from (147) and (148) the leading order term for Q(ζ) as $\zeta \to \zeta_{*}\in \left\{0,\alpha c,\alpha c^{-1},c,c^{-1}\right\}$:

(150) $$\begin{array}{cccc} & Q\left(\zeta \right)=2^{-2}\zeta^{-2}+O\left(\zeta^{-1}\right), & & \;\text{as}\;\zeta \to 0, \end{array}$$

(151) $$\begin{array}{cccc} & Q\left(\zeta \right)=2^{-2}{\left(\zeta -\alpha c\right)}^{-2}+O\left({\left(\zeta -\alpha c\right)}^{-1}\right), & & \;\text{as}\;\zeta \to \alpha c, \end{array}$$

(152) $$\begin{array}{cccc} & Q\left(\zeta \right)=2^{-2}{\left(\zeta -\alpha c^{-1}\right)}^{-2}+O\left({\left(\zeta -\alpha c^{-1}\right)}^{-1}\right), & & \;\text{as}\;\zeta \to \alpha c^{-1}, \end{array}$$

(153) $$\begin{array}{cccc} & Q\left(\zeta \right)=2^{-2}{\left(\zeta -c\right)}^{-2}+O\left({\left(\zeta -c\right)}^{-1}\right), & & \;\text{as}\;\zeta \to c, \end{array}$$

(154) $$\begin{array}{cccc} & Q\left(\zeta \right)=2^{-2}{\left(\zeta -c^{-1}\right)}^{-2}+O\left({\left(\zeta -c^{-1}\right)}^{-1}\right), & & \;\text{as}\;\zeta \to c^{-1}. \end{array}$$

By combining these asymptotics with (149), we get

(155) $$\begin{array}{cccccc} & \Pi \left(0\right)=\alpha^{4}, & & \Pi \left(\alpha c\right)={\left(1-\alpha \right)}^{8}c^{8}, & & \Pi \left(\alpha c^{-1}\right)={\left(1-\alpha \right)}^{8}\alpha^{4},\\ & \Pi \left(c\right)={\left(1-\alpha \right)}^{8}c^{8}, & & \Pi \left(c^{-1}\right)={\left(1-\alpha \right)}^{8}\alpha^{-4}. \end{array}$$

This gives five linear equations for the eight unknown coefficients of Π, which is not enough to determine Π (and hence, Q). Therefore, one needs to make a further assumption: we assume that we can find Π in the form

(156) $$\Pi \left(\zeta \right)={\left(\zeta -r_{1}\right)}^{2}{\left(\zeta -r_{2}\right)}^{2}{\left(\zeta -r_{3}\right)}^{2}\left(\zeta -r_{+}\right)\left(\zeta -r_{-}\right).$$

As we will see, Assumption (156) implies that supp(μ) consists of a single curve (“one‐cut regime”). This assumption is justified if we can: (1) find *r*_1_, *r*_2_, *r*_3_, $r_{+}$, $r_{-}$ so that (155) holds and (2) construct a *g*‐function via (147), which satisfies the properties (a), (b), and (c).

Substituting (156) in (155), we obtain five *nonlinear* equations for the five unknowns *r*_1_, *r*_2_, *r*_3_, $r_{+}$, $r_{-}$. This system turns out to have quite a few solutions—we need to select “the correct one.” Let us define *r*_1_, *r*_2_, *r*_3_, $r_{+}$, $r_{-}$ by (33) and (34). It is a simple computation to verify that indeed (155) holds in this case. We will show in Section 8.4 that this definition of *r*_1_, *r*_2_, *r*_3_, $r_{+}$, $r_{-}$ is “the correct solution” to (155), in the sense that it allows to construct a *g*‐function satisfying the properties (a), (b), and (c).

Remark 5 Let us briefly comment on how to find (33) and (34). Unfortunately, we were not able to solve analytically the nonlinear system obtained after substituting (156) into (155). Instead, we have solved numerically (using the Newton–Raphson method) this system for a large number of values of α∈(0,1). As already mentioned, the system (155) possesses several solutions. To ensure numerical convergence to “the correct solution,” we choose starting values of *r*_1_, *r*_2_, and *r*_3_ so that (35) holds. The expressions (33) and (34) have then been guessed by an inspection of the plots of $r_{1}\left(\alpha \right)$, $r_{2}\left(\alpha \right)$, $r_{3}\left(\alpha \right)$, $r_{+}\left(\alpha \right)$, $r_{-}\left(\alpha \right)$.

### 8.2 Critical trajectories of Q

In this subsection, we study the critical trajectories of Q, which are relevant to define the *g*‐function and study its properties.

Let t↦ζ(t), t∈[a,b] be a smooth parameterization of a curve σ, satisfying $\zeta^{\prime }\left(t\right)\neq 0$ for all t∈(a,b). σ is a *trajectory* of the quadratic differential $Q\left(\zeta \right)d\zeta^{2}$ if $Q\left(\zeta \left(t\right)\right)\zeta^{\prime }{\left(t\right)}^{2}<0$ for every t∈(a,b), and an *orthogonal trajectory* if $Q\left(\zeta \left(t\right)\right)\zeta^{\prime }{\left(t\right)}^{2}>0$ for every t∈(a,b). σ is *critical* if it contains a zero or a pole of Q. Note that these definitions are independent of the choice of the parameterization.

Because $r_{+}$ and $r_{-}$ are simple zeros of Q, there are three critical trajectories (and also three orthogonal critical trajectories) emanating from each of the points $r_{\pm }$. Recall the definitions of $\gamma_{0},\gamma_{\alpha },\gamma_{1},\Sigma_{0},\Sigma_{\alpha }$, and Σ_1_ given in Section 3.4.

**FIGURE 10:**
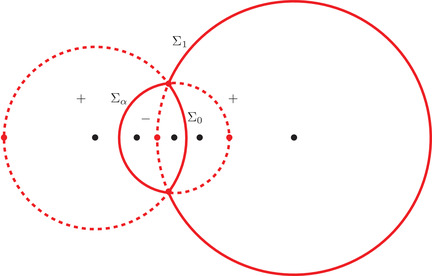
The critical trajectories of Q (solid red), and the critical orthogonal trajectories (dashed red) for α=0.4. The red dots are the zeros of Q, and the black dots are the poles. The critical trajectories divide C in three regions. The sign of Reϕ in each of these regions in shown by + or −

Lemma 8 The arcs $\bar{\Sigma_{0}}$, $\bar{\Sigma_{\alpha }}$, and $\bar{\Sigma_{1}}$ are three critical trajectories of $Q\left(\zeta \right)d\zeta^{2}$ joining $r_{-}$ with $r_{+}$, and $\gamma_{0}\setminus \Sigma_{0}$, $\gamma_{\alpha }\setminus \Sigma_{\alpha }$, and $\gamma_{1}\setminus \Sigma_{1}$ are each the union of two critical orthogonal trajectories of $Q\left(\zeta \right)d\zeta^{2}$. An illustration is shown in Figure 10.

Let $t\mapsto \zeta =\zeta \left(t\right)=c^{-1}+R_{1}e^{it}$, t∈[−π,π], be a parameterization of γ_1_. Writing $r_{\pm }=c^{-1}+R_{1}e^{\pm i\theta_{1}}$ with $\theta_{1}\in \left(\frac{2\pi }{3},\pi \right)$, and noting that $\zeta^{\prime }=iRe^{it}$, we have

$$\begin{array}{c} \left(\zeta -r_{+}\right)\left(\zeta -r_{-}\right)=2R_{1}^{2}e^{it}\left(\cos t-\cos\theta_{1}\right)\;\;\text{and}\;\;\frac{{\left(\zeta^{\prime }\right)}^{2}}{{\left(\zeta -c^{-1}\right)}^{2}}=-1. \end{array}$$

Therefore, we get

(157) $$\begin{array}{cc} Q\left(\zeta \right){\left(\zeta^{\prime }\right)}^{2} & \;={\left(\zeta^{\prime }\right)}^{2}\frac{{\left(\zeta -r_{1}\right)}^{2}{\left(\zeta -r_{2}\right)}^{2}{\left(\zeta -r_{3}\right)}^{2}\left(\zeta -r_{+}\right)\left(\zeta -r_{-}\right)}{4\zeta^{2}{\left(\zeta -\alpha c\right)}^{2}{\left(\zeta -\alpha c^{-1}\right)}^{2}{\left(\zeta -c\right)}^{2}{\left(\zeta -c^{-1}\right)}^{2}}\\ & \;=-R_{1}^{2}e^{it}\frac{{\left(\zeta -r_{1}\right)}^{2}{\left(\zeta -r_{2}\right)}^{2}{\left(\zeta -r_{3}\right)}^{2}\left(\cos t-\cos\theta_{1}\right)}{2\zeta^{2}{\left(\zeta -\alpha c\right)}^{2}{\left(\zeta -\alpha c^{-1}\right)}^{2}{\left(\zeta -c\right)}^{2}}. \end{array}$$

Using (43), we show that $\left(\zeta -r_{2}\right)=2R_{1}e^{\frac{it}{2}}\cos\frac{t}{2}$, and

$$\begin{array}{cc} \left(\zeta -r_{1}\right)\left(\zeta -r_{3}\right) & \;=2R_{1}^{2}e^{it}\left(\cos t+\frac{\alpha^{2}+\left(2-\alpha \right)\sqrt{1-\alpha +\alpha^{2}}}{2(1-\alpha )}\right),\\ \zeta (\zeta -c) & \;=2R_{1}^{2}e^{it}\left(\cos t+\frac{2-3\alpha +2\alpha^{2}}{2\left(1-\alpha \right)\sqrt{1-\alpha +\alpha^{2}}}\right),\\ \left(\zeta -\alpha c\right)\left(\zeta -\alpha c^{-1}\right) & \;=2R_{1}^{2}e^{it}\left(\cos t+\frac{2-\alpha +\alpha^{2}}{2\sqrt{1-\alpha +\alpha^{2}}}\right). \end{array}$$

Substituting the above expressions in (157), we find

(158) $$\begin{array}{c} Q\left(\zeta \right){\left(\zeta^{\prime }\right)}^{2}=\frac{\left(\cos\theta_{1}-\cos t\right)\cos^{2}\frac{t}{2}{\left(\cos t+\frac{\alpha^{2}+\left(2-\alpha \right)\sqrt{1-\alpha +\alpha^{2}}}{2(1-\alpha )}\right)}^{2}}{2{\left(\cos t+\frac{2-3\alpha +2\alpha^{2}}{2\left(1-\alpha \right)\sqrt{1-\alpha +\alpha^{2}}}\right)}^{2}{\left(\cos t+\frac{2-\alpha +\alpha^{2}}{2\sqrt{1-\alpha +\alpha^{2}}}\right)}^{2}}. \end{array}$$

We verify by direct computations that

$$\begin{array}{c} \frac{\alpha^{2}+\left(2-\alpha \right)\sqrt{1-\alpha +\alpha^{2}}}{2(1-\alpha )}>\frac{2-3\alpha +2\alpha^{2}}{2\left(1-\alpha \right)\sqrt{1-\alpha +\alpha^{2}}}>\frac{2-\alpha +\alpha^{2}}{2\sqrt{1-\alpha +\alpha^{2}}}>1, \end{array}$$

and thus the right‐hand side of (158) is negative for $t\in (-\theta_{1},\theta_{1})$, positive for $t\in \left(-\pi ,-\theta_{1}\right)\cup \left(\theta_{1},\pi \right)$, and zero for $t=-\pi ,-\theta_{1},\theta_{1},\pi$. We conclude that $\bar{\Sigma_{1}}$ is a critical trajectory and that $\gamma_{1}\setminus \Sigma_{1}$ is the union of two orthogonal critical trajectories. The statement about $\bar{\Sigma_{\alpha }}$, $\gamma_{\alpha }\setminus \Sigma_{\alpha }$, $\bar{\Sigma_{0}}$, $\gamma_{0}\setminus \Sigma_{0}$ can be proved a similar way, and we provide less details. For $\zeta =\zeta \left(t\right)=R_{0}e^{it}$, t∈[−π,π], after long but straightforward computations, we obtain

(159) $$\begin{array}{c} Q\left(\zeta \right){\left(\zeta^{\prime }\right)}^{2}=\frac{\left(\cos\theta_{0}-\cos t\right)\cos^{2}\frac{t}{2}{\left(\cos t-\frac{\left(1+\alpha \right)\sqrt{1-\alpha +\alpha^{2}}-{\left(1-\alpha \right)}^{2}}{2\alpha }\right)}^{2}}{2{\left(\cos t-\frac{1-\alpha +2\alpha^{2}}{2\alpha \sqrt{1-\alpha +\alpha^{2}}}\right)}^{2}{\left(\cos t-\frac{2-\alpha +\alpha^{2}}{2\sqrt{1-\alpha +\alpha^{2}}}\right)}^{2}}. \end{array}$$

Because

$$\begin{array}{c} \frac{1-\alpha +2\alpha^{2}}{2\alpha \sqrt{1-\alpha +\alpha^{2}}}>\frac{\left(1+\alpha \right)\sqrt{1-\alpha +\alpha^{2}}-{\left(1-\alpha \right)}^{2}}{2\alpha }>\frac{2-\alpha +\alpha^{2}}{2\sqrt{1-\alpha +\alpha^{2}}}>1, \end{array}$$

we infer that $\bar{\Sigma_{0}}$ is a critical trajectory and that $\gamma_{0}\setminus \Sigma_{0}$ is the union of two orthogonal critical trajectories. For $\zeta =\zeta \left(t\right)=\alpha c^{-1}+R_{\alpha }e^{it}$, t∈[−π,π], we obtain

(160) $$\begin{array}{c} Q\left(\zeta \right){\left(\zeta^{\prime }\right)}^{2}=\frac{\left(\cos t-\cos\theta_{\alpha }\right)\sin^{2}\frac{t}{2}{\left(\cos t+\frac{1-\left(1-2\alpha \right)\sqrt{1-\alpha +\alpha^{2}}}{2\alpha (1-\alpha )}\right)}^{2}}{2{\left(\cos t-\frac{1-\alpha +2\alpha^{2}}{2\alpha \sqrt{1-\alpha +\alpha^{2}}}\right)}^{2}{\left(\cos t+\frac{2-3\alpha +2\alpha^{2}}{2\left(1-\alpha \right)\sqrt{1-\alpha +\alpha^{2}}}\right)}^{2}} \end{array}$$

with

$$\begin{array}{c} \frac{1-\left(1-2\alpha \right)\sqrt{1-\alpha +\alpha^{2}}}{2\alpha (1-\alpha )}>\frac{2-3\alpha +2\alpha^{2}}{2\left(1-\alpha \right)\sqrt{1-\alpha +\alpha^{2}}}>1,\;\frac{1-\alpha +2\alpha^{2}}{2\alpha \sqrt{1-\alpha +\alpha^{2}}}>1. \end{array}$$

Therefore, we deduce from an inspection of (160) that $\bar{\Sigma_{\alpha }}$ is a critical trajectory and that $\gamma_{\alpha }\setminus \Sigma_{\alpha }$ is the union of two orthogonal critical trajectories. This finishes the proof.▪

### 8.3 Branch cut structure and the zero set of Reϕ

As can be seen in (147), $g^{\prime }$ can be expressed as

(161) $$\begin{array}{c} g^{\prime }\left(\zeta \right)=\frac{V^{\prime }\left(\zeta \right)}{2}+Q{\left(\zeta \right)}^{1/2}, \end{array}$$

for a certain branch of $Q{\left(\zeta \right)}^{1/2}$. To obtain a *g*‐function with the desired properties (a), (b), and (c), it turns out that the branch cut needs to be taken along the critical trajectory Σ_1_ (as in Section 3.4).

Definition 3 We define $Q^{1/2}$ as

(162) $$\begin{array}{c} Q{\left(\zeta \right)}^{1/2}=\frac{\left(\zeta -r_{1}\right)\left(\zeta -r_{2}\right)\left(\zeta -r_{3}\right)\sqrt{\left(\zeta -r_{+}\right)\left(\zeta -r_{-}\right)}}{2\zeta \left(\zeta -\alpha c\right)\left(\zeta -\alpha c^{-1}\right)\left(\zeta -c\right)\left(\zeta -c^{-1}\right)}, \end{array}$$

where the branch cut for $\sqrt{\left(\zeta -r_{+}\right)\left(\zeta -r_{-}\right)}$ is taken on Σ_1_ such that

$$\begin{array}{c} \sqrt{\left(\zeta -r_{+}\right)\left(\zeta -r_{-}\right)}=\zeta +O\left(1\right),\;\text{as}\;\zeta \to \infty . \end{array}$$

It will also be convenient to define a primitive of $Q^{1/2}$.

Definition 4 We define $\phi :C\setminus (\left(-\infty ,c^{-1}\right]\cup \left\{c^{-1}+R_{1}e^{it}:-\pi \leq t\leq \theta_{1}\right\})\to C$ by

(163) $$\phi \left(\zeta \right)=\int_{r_{+}}^{\zeta }Q{\left(\xi \right)}^{1/2}d\xi ,$$

where the path of integration does not intersect $\left(-\infty ,c^{-1}\right]\cup \left\{c^{-1}+R_{1}e^{it}:-\pi \leq t\leq \theta_{1}\right\}$.

We first state some basic properties of ϕ. By (150)–(154), $Q^{1/2}$ has simple poles at 0, αc, $\alpha c^{-1}$, *c*, and $c^{-1}$, and the residues are real. Also, because Σ_1_ is a critical trajectory of Q, we have $\phi_{\pm }\left(\zeta \right)\in iR$ for $\zeta \in \Sigma_{1}$. Therefore, Reϕ is single‐valued and continuous in $C\setminus \{0,\alpha c,\alpha c^{-1},c,c^{-1}\}$, and Reϕ is also harmonic in $C\setminus (\Sigma_{1}\cup \left\{0,\alpha c,\alpha c^{-1},c,c^{-1}\right\})$. Finally, by combining Definition 3 with (150)–(154), we have

(164) $$\begin{array}{ccc} \phi (\zeta ) & =-\frac{1}{2}\log\zeta +O\left(1\right)\;\text{as}\;\zeta \to 0, & \lim_{\zeta \to 0}\text{Re}\;\phi \left(\zeta \right)=+\infty ,\\ \phi (\zeta ) & =\frac{1}{2}\log\left(\zeta -\alpha c\right)+O\left(1\right)\;\text{as}\;\zeta \to \alpha c, & \lim_{\zeta \to \alpha c}\text{Re}\;\phi \left(\zeta \right)=-\infty ,\\ \phi (\zeta ) & =\frac{1}{2}\log\left(\zeta -\alpha c^{-1}\right)+O\left(1\right)\;\text{as}\;\zeta \to \alpha c^{-1}, & \lim_{\zeta \to \alpha c^{-1}}\text{Re}\;\phi \left(\zeta \right)=-\infty ,\\ \phi (\zeta ) & =-\frac{1}{2}\log\left(\zeta -c\right)+O\left(1\right)\;\text{as}\;\zeta \to c, & \lim_{\zeta \to c}\text{Re}\;\phi \left(\zeta \right)=+\infty ,\\ \phi (\zeta ) & =-\frac{1}{2}\log\left(\zeta -c^{-1}\right)+O\left(1\right)\;\text{as}\;\zeta \to c^{-1}, & \lim_{\zeta \to c^{-1}}\text{Re}\;\phi \left(\zeta \right)=+\infty ,\\ \phi (\zeta ) & =\frac{1}{2}\log\left(\zeta \right)+O\left(1\right)\;\text{as}\;\zeta \to \infty , & \lim_{\zeta \to \infty }\text{Re}\;\phi \left(\zeta \right)=+\infty . \end{array}$$

In the rest of this subsection, we determine the zero set $N_{\phi }$ of Reϕ. This will be useful in Section 8.4 to establish the (a), (b), and (c) properties of the *g*‐function. Let us define

(165) $$N_{\phi }=\left\{z\in C:\text{Re}\;\phi \left(z\right)=0\right\}.$$

Lemma 9 We have

(166) $$\begin{array}{c} N_{\phi }=\Sigma_{0}\cup \Sigma_{\alpha }\cup \Sigma_{1}. \end{array}$$

In particular, $N_{\phi }$ divides the complex plane in three regions. The sign of Reϕ in these regions is as shown in Figure 10.

By Lemma 8, it holds that

(167) $$\begin{array}{c} N_{\phi }⊇\Sigma_{0}\cup \Sigma_{\alpha }\cup \Sigma_{1}. \end{array}$$

We now prove the inclusion ⊆. We first show that

(168) $$\begin{array}{c} N_{\phi }\cap R=\left(\Sigma_{0}\cup \Sigma_{\alpha }\cup \Sigma_{1}\right)\cap R=\left\{\alpha c^{-1}-R_{\alpha },R_{0},c^{-1}+R_{1}\right\}. \end{array}$$

By Definitions 3 and 4, $\phi^{\prime }=Q_{\alpha }^{1/2}$ changes sign when it crosses each of the nine points *r*_1_, 0, αc, *r*_2_, $\alpha c^{-1}$, *c*, *r*_3_, $c^{-1}$, $c^{-1}+R_{1}$. Because $\phi^{\prime }\left(\zeta \right)=2^{-1}\zeta^{-1}+O\left(\zeta^{-2}\right)$ as ζ→∞, we have $\phi^{\prime }>0$ on the intervals

$$\begin{array}{c} \left(r_{1},0\right),\;\left(\alpha c,r_{2}\right),\;\left(\alpha c^{-1},c\right),\;\left(r_{3},c^{-1}\right),\;\left(c^{-1}+R_{1},+\infty \right), \end{array}$$

and $\phi^{\prime }<0$ on the intervals

$$\begin{array}{c} \left(-\infty ,r_{1}\right),\;\left(0,\alpha c\right),\;\left(r_{2},\alpha c^{-1}\right),\;\left(c,r_{3}\right),\;\left(c^{-1},c^{-1}+R_{1}\right). \end{array}$$

By (167), we have

$$\begin{array}{c} \text{Re}\;\phi \left(\alpha c^{-1}-R_{\alpha }\right)=\text{Re}\;\phi \left(R_{0}\right)=\text{Re}\;\phi \left(c^{-1}+R_{1}\right)=0, \end{array}$$

so Reϕ admits no other zeros on $\left(0,\alpha c\right)\cup \left(\alpha c^{-1},c\right)\cup \left(c^{-1},+\infty \right)$. On the intervals (−∞,0) and $\left(c,c^{-1}\right)$, Reϕ admits a local minimum at *r*_1_ and *r*_3_, respectively, and on the interval $\left(\alpha c,\alpha c^{-1}\right)$, it admits a local maximum at *r*_2_. Thus, (168) holds true if we show that

(169) $$\begin{array}{c} \text{Re}\;\phi \left(r_{1}\right)>0,\;\text{Re}\;\phi \left(r_{2}\right)<0,\;\;\text{and}\;\;\text{Re}\;\phi \left(r_{3}\right)>0. \end{array}$$

By Lemma 8, Reϕ is strictly monotone on each of the curves $\left(\gamma_{0}\setminus \Sigma_{0}\right)\cap C^{+}$, $\left(\gamma_{\alpha }\setminus \Sigma_{\alpha }\right)\cap C^{+}$, and $\left(\gamma_{1}\setminus \Sigma_{1}\right)\cap C^{+}$. The expressions (158), (159), and (160), together with Definition 3, allow to conclude that Reϕ is strictly increasing on $\left(\gamma_{0}\setminus \Sigma_{0}\right)\cap C^{+}$ oriented from $r_{+}$ to *r*_1_, strictly increasing on $\left(\gamma_{\alpha }\setminus \Sigma_{\alpha }\right)\cap C^{+}$ oriented from $r_{+}$ to *r*_3_, and strictly decreasing on $\left(\gamma_{1}\setminus \Sigma_{1}\right)\cap C^{+}$ oriented from $r_{+}$ to *r*_2_. In particular this proves (169), and thus (168).

Assume $N_{\phi }$ if of the form $\Sigma_{0}\cup \Sigma_{\alpha }\cup \Sigma_{1}\cup \sigma$ for a certain curve σ distinct from Σ_0_, $\Sigma_{\alpha }$, and Σ_1_. Because $\phi_{\pm }^{\prime }\left(\zeta \right)\neq 0$ for $\zeta \in \Sigma_{1}$, we must have $\sigma \cap \Sigma_{1}=\emptyset$. Also, in view of (168), σ cannot intersect the real axis. Then, σ must be a closed contour in $C\setminus (R\cup \Sigma_{1})$, and the max/min principle for harmonic functions would then imply that Reϕ in constant on the whole bounded region delimited by σ. By (163), Reϕ is clearly not constant on such domain, so we arrive at a contradiction, and we conclude that $N_{\phi }=\Sigma_{0}\cup \Sigma_{\alpha }\cup \Sigma_{1}$.

Thus, $N_{\phi }$ divides the complex plane in three regions in which Reϕ does not change sign. The signs in each of these regions is then determined immediately by (164) (or equivalently, by (169)).▪

### 8.4 Definition and properties of *g*

Definition 5 We define the measure μ by

(170) $$\begin{array}{cc} d\mu (\zeta ) & =\frac{1}{\pi i}Q_{-}{\left(\zeta \right)}^{1/2}d\zeta \\ & =\frac{1}{\pi i}\frac{\left(\zeta -r_{1}\right)\left(\zeta -r_{2}\right)\left(\zeta -r_{3}\right){\sqrt{\left(\zeta -r_{+}\right)\left(\zeta -r_{-}\right)}}_{-}}{2\zeta \left(\zeta -\alpha c\right)\left(\zeta -\alpha c^{-1}\right)\left(\zeta -c\right)\left(\zeta -c^{-1}\right)}d\zeta ,\;\zeta \in \Sigma_{1}, \end{array}$$

where $\Sigma_{1}=\mathrm{supp}\left(\mu \right)$ is given by (44), and is oriented from $r_{-}$ to $r_{+}$; so $Q_{-}{\left(\zeta \right)}^{1/2}$ denotes the limit of $Q{\left(\xi \right)}^{1/2}$ as $\xi \to \zeta \in \Sigma_{1}$ with ξ in the exterior of the circle γ_1_.

Proposition 13 The measure μ defined in (170) is a probability measure.

We compute $\int_{\Sigma_{1}}d\mu$ by residue calculation. Because $Q_{+}=-Q_{-}$, we have

(171) $$\begin{array}{c} \int_{\Sigma_{1}}d\mu \left(\zeta \right)=\frac{1}{2\pi i}\int_{C}Q{\left(\zeta \right)}^{1/2}d\zeta , \end{array}$$

where C is a closed curve surrounding Σ_1_ once in the positive direction, but not surrounding any of the poles of Q. By deforming C into another contour $\tilde{C}$ surrounding 0, αc, $\alpha c^{-1}$, *c*, and $c^{-1}$, we pick up some residues:

(172) $$\begin{array}{c} \int_{\Sigma_{1}}d\mu \left(\zeta \right)=-\sum_{\zeta_{*}\in P}\text{Res}\left(Q{\left(\zeta \right)}^{1/2},\zeta =\zeta_{*}\right)+\frac{1}{2\pi i}\int_{\tilde{C}}Q{\left(\zeta \right)}^{1/2}d\zeta , \end{array}$$

where $P=\{0,\alpha c,\alpha c^{-1},c,c^{-1}\}$. By combining Definition 3 with (150)–(154), we have

(173) $$\begin{array}{cccc} & \text{Res}\left(Q{\left(\zeta \right)}^{1/2},\zeta =0\right)=-\frac{1}{2}, & & \text{Res}\left(Q{\left(\zeta \right)}^{1/2},\zeta =\alpha c\right)=\frac{1}{2},\\ & \text{Res}\left(Q{\left(\zeta \right)}^{1/2},\zeta =\alpha c^{-1}\right)=\frac{1}{2}, & & \text{Res}\left(Q{\left(\zeta \right)}^{1/2},\zeta =c\right)=-\frac{1}{2},\\ & \text{Res}\left(Q{\left(\zeta \right)}^{1/2},\zeta =c^{-1}\right)=-\frac{1}{2}, \end{array}$$

and because $Q{\left(\zeta \right)}^{1/2}=\frac{1}{2\zeta }+O\left(\zeta^{-2}\right)$ as ζ→∞, we find

(174) $$\begin{array}{c} \int_{\Sigma_{1}}d\mu \left(\zeta \right)=1. \end{array}$$

It remains to show that μ has a positive density on Σ_1_. Let $\zeta \left(t\right)=c^{-1}+R_{1}e^{it}$, $-\theta_{1}<t<\theta_{1}$, be a parameterization of Σ_1_. Consider the function

(175) $$t\mapsto \int_{r_{-}}^{\zeta (t)}d\mu =\frac{1}{\pi i}\int_{r_{-}}^{\zeta (t)}Q_{-}{\left(\xi \right)}^{1/2}d\xi ,$$

whose derivative is given by

(176) $$\begin{array}{c} \frac{1}{\pi i}Q_{-}{\left(\zeta \left(t\right)\right)}^{1/2}\zeta^{\prime }\left(t\right). \end{array}$$

Because $Q\left(\zeta \left(t\right)\right){\left(\zeta^{\prime }\left(t\right)\right)}^{2}<0$ for $t\in (-\theta_{1},\theta_{1})$ by Lemma 8, (176) is real and nonzero. Note also that the function (175) vanishes for $t=-\theta_{1}$ and equals 1 for $t=\theta_{1}$ by (174). Therefore, (176) is strictly positive.▪

Definition 6 The *g*‐function is defined by

(177) $$g\left(\zeta \right)=\int_{\Sigma_{1}}\log\left(\zeta -\xi \right)d\mu \left(\xi \right),\;\zeta \in C\setminus \left(\left(-\infty ,r_{2}\right]\cup \left\{c^{-1}+R_{1}e^{it}:-\pi \leq t\leq \theta_{1}\right\}\right),$$

where for each $\xi \in \Sigma_{1}$, the function ζ↦log(ζ−ξ) has a branch cut along $\left(-\infty ,r_{2}\right]\cup \left\{c^{-1}+R_{1}e^{it}:-\pi \leq t\leq \arg\xi \right\}$ and behaves like $\log\left(\zeta -\xi \right)=\log|\zeta |+O\left(\zeta^{-1}\right)$, as ζ→+∞.

We define the variational constant ℓ∈C by

(178) $$\begin{array}{c} \ell =-2g\left(r_{+}\right)+V\left(r_{+}\right). \end{array}$$

The next proposition shows, among other things, that Definition 6 for *g* is consistent with (161), and that *g* satisfies (143).

Proposition 14 The functions *g* and ϕ are related by

(179) $$\begin{array}{c} \phi \left(\zeta \right)=g\left(\zeta \right)-\frac{V(\zeta )}{2}+\frac{\ell }{2},\;\zeta \in C\setminus \left(\left(-\infty ,r_{2}\right]\cup \left\{c^{-1}+R_{1}e^{it}:-\pi \leq t\leq \theta_{1}\right\}\right) \end{array}$$

and we have

(180) $$\begin{array}{cccc} & g_{+}\left(\zeta \right)+g_{-}\left(\zeta \right)-V\left(\zeta \right)+\ell =0, & & \text{for}\;\zeta \in \Sigma_{1}, \end{array}$$

(181) $$\begin{array}{cccc} & g_{+}\left(\zeta \right)-g_{-}\left(\zeta \right)=2\phi_{+}\left(\zeta \right)=-2\phi_{-}\left(\zeta \right), & & \text{for}\;\zeta \in \Sigma_{1}. \end{array}$$

Furthermore, the *g*‐function satisfies the properties (a), (b), and (c) listed at the beginning of Section 8 with $\gamma_{C}=\gamma_{1}$.

We first prove (179). For a fixed $\zeta \in C\setminus \Sigma_{1}$, we have

$$\begin{array}{cc} & g^{\prime }\left(\zeta \right)=\int_{\Sigma_{1}}\frac{d\mu (\xi )}{\zeta -\xi }=\frac{1}{2\pi i}\int_{C}\frac{Q{\left(\xi \right)}^{1/2}}{\zeta -\xi }d\xi , \end{array}$$

where C is a closed curve surrounding Σ_1_ once in the positive direction, but not surrounding any of the poles of Q, and not surrounding ζ. By deforming C into another contour $\tilde{C}$ surrounding 0, αc, $\alpha c^{-1}$, *c*, $c^{-1}$, and ζ, we obtain

(182) $$\begin{array}{c} \int_{\Sigma_{1}}\frac{d\mu (\xi )}{\zeta -\xi }=-\sum_{\xi_{*}\in P}\text{Res}\left(\frac{Q{\left(\xi \right)}^{1/2}}{\zeta -\xi },\xi =\xi_{*}\right)+Q{\left(\zeta \right)}^{1/2}+\frac{1}{2\pi i}\int_{\tilde{C}}\frac{Q{\left(\xi \right)}^{1/2}}{\zeta -\xi }d\xi , \end{array}$$

where $P=\{0,\alpha c,\alpha c^{-1},c,c^{-1}\}$. By deforming $\tilde{C}$ to ∞, noting that $Q{\left(\xi \right)}^{1/2}=O\left(\xi^{-1}\right)$ as ξ→∞, the integral on the right‐hand side of (182) is 0. The sum can be evaluated using the residues (173), and we get

$$\begin{array}{c} \int_{\Sigma_{1}}\frac{d\mu (\xi )}{\zeta -\xi }=\frac{1}{2\zeta }-\frac{1}{2(\zeta -\alpha c)}-\frac{1}{2(\zeta -\alpha c^{-1})}+\frac{1}{2(\zeta -c)}+\frac{1}{2(\zeta -c^{-1})}+Q{\left(\zeta \right)}^{1/2}. \end{array}$$

Using (148) and $\phi^{\prime }=Q^{1/2}$, the above can be rewritten as

$$\begin{array}{c} g^{\prime }\left(\zeta \right)=\frac{V^{\prime }\left(\zeta \right)}{2}+\phi^{\prime }\left(\zeta \right),\;\zeta \in C\setminus \Sigma_{1}. \end{array}$$

Integrating this identity from $r_{+}$ to ζ along a path that does not intersect $\left(-\infty ,c^{-1}\right]\cup \left\{c^{-1}+R_{1}e^{it}:-\pi \leq t<\theta_{1}\right\}$, we obtain

$$\begin{array}{c} g\left(\zeta \right)-g\left(r_{+}\right)=\frac{V(\zeta )}{2}-\frac{V(r_{+})}{2}+\phi \left(\zeta \right), \end{array}$$

where we have used $\phi (r_{+})=0$. Then, (179) follows from the definition of ℓ given by (178). Because $Q_{+}^{1/2}=-Q_{-}^{1/2}$ on Σ_1_, by (163) we have

$$\begin{array}{cc} & \phi_{+}\left(\zeta \right)+\phi_{-}\left(\zeta \right)=0,\;\text{for}\;\zeta \in \Sigma_{1}, \end{array}$$

from which (180) and (181) follow. The circle γ_1_ encloses both *c* and $c^{-1}$, and 0 lies in the exterior of γ_1_, so criterion (a) is fulfilled. For $\zeta \in \left(-\infty ,r_{2}\right]\cup \left\{c^{-1}+R_{1}e^{it}:-\pi \leq -\theta_{1}\right\}$, we have

$$\begin{array}{c} g_{+}\left(\zeta \right)-g_{-}\left(\zeta \right)=\int_{\Sigma_{1}}\left(\log_{+}\left(\zeta -\xi \right)-\log_{-}\left(\zeta -\xi \right)\right)d\mu \left(\xi \right)=2\pi i\int_{\Sigma_{1}}d\mu \left(\xi \right)=2\pi i, \end{array}$$

so $e^{g}$ is analytic in $C\setminus \bar{\Sigma_{1}}$ and criterion (b) is also fulfilled. For $\zeta \in \gamma_{1}\setminus \bar{\Sigma_{1}}$, by (179) and Lemma 9, we have

$$\begin{array}{c} \text{Re}\;\left(g_{+}\left(\zeta \right)+g_{-}\left(\zeta \right)-V\left(\zeta \right)+\ell \right)=\text{Re}\;\left(\phi_{+}\left(\zeta \right)+\phi_{-}\left(\zeta \right)\right)=2\;\text{Re}\;\phi \left(\zeta \right)<0, \end{array}$$

as required in (144). Finally, by Definitions 4 and 5, for $\zeta \in \Sigma_{1}$ we have

$$\begin{array}{c} \text{Im}\;\left(g_{+}\left(\zeta \right)-\frac{V(\zeta )}{2}+\frac{\ell }{2}\right)=\text{Im}\;\phi_{+}\left(\zeta \right)=\text{Im}\;\int_{r_{+}}^{\zeta }Q_{+}^{1/2}\left(\xi \right)d\xi =\pi \int_{\zeta }^{r_{+}}d\mu \left(\xi \right), \end{array}$$

which is strictly decreasing as ζ goes from $r_{-}$ to $r_{+}$. So (145) holds as well, and hence (c), which finishes the proof.▪

## 9 STEEPEST DESCENT FOR *U*

In this section, we will perform an asymptotic analysis of the RH problem for *U* as N→+∞, by means of the Deift/Zhou steepest descent method.^22^ As mentioned in Section 8, the relevant contour to consider for the RH problem for *U* is $\gamma_{C}=\gamma_{1}$. The analysis is split in a series of transformations U↦T↦S↦R. The first transformation U↦T of Section 9.1 uses the *g*‐function obtained in Section 8 to normalize the RH problem at ∞. The opening of the lenses T↦S is realized in Section 9.2. The last step S↦R requires some preparations that are done in Section 9.3: it consists of constructing approximations (called “parametrices”) for *S* in different regions of the complex plane. Finally, the S↦R transformation is carried out in Section 9.4.

### 9.1 First transformation: U↦T

We normalize the RH problem with the following transformation:

(183) $$T\left(\zeta \right)=e^{N\ell \sigma_{3}}U\left(\zeta \right)e^{-2Ng\left(\zeta \right)\sigma_{3}}e^{-N\ell \sigma_{3}},$$

where *g* and ℓ are defined in Definition 6. Using (179), we can write the jumps for *T* in terms of the function ϕ of Definition 4. From (180) and (181), we find that *T* satisfies the following RH problem.

#### RH problem for *T*

(a) $T:C\setminus \gamma_{1}\to C^{2\times 2}$ is analytic.
(b) By using (142), (140), and (143), the jumps for *T* are given by
  (184) $$\begin{array}{cccc} & T_{+}\left(\zeta \right)=T_{-}\left(\zeta \right)\left(\begin{array}{cc} e^{-4N\phi_{+}\left(\zeta \right)} & 1\\ 0 & \;e^{-4N\phi_{-}\left(\zeta \right)} \end{array}\right), & & \;\text{for}\;\zeta \in \Sigma_{1}\subset \gamma_{1}, \end{array}$$
  (185) $$\begin{array}{cccc} & T_{+}\left(\zeta \right)=T_{-}\left(\zeta \right)\left(\begin{array}{cc} 1 & e^{2N(\phi_{+}\left(\zeta \right)+\phi_{-}\left(\zeta \right))}\\ 0 & 1 \end{array}\right), & & \;\text{for}\;\zeta \in \gamma_{1}\setminus \bar{\Sigma_{1}}. \end{array}$$
(c) As ζ→∞, we have $T\left(\zeta \right)=I+O\left(\zeta^{-1}\right)$. As ζ tends to $r_{+}$ or $r_{-}$, T(ζ) remains bounded.

The following estimates for *T* will be important for the saddle point analysis of Section 11.

Proposition 15 We have $T\left(\zeta \right)=O\left(N^{1/6}\right)$ and $T^{-1}\left(\zeta \right)=O\left(N^{1/6}\right)$ as N→∞, uniformly for $\zeta \in C\setminus \gamma_{1}$. In addition, for every δ>0 fixed, we have T(ζ)=O(1) and $T^{-1}\left(\zeta \right)=O\left(1\right)$ as N→∞ uniformly for

(186) $$\zeta \in \{\zeta \in C\setminus \gamma_{1}:|\zeta -r_{+}|\geq \delta ,|\zeta -r_{-}|\geq \delta \}.$$

The rest of this section is devoted to the proof of Proposition 15.

### 9.2 Second transformation: T↦S

**FIGURE 11:**
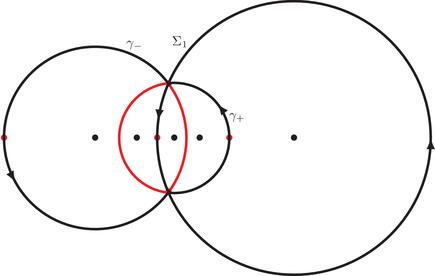
The jump contour for *T* (black), and $\Sigma_{\alpha }$ and Σ_0_ (in red), for α=0.4. The red dots are the zeros of Q, and the black dots are the poles

Note that for $\zeta \in \Sigma_{1}$, the jumps for *T* can be factorized as follows:

(187) $$\begin{array}{c} \left(\begin{array}{cc} e^{-4N\phi_{+}\left(\zeta \right)} & 1\\ 0 & e^{-4N\phi_{-}\left(\zeta \right)} \end{array}\right)=\left(\begin{array}{cc} 1 & 0\\ e^{-4N\phi_{-}\left(\zeta \right)} & 1 \end{array}\right)\left(\begin{array}{cc} 0 & 1\\ -1 & 0 \end{array}\right)\left(\begin{array}{cc} 1 & 0\\ e^{-4N\phi_{+}\left(\zeta \right)} & 1 \end{array}\right), \end{array}$$

where we used $\phi_{+}\left(\zeta \right)+\phi_{-}\left(\zeta \right)=0$ for $\zeta \in \Sigma_{1}$. We define the lenses $\gamma_{+}$ and $\gamma_{-}$ by

$$\begin{array}{cc} & \gamma_{+}:=\gamma_{\alpha }\setminus \bar{\Sigma_{\alpha }}\;\;\text{and}\;\;\gamma_{-}:=\gamma_{0}\setminus \bar{\Sigma_{0}}, \end{array}$$

see also Figure 11. The T↦S transformation is given by S(ζ)=T(ζ)W(ζ), where

(188) $$W\left(\zeta \right)=\left\{\begin{array}{cc} \left(\begin{array}{cc} 1 & 0\\ -e^{-4N\phi (\zeta )} & 1 \end{array}\right), & \text{for}\;\zeta \;\text{in}\;\text{the}\;\text{bounded}\;\text{region}\;\mathrm{delimited}\;\text{by}\;\bar{\Sigma_{1}\cup \gamma_{+}},\\ \left(\begin{array}{cc} 1 & 0\\ e^{-4N\phi (\zeta )} & 1 \end{array}\right), & \text{for}\;\zeta \;\text{in}\;\text{the}\;\text{unbounded}\;\text{region}\;\mathrm{delimited}\;\text{by}\;\bar{\Sigma_{1}\cup \gamma_{-}},\\ I, & \text{otherwise}. \end{array}\right.$$

*S* satisfies the following RH problem.

#### RH problem for *S*

(a) $S:C\setminus \left(\gamma_{1}\cup \gamma_{+}\cup \gamma_{-}\right)\to C^{2\times 2}$ is analytic.
(b) The jumps for *S* are given by
  (189) $$\begin{array}{cccc} & S_{+}\left(\zeta \right)=S_{-}\left(\zeta \right)\left(\begin{array}{cc} 0 & \;1\\ -1 & \;0 \end{array}\right), & & \;\text{for}\;\zeta \in \Sigma_{1}, \end{array}$$
  (190) $$\begin{array}{cccc} & S_{+}\left(\zeta \right)=S_{-}\left(\zeta \right)\left(\begin{array}{cc} 1 & \;0\\ e^{-4N\phi (\zeta )} & \;1 \end{array}\right), & & \;\text{for}\;\zeta \in \gamma_{+}\cup \gamma_{-}, \end{array}$$
  (191) $$\begin{array}{cccc} & S_{+}\left(\zeta \right)=S_{-}\left(\zeta \right)\left(\begin{array}{cc} 1 & \;e^{2N(\phi_{+}\left(\zeta \right)+\phi_{-}\left(\zeta \right))}\\ 0 & 1 \end{array}\right), & & \;\text{for}\;\zeta \in \gamma_{1}\setminus \bar{\Sigma_{1}}. \end{array}$$
(c) As ζ→∞, we have $S\left(\zeta \right)=I+O\left(\zeta^{-1}\right)$. As ζ tends to $r_{+}$ or $r_{-}$, S(ζ) remains bounded.

### 9.3 Parametrices

In this subsection, we find good approximations to *S* in different regions of the complex plane. By Lemma 9, Reϕ(ζ)>0 for $\zeta \in \gamma_{+}\cup \gamma_{-}$, Reϕ(ζ)<0 for $\zeta \in \gamma_{1}\setminus \bar{\Sigma_{1}}$, and Reϕ(ζ)=0 for $\zeta \in \Sigma_{1}$. So the jumps for *S* on $\gamma_{+}\cup \gamma_{-}\cup \left(\gamma_{1}\setminus \bar{\Sigma_{1}}\right)$ are exponentially close to the identity matrix matrix as N→∞, uniformly outside fixed neighborhoods of $r_{-}$ and $r_{+}$. By ignoring these jumps, we are left with the following RH problem, whose solution is denoted as $P^{\left(\infty \right)}$. We will show in Section 9.4 that $P^{\left(\infty \right)}$ is a good approximation to *S* away from $r_{+}$ and $r_{-}$.

#### RH problem for $P^{\left(\infty \right)}$

(a) $P^{\left(\infty \right)}:C\setminus \bar{\Sigma_{1}}\to C^{2\times 2}$ is analytic.
(b) The jumps for $P^{\left(\infty \right)}$ are given by
  (192) $$\begin{array}{cccc} & P_{+}^{\left(\infty \right)}\left(\zeta \right)=P_{-}^{\left(\infty \right)}\left(\zeta \right)\left(\begin{array}{cc} 0 & \;1\\ -1 & \;0 \end{array}\right), & & \;\text{for}\;\zeta \in \Sigma_{1}. \end{array}$$
(c) As ζ→∞, we have $P^{\left(\infty \right)}\left(\zeta \right)=I+O\left(\zeta^{-1}\right)$. As $\zeta \to \zeta_{*}\in \left\{r_{+},r_{-}\right\}$, $P^{\left(\infty \right)}\left(\zeta \right)=O\left({\left(\zeta -\zeta_{*}\right)}^{-1/4}\right)$.

The condition on the behavior of $P^{\left(\infty \right)}\left(\zeta \right)$ as $\zeta \to \zeta_{*}\in \left\{r_{+},r_{-}\right\}$ has been added to ensure existence of a solution. This RH problem is independent of *N*, and its unique solution is given by

(193) $$P^{\left(\infty \right)}\left(\zeta \right)=\left(\begin{array}{cc} \frac{1}{2}\left(a\left(\zeta \right)+a{\left(\zeta \right)}^{-1}\right) & \frac{1}{2i}\left(a\left(\zeta \right)-a{\left(\zeta \right)}^{-1}\right),\\ \frac{1}{-2i}\left(a\left(\zeta \right)-a{\left(\zeta \right)}^{-1}\right) & \frac{1}{2}\left(a\left(\zeta \right)+a{\left(\zeta \right)}^{-1}\right) \end{array}\right),$$

where $a\left(\zeta \right):={\left(\frac{\zeta -r_{+}}{\zeta -r_{-}}\right)}^{1/4}$ is analytic in $C\setminus \Sigma_{1}$ and such that a(ζ)∼1 as ζ→∞.

Note that $P^{\left(\infty \right)}$ is not a good approximation to *S* in small neighborhoods of $r_{+},r_{-}$; this can be seen from the behaviors

$$\begin{array}{cccc} & S\left(\zeta \right)=O\left(1\right)\;\;\text{and}\;\;P^{\left(\infty \right)}\left(\zeta \right)=O\left({\left(\zeta -\zeta_{*}\right)}^{-1/4}\right), & & \text{as}\;\zeta \to \zeta_{*}\in \left\{r_{+},r_{-}\right\}. \end{array}$$

Let δ>0 in Proposition 15 be fixed, and let $D_{r_{+}}$ and $D_{r_{-}}$ be small open disks of radius δ/2 centered at $r_{+}$ and $r_{-}$, respectively. We now construct local approximations $P^{\left(r_{+}\right)}$ and $P^{\left(r_{-}\right)}$ (called “local parametrices”) to *S* in $D_{r_{+}}$ and $D_{r_{-}}$, respectively. We require $P^{\left(r_{\pm }\right)}$ to satisfy the same jumps as *S* inside $D_{r_{\pm }}$, to remain bounded as $\zeta \to r_{\pm }$, and to satisfy the matching condition

(194) $$\begin{array}{c} P^{\left(r_{\pm }\right)}\left(\zeta \right)=\left(I+O\left(N^{-1}\right)\right)P^{\left(\infty \right)}\left(\zeta \right),\;\text{as}\;N\to +\infty , \end{array}$$

uniformly for $\zeta \in \partial D_{r_{\pm }}$. The density of μ vanishes like a square root at the endpoints $r_{+}$ and $r_{-}$, and therefore $P^{\left(r_{\pm }\right)}$ can be built in terms of Airy functions.^52^ These constructions are well known and standard, so we do not give the details. What is important for us is that

(195) $$P^{\left(r_{\pm }\right)}\left(z\right)=O\left(N^{\frac{1}{6}}\right),\;P^{\left(r_{\pm }\right)}{\left(z\right)}^{-1}=O\left(N^{\frac{1}{6}}\right)\;\;\text{as}\;N\to \infty ,$$

uniformly for $z\in D_{r_{\pm }}$.

### 9.4 Small norm RH problem *R*

The final transformation S↦R of the steepest descent is defined by

(196) $$R\left(\zeta \right)=\left\{\begin{array}{cc} S\left(\zeta \right)P^{\left(\infty \right)}{\left(\zeta \right)}^{-1}, & \;\;\text{for}\;\zeta \in C\setminus (\bar{D_{r_{+}}\cup D_{r_{-}}}),\\ S\left(\zeta \right)P^{\left(r_{+}\right)}{\left(\zeta \right)}^{-1}, & \;\;\text{for}\;\zeta \in D_{r_{+}},\\ S\left(\zeta \right)P^{\left(r_{-}\right)}{\left(\zeta \right)}^{-1}, & \;\;\text{for}\;\zeta \in D_{r_{-}}. \end{array}\right.$$

Because *S* and $P^{\left(r_{\pm }\right)}$ satisfy the same jumps inside $D_{r_{\pm }}$, *R* is analytic inside $\left(D_{r_{+}}\setminus \left\{r_{+}\right\}\right)\cup \left(D_{r_{-}}\setminus \left\{r_{-}\right\}\right)$. Furthermore, *S* and $P^{\left(r_{\pm }\right)}$ remain bounded near $r_{\pm }$, so the singularities of *R* at $r_{\pm }$ are removable. We conclude that *R* is analytic in

(197) $$\begin{array}{c} C\setminus \left(\left(\left(\gamma_{1}\cup \gamma_{+}\cup \gamma_{-}\right)\setminus \left(D_{r_{+}}\cup D_{r_{-}}\right)\right)\cup \partial D_{r_{+}}\cup \partial D_{r_{-}}\right). \end{array}$$

By (194), the jumps $R_{-}^{-1}R_{+}$ are $O(N^{-1})$ on $\partial D_{r_{+}}\cup \partial D_{r_{+}}$, and by Lemma 9, $R_{-}^{-1}R_{+}=O\left(e^{-cN}\right)$ on $\left(\gamma_{1}\cup \gamma_{+}\cup \gamma_{-}\right)\setminus \left(D_{r_{+}}\cup D_{r_{-}}\right)$ for a certain c>0. It follows by standard theory^52^, ^53^ that

(198) $$\begin{array}{c} R\left(\zeta \right)=I+O\left(N^{-1}\right),\;\text{as}\;N\to +\infty , \end{array}$$

uniformly for ζ in the domain (197). In particular, *R* and $R^{-1}$ remain bounded as N→∞.

Inverting the transformations (188) and (196), we get

$$\begin{array}{c} T\left(\zeta \right)=R\left(\zeta \right)\times \left\{\begin{array}{cc} P^{\left(\infty \right)}\left(\zeta \right), & \;\;\text{for}\;\zeta \in C\setminus (D_{r_{+}}\cup D_{r_{-}})\\ P^{\left(r_{+}\right)}\left(\zeta \right), & \;\;\text{for}\;\zeta \in D_{r_{+}}\\ P^{\left(r_{-}\right)}\left(\zeta \right), & \;\;\text{for}\;\zeta \in D_{r_{-}} \end{array}\right\}\times W{\left(\zeta \right)}^{-1}. \end{array}$$

By Lemma 9, W(ζ) and $W{\left(\zeta \right)}^{-1}$ are bounded as N→+∞, uniformly for ζ∈C. Proposition 15 follows then straightforwardly by using the estimates (195) and (198).

## 10 PHASE FUNCTIONS Φ AND Ψ

In Section 11, we will prove Proposition 11 via a saddle point analysis of the double contour integral (116). As it will turn out, the dominant part of the integrand as N→+∞ will be in the form $e^{2N(\Phi (\zeta ;\xi ,\eta )-\Phi (\omega ;\xi ,\eta ))}$, for a certain function Φ which is described below. The analytic continuation of Φ to the second sheet of $R_{\alpha }$ is denoted Ψ—it will also play a role in the saddle point analysis and is presented below.

The content of this section is a preparation for the saddle point analysis of Section 11. We will study the level set

(199) $$\begin{array}{c} N_{\Phi }=\left\{\zeta \in C:\text{Re}\;\Phi \left(\zeta \right)=\text{Re}\;\Phi \left(s\right)\right\}, \end{array}$$

and also find the relevant contour deformations to consider.

### 10.1 Preliminaries

We start with a definition.

Definition 7 For (ξ,η)∈H and $\zeta \in C\setminus (\left(-\infty ,c^{-1}\right]\cup \left\{c^{-1}+R_{1}e^{it}:-\pi \leq t\leq \theta_{1}\right\})$, we define Φ and Ψ by

(200) $$\begin{array}{cc} \Phi (\zeta )=\Phi (\zeta ;\xi ,\eta ) & =g\left(\zeta \right)-\frac{1+\xi -\eta }{2}\log\zeta +\frac{1+\xi }{2}\log\left(\left(\zeta -\alpha c\right)\left(\zeta -\alpha c^{-1}\right)\right)\\ & \;-\frac{1+\eta }{2}\log\left(\left(\zeta -c\right)\left(\zeta -c^{-1}\right)\right)+\frac{\ell }{2}\\ & =\phi \left(\zeta \right)-\frac{\xi -\eta }{2}\log\zeta +\frac{\xi }{2}\log\left(\left(\zeta -\alpha c\right)\left(\zeta -\alpha c^{-1}\right)\right)-\frac{\eta }{2}\log\left(\left(\zeta -c\right)\left(\zeta -c^{-1}\right)\right), \end{array}$$

(201) $$\begin{array}{cc} \Psi (\zeta ) & =\Psi (\zeta ;\xi ,\eta )=-\Phi (\zeta ;-\xi ,-\eta ) \end{array}$$

(202) $$\begin{array}{cc} & =-\phi \left(\zeta \right)-\frac{\xi -\eta }{2}\log\zeta +\frac{\xi }{2}\log\left(\left(\zeta -\alpha c\right)\left(\zeta -\alpha c^{-1}\right)\right)-\frac{\eta }{2}\log\left(\left(\zeta -c\right)\left(\zeta -c^{-1}\right)\right), \end{array}$$

where we have used (142) and (179) to write (200).

In the formulas that will be used in Section 11, Φ and Ψ will always appear in the form

$$\begin{array}{c} e^{\pm 2N\Phi (\zeta ;\xi_{N},\eta_{N})},\;e^{\pm 2N\Psi (\zeta ;\xi_{N},\eta_{N})},\;\;\text{with}\;\;\xi_{N}=\frac{x}{N}-1,\;\eta_{N}=\frac{y}{N}-1, \end{array}$$

for certain integers x,y∈{1,…,2N−1}. Because *x* and *y* are integers, the functions $\zeta \mapsto e^{\pm 2N\Phi (\zeta ;\xi_{N},\eta_{N})}$ and $\zeta \mapsto e^{\pm 2N\Psi (\zeta ;\xi_{N},\eta_{N})}$ have no jumps along $\left(-\infty ,c^{-1}\right]\cup \left\{c^{-1}+R_{1}e^{it}:-\pi \leq t\leq -\theta_{1}\right\}$. Also, for any (ξ,η)∈H, ReΦ and ReΨ are harmonic on $C\setminus (\Sigma_{1}\cup \left\{0,\alpha c,\alpha c^{-1},c,c^{-1}\right\})$, and well defined and continuous on $C\setminus \{0,\alpha c,\alpha c^{-1},c,c^{-1}\}$. For $\left(\xi ,\eta \right)\in H^{o}$, we note the following basic properties of Φ:

(203a) $$\begin{array}{ccc} \Phi (\zeta ) & =-\frac{1+\xi -\eta }{2}\log\zeta +O\left(1\right)\;\text{as}\;\zeta \to 0, & \lim_{\zeta \to 0}\text{Re}\;\Phi \left(\zeta \right)=+\infty , \end{array}$$

(203b) $$\begin{array}{ccc} \Phi (\zeta ) & =\frac{1+\xi }{2}\log\left(\zeta -\alpha c\right)+O\left(1\right)\;\text{as}\;\zeta \to \alpha c, & \lim_{\zeta \to \alpha c}\text{Re}\;\Phi \left(\zeta \right)=-\infty , \end{array}$$

(203c) $$\begin{array}{ccc} \Phi (\zeta ) & =\frac{1+\xi }{2}\log\left(\zeta -\alpha c^{-1}\right)+O\left(1\right)\;\text{as}\;\zeta \to \alpha c^{-1}, & \lim_{\zeta \to \alpha c^{-1}}\text{Re}\;\Phi \left(\zeta \right)=-\infty , \end{array}$$

(203d) $$\begin{array}{ccc} \Phi (\zeta ) & =-\frac{1+\eta }{2}\log\left(\zeta -c\right)+O\left(1\right)\;\text{as}\;\zeta \to c, & \lim_{\zeta \to c}\text{Re}\;\Phi \left(\zeta \right)=+\infty , \end{array}$$

(203e) $$\begin{array}{ccc} \Phi (\zeta ) & =-\frac{1+\eta }{2}\log\left(\zeta -c^{-1}\right)+O\left(1\right)\;\text{as}\;\zeta \to c^{-1}, & \lim_{\zeta \to c^{-1}}\text{Re}\;\Phi \left(\zeta \right)=+\infty , \end{array}$$

(203f) $$\begin{array}{ccc} \Phi (\zeta ) & =\frac{1-\xi +\eta }{2}\log\left(\zeta \right)+O\left(1\right)\;\text{as}\;\zeta \to \infty , & \lim_{\zeta \to \infty }\text{Re}\;\Phi \left(\zeta \right)=+\infty , \end{array}$$

and similarly

(204a) $$\begin{array}{cc} & \lim_{\zeta \to 0}\text{Re}\;\Psi \left(\zeta \right)=\lim_{\zeta \to c}\text{Re}\;\Psi \left(\zeta \right)=\lim_{\zeta \to c^{-1}}\text{Re}\;\Psi \left(\zeta \right)=\lim_{\zeta \to \infty }\text{Re}\;\Psi \left(\zeta \right)=-\infty , \end{array}$$

(204b) $$\begin{array}{cc} & \lim_{\zeta \to \alpha c}\text{Re}\;\Psi \left(\zeta \right)=\lim_{\zeta \to \alpha c^{-1}}\text{Re}\;\Psi \left(\zeta \right)=+\infty . \end{array}$$

Because the saddle points are the solutions to (41), it follows from (163), (200), and (202) that they are also the zeros of $\Phi^{\prime }$ and $\Psi^{\prime }$. For the saddle point analysis, it will be important to know: (1) the sign of $|s-c^{-1}|-R_{1}$ and (2) whether $\Phi^{\prime }\left(s\right)=0$ or $\Psi^{\prime }\left(s\right)=0$. We summarize the different cases in the next lemma.

Lemma 10 Let $\left(\xi ,\eta \right)\in L_{\alpha }$ and s=s(ξ,η;α). Then, we have

(a) $\Phi^{\prime }\left(s\right)=0$ and $|s-c^{-1}|<R_{1}$ if and only if ξ<0 and $\eta <\frac{\xi }{2}$,
(b) $\Phi^{\prime }\left(s\right)=0$ and $|s-c^{-1}|>R_{1}$ if and only if ξ<0 and $\eta >\frac{\xi }{2}$,
(c) $\Psi^{\prime }\left(s\right)=0$ and $|s-c^{-1}|<R_{1}$ if and only if ξ>0 and $\eta >\frac{\xi }{2}$,
(d) $\Psi^{\prime }\left(s\right)=0$ and $|s-c^{-1}|>R_{1}$ if and only if ξ>0 and $\eta <\frac{\xi }{2}$,
(e) $|s-c^{-1}|=R_{1}$ if and only if ξ=0 or $\eta =\frac{\xi }{2}$.

This is an immediate consequence of Propositions 4 and 5.▪

### 10.2 The level set $N_{\Phi }$

**FIGURE 12:**
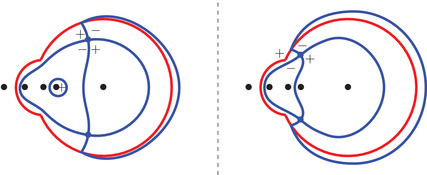
The set $N_{\Phi }$ is represented in blue, and $\Sigma_{\alpha }\cup \Sigma_{1}$ in red. The parameters are (α,ξ,η)=(0.4,−0.12,−0.86) (left) and (α,ξ,η)=(0.4,−0.22,−0.66) (right), and they satisfy $\eta <\frac{\xi }{2}<0$. The sign of Re(Φ(ζ)−Φ(s)) in the different regions delimited by $N_{\Phi }$ is indicated with ±. In each figure, the black dots represent 0, αc, $\alpha c^{-1}$, *c*, and $c^{-1}$ and the blue dots are *s* and $\bar{s}$

**FIGURE 13:**
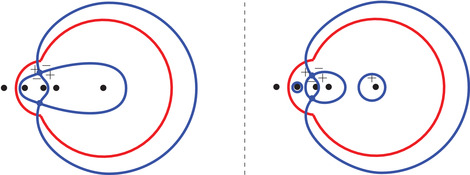
The set $N_{\Phi }$ is represented in blue, and $\Sigma_{\alpha }\cup \Sigma_{1}$ in red. The parameters are (α,ξ,η)=(0.4,−0.55,−0.414) (left) and (α,ξ,η)=(0.4,−0.88,−0.502) (right), and they satisfy $\eta <\frac{\xi }{2}<0$. The sign of Re(Φ(ζ)−Φ(s)) in the different regions delimited by $N_{\Phi }$ is indicated with ±. In each figure, the black dots represent 0, αc, $\alpha c^{-1}$, *c*, and $c^{-1}$ and the blue dots are *s* and $\bar{s}$

**FIGURE 14:**
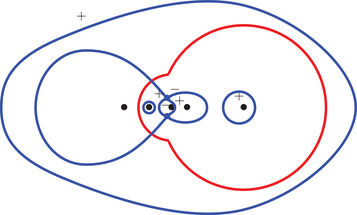
The set $N_{\Phi }$ is represented in blue, and $\Sigma_{\alpha }\cup \Sigma_{1}$ in red. The parameters are (α,ξ,η)=(0.4,−0.943,−0.538), and they satisfy $\eta <\frac{\xi }{2}<0$. The sign of Re(Φ(ζ)−Φ(s)) in the different regions delimited by $N_{\Phi }$ is indicated with ±. The black dots represent 0, αc, $\alpha c^{-1}$, *c*, and $c^{-1}$ and the blue dots are *s* and $\bar{s}$

We study the set

$$\begin{array}{c} N_{\Phi }=\left\{z\in C:\text{Re}\;\Phi \left(z\right)=\text{Re}\;\Phi \left(s\right)\right\}, \end{array}$$

in case $\eta \leq \frac{\xi }{2}<0$. We have represented $N_{\Phi }$ for different values of (α,ξ,η) in Figures 12, 13, and 14. There are in total eight saddles, which are the zeros of $\Phi^{\prime }$ and $\Psi^{\prime }$. From (203) and (204), both $\Phi^{\prime }$ and $\Psi^{\prime }$ vanish at least once on each of the intervals (−∞,0), $\left(\alpha c,\alpha c^{-1}\right)$, and $\left(c,c^{-1}\right)$. This determines the location of six saddles. The remaining two are *s* and $\bar{s}$, and we already know from Lemma 10(a) and (e) that $\Phi^{\prime }\left(s\right)=0=\Phi^{\prime }\left(\bar{s}\right)$. Therefore, $\Phi^{\prime }\neq 0$ on $\left(0,\alpha c\right)\cup \left(\alpha c^{-1},c\right)$. Because $\Phi^{\prime }\left(\zeta \right)\in R$ for $\zeta \in R\setminus \{0,\alpha c,\alpha c^{-1},c,c^{-1}\}$, this implies by (203) that $N_{\Phi }$ intersects exactly once each of these two intervals.

We show with the next two lemmas that the set $N_{\Phi }\cap \left(\bar{\Sigma_{\alpha }\cup \Sigma_{1}}\right)\cap C^{+}$ is either the empty set or a singleton.

For $\zeta \in C\setminus \{0,\alpha c,\alpha c^{-1},c,c^{-1}\}$, we define the following functions:

$$\begin{array}{c} f_{1}\left(\zeta \right)=\log\frac{\left(\zeta -c\right)\left(\zeta -c^{-1}\right)}{\zeta },\;f_{2}\left(\zeta \right)=\log\frac{\zeta }{\left(\zeta -\alpha c\right)\left(\zeta -\alpha c^{-1}\right)},\;f_{3}\left(\zeta \right)=\log\frac{\left(\zeta -c\right)\left(\zeta -c^{-1}\right)}{\left(\zeta -\alpha c\right)\left(\zeta -\alpha c^{-1}\right)}. \end{array}$$

Lemma 11 If ζ moves along $\left(\bar{\Sigma_{\alpha }\cup \Sigma_{1}}\right)\cap C^{+}$ from left to right, then

(1) $\text{Re}\;f_{1}$ is strictly decreasing on $\Sigma_{\alpha }\cap C^{+}$ and constant on $\Sigma_{1}\cap C^{+}$,
(2) $\text{Re}\;f_{2}$ is constant on $\Sigma_{\alpha }\cap C^{+}$ and strictly decreasing on $\Sigma_{1}\cap C^{+}$,
(3) $\text{Re}\;f_{3}$ is strictly decreasing.

A long and tedious computation shows that $\frac{d}{dt}\text{Re}\;f_{1}\left(\alpha c^{-1}+R_{\alpha }e^{-it}\right)$ has the same sign as sint. In particular, $\text{Re}\;f_{1}\left(\zeta \right)$ is strictly decreasing along $\Sigma_{\alpha }\cap C^{+}$ as ζ moves from left to right. Another (and simpler) computation gives

$$\begin{array}{c} \frac{d}{dt}f_{1}\left(c^{-1}+R_{1}e^{-it}\right)=-i\frac{\cos t+\frac{\sqrt{1-\alpha +\alpha^{2}}}{1-\alpha }}{\cos t+\frac{2-3\alpha +2\alpha^{2}}{2\left(1-\alpha \right)\sqrt{1-\alpha +\alpha^{2}}}}. \end{array}$$

This expression is purely imaginary, so $\text{Re}\;f_{1}$ is constant on Σ_1_. The proofs for *f*_2_ and *f*_3_ are similar, so we omit them.▪

Corollary 3 For $\eta \leq \frac{\xi }{2}<0$, the function ζ↦ReΦ(ζ) is strictly decreasing as ζ moves along $\left(\bar{\Sigma_{\alpha }\cup \Sigma_{1}}\right)\cap C^{+}$ from left to right.

We know from Lemma 9 that Reϕ=0 on $\Sigma_{\alpha }\cup \Sigma_{1}$. Therefore, from the expression (200) for Φ, for $\zeta \in \Sigma_{\alpha }\cup \Sigma_{1}$ we have

(205) $$\begin{array}{cc} \text{Re}\;\Phi (\zeta ) & =-\frac{\xi -\eta }{2}\log\left|\zeta \right|+\frac{\xi }{2}\log\left|\left(\zeta -\alpha c\right)\left(\zeta -\alpha c^{-1}\right)\right|-\frac{\eta }{2}\log\left|\left(\zeta -c\right)\left(\zeta -c^{-1}\right)\right|\\ & =\left(\frac{\xi }{4}-\frac{\eta }{2}\right)\text{Re}\;f_{1}\left(\zeta \right)-\frac{\xi }{4}\left(\text{Re}\;f_{2}\left(\zeta \right)+\text{Re}\;f_{3}\left(\zeta \right)\right). \end{array}$$

The claim follows from Lemma 11, because ξ<0 and $\frac{\xi }{2}-\eta \geq 0$.▪

##### 10.2.1 Notation

For a given closed curve σ, we denote int(σ) for the open and bounded region delimited by σ.

Because $\Phi^{\prime }\left(s\right)=0$, there are four curves ${\left\{\Gamma_{j}\right\}}_{j=1}^{4}$ emanating from *s* that belongs to $N_{\Phi }$. By Corollary 3, $N_{\Phi }\cap \left(\bar{\Sigma_{\alpha }\cup \Sigma_{1}}\right)\cap C^{+}$ is either the empty set or a singleton, so at least three of the $\Gamma_{j}$'s, say $\Gamma_{1},\Gamma_{2},\Gamma_{3}$, do not intersect $\left(\bar{\Sigma_{\alpha }\cup \Sigma_{1}}\right)\cap C^{+}$. The curves $\Gamma_{j}$, j=1,2,3 cannot lie entirely in $C^{+}$; otherwise the max/min principle for harmonic functions would imply that ReΦ is constant within the region $\text{int}(\Gamma_{j})$. Therefore, $\Gamma_{j}$, j=1,2,3 have to intersect R. Note that $\bar{\Phi (\zeta )}=\Phi \left(\bar{\zeta }\right)$ implies that $N_{\Phi }$ is symmetric with respect to R. In particular, the curves $\Gamma_{j}$, j=1,2,3 join *s* with $\bar{s}$. The next lemma states that Γ_4_ is not contained in the region $\text{int}(\bar{\Sigma_{\alpha }\cup \Sigma_{1}})$.

Lemma 12 $N_{\Phi }\cap \left(\bar{\Sigma_{\alpha }\cup \Sigma_{1}}\right)\cap C^{+}$ is a singleton.

Assume on the contrary that Γ_4_ lies entirely in $\text{int}(\bar{\Sigma_{\alpha }\cup \Sigma_{1}})$, and denote $p_{j}$ for the intersection point of $\Gamma_{j}$ with R. We assume without loss of generality that $p_{1}<p_{2}<p_{3}<p_{4}$. There is at most one $p_{j}$ inside each of the intervals

$$\begin{array}{c} \left(\alpha c^{-1}-R_{\alpha },\alpha c\right),\;\left(\alpha c,\alpha c^{-1}\right),\;\left(\alpha c^{-1},c\right),\;\left(c,c^{-1}\right),\;\left(c^{-1},c^{-1}+R_{1}\right), \end{array}$$

otherwise we again find a contradiction using the max/min principle for harmonic functions. Thus, there are five five possibilities for the location of the $p_{j}$'s, and each of them leads to a contradiction. Let us treat the case

(206) $$\begin{array}{c} p_{1}\in \left(\alpha c,\alpha c^{-1}\right),\;p_{2}\in \left(\alpha c^{-1},c\right),\;p_{3}\in \left(c,c^{-1}\right),\;p_{4}\in \left(c^{-1},c^{-1}+R_{1}\right). \end{array}$$

Because Re(Φ(ζ)−Φ(s)) changes sign as ζ crosses $N_{\Phi }\setminus \left\{s,\bar{s}\right\}$, by (203) we must have

(207) $$\begin{array}{c} N_{\Phi }=\sigma_{1}\cup \sigma_{2}\cup \overset{4}{\underset{j=1}{⋃}}\Gamma_{j}, \end{array}$$

where σ_1_ is a closed curve surrounding either αc or $\alpha c^{-1}$, such that $\text{int}\left(\sigma_{1}\right)\cap N_{\Phi }=\emptyset$, and σ_2_ is a closed curve surrounding either *c* or $c^{-1}$, such that $\text{int}\left(\sigma_{2}\right)\cap N_{\Phi }=\emptyset$. Because $N_{\Phi }$ intersects both (0,αc) and $\left(\alpha c^{-1},c\right)$ exactly once, σ_1_ surrounds αc and σ_2_ surrounds $c^{-1}$. Then, the max/min principle implies that ReΦ is constant on $\text{int}\left(\bar{\Gamma_{3}\cup \Gamma_{4}}\right)\setminus \text{int}\left(\sigma_{2}\right)$, which is a contradiction. The four other cases than (206) can be treated similarly, so we omit the proofs.▪

**FIGURE 15:**
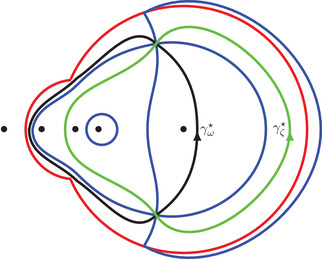
The set $N_{\Phi }$ is represented in blue, and $\Sigma_{\alpha }\cup \Sigma_{1}$ in red. The parameters are (α,ξ,η)=(0.4,−0.12,−0.86) as in Figure 12 (left). The contour $\gamma_{\zeta }^{*}$ is represented in green, and $\gamma_{\omega }^{*}$ in black. The black dots represent 0, αc, $\alpha c^{-1}$, *c*, and $c^{-1}$ and the blue dots are *s* and $\bar{s}$

**FIGURE 16:**
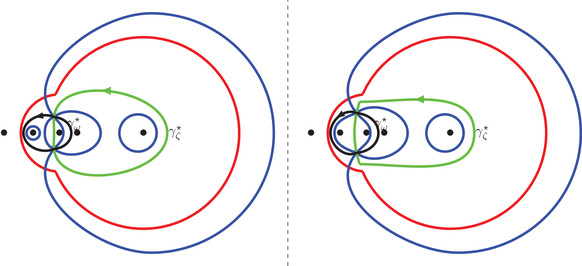
The set $N_{\Phi }$ is represented in blue, and $\Sigma_{\alpha }\cup \Sigma_{1}$ in red. The parameters are (α,ξ,η)=(0.4,−0.88,−0.502) (left) and (α,ξ,η)=(0.4,−0.7,−0.35) (right). The contour $\gamma_{\zeta }^{*}$ is represented in green, and $\gamma_{\omega }^{*}$ in black. The black dots represent 0, αc, $\alpha c^{-1}$, *c*, and $c^{-1}$ and the blue dots are *s* and $\bar{s}$

Lemma 12 states that Γ_4_ crosses $\Sigma_{\alpha }\cup \Sigma_{1}$ exactly once. We know from (203) that ReΦ(ζ)→+∞ as ζ→∞, so Γ_4_ intersects the real line, and then by symmetry ends at $\bar{s}$. So each of the $\Gamma_{j}$'s intersects R. We denote $p_{j}$ for the intersection point of $\Gamma_{j}$ with R, and choose the ordering such that $p_{1}<p_{2}<p_{3}$. We recall that Re(Φ(ζ)−Φ(s)) is harmonic for $\zeta \in C\setminus (\Sigma_{1}\cup \left\{0,\alpha c,\alpha c^{-1},c,c^{-1}\right\})$ and changes sign as ζ crosses $N_{\Phi }\setminus \left\{s,\bar{s}\right\}$. Therefore, by (203), the region $\text{int}(\bar{\Gamma_{1}\cup \Gamma_{2}})$ must contain at least one of the singularities αc and $\alpha c^{-1}$, and $\text{int}(\bar{\Gamma_{2}\cup \Gamma_{3}})$ must contain at least one of the singularities *c* and $c^{-1}$. There are still quite a few cases that can occur. The figures provide a fairly good overview (though not complete) of what can happen:

1. In Figure 12 (left), $\alpha c,\alpha c^{-1},c\in \text{int}\left(\bar{\Gamma_{1}\cup \Gamma_{2}}\right)$, $c^{-1}\in \text{int}\left(\bar{\Gamma_{2}\cup \Gamma_{3}}\right)$.
2. In Figures 12 (right) and 13 (left), $\alpha c,\alpha c^{-1}\in \text{int}\left(\bar{\Gamma_{1}\cup \Gamma_{2}}\right)$ and $c,c^{-1}\in \text{int}\left(\bar{\Gamma_{2}\cup \Gamma_{3}}\right)$.
3. In Figures 13 (right) and 14, $\alpha c^{-1}\in \text{int}\left(\bar{\Gamma_{1}\cup \Gamma_{2}}\right)$ and $c\in \text{int}(\bar{\Gamma_{2}\cup \Gamma_{3}})$.

Furthermore, Γ_4_ intersects both Σ_1_ and $\left(c^{-1}+R_{1},+\infty \right)$ in Figure 12, intersects both $\Sigma_{\alpha }$ and $\left(c^{-1}+R_{1},+\infty \right)$ in Figure 13, and intersects both $\Sigma_{\alpha }$ and $\left(-\infty ,\alpha c^{-1}-R_{\alpha }\right)$ in Figure 14. There are also some obvious intermediate cases, which are not illustrated by a figure. In all cases, we can find contours $\gamma_{\zeta }^{*}$ and $\gamma_{\omega }^{*}$ as described in the following proposition. These contours are illustrated for two different situations in Figures 15 and 16 (left).

Proposition 16 Let $\left(\xi ,\eta \right)\in L_{\alpha }$ with $\eta <\frac{\xi }{2}<0$. There exist contours $\gamma_{\zeta }^{*}$ and $\gamma_{\omega }^{*}$ such that

- $\gamma_{\omega }^{*}\subset \text{int}\left(\bar{\Sigma_{\alpha }\cup \Sigma_{1}}\right)$, it surrounds αc and $\alpha c^{-1}$, and it goes through *s* and $\bar{s}$ in such a way that
  $$\begin{array}{c} \text{Re}\;\Phi \left(\omega \right)>\text{Re}\;\Phi \left(s\right),\;\omega \in \gamma_{\omega }^{*}\setminus \left\{s,\bar{s}\right\}, \end{array}$$
- $\gamma_{\zeta }^{*}\subset \text{int}\left(\gamma_{1}\right)$, surrounds *c* and $c^{-1}$, and it goes through *s* and $\bar{s}$ in such a way that
  $$\begin{array}{c} \text{Re}\;\Phi \left(\zeta \right)<\text{Re}\;\Phi \left(s\right),\;\zeta \in \gamma_{\zeta }^{*}\setminus \left\{s,\bar{s}\right\}. \end{array}$$

If $\eta =\frac{\xi }{2}$, we know from Proposition 4 (b) that *s* lies on $\gamma_{1}\setminus \bar{\Sigma_{1}}$. For the saddle point analysis, we will need $\gamma_{\zeta }^{*}$ lying inside γ_1_ (not necessarily strictly inside). To prove existence of such a contour $\gamma_{\zeta }^{*}$, we need to know that ReΦ(ζ)−ReΦ(s) is strictly negative for $\zeta \in \gamma_{1}\setminus \bar{\Sigma_{1}}$ (at least in small neighborhoods of *s* and $\bar{s}$).

Lemma 13 Let $\eta =\frac{\xi }{2}<0$. For $\zeta \in \gamma_{1}\setminus \left(\bar{\Sigma_{1}}\cup \left\{s\right\}\right)\cap C^{+}$, we have ReΦ(ζ)<ReΦ(s).

Let $\zeta =c^{-1}+R_{1}e^{it}$. For $t\in (\theta_{1},\pi )$, we have

(208) $$\begin{array}{c} \text{Re}\;\left(\Phi^{\prime }\left(\zeta \right)d\zeta \right)=\frac{-\cos\left(\frac{t}{2}\right)\left(\sqrt{\cos\theta_{1}-\cos t}\left(\cos t+a_{1}\right)+\frac{\xi }{2}\left(\cos t+a_{2}\right)\sqrt{1-\cos t}\right)}{\sqrt{2}\left(\cos t+\frac{2-\alpha +\alpha^{2}}{2\sqrt{1-\alpha +\alpha^{2}}}\right)\left(\cos t+\frac{2-3\alpha +2\alpha^{2}}{2\left(1-\alpha \right)\sqrt{1-\alpha +\alpha^{2}}}\right)}, \end{array}$$

where $a_{1},a_{2}$ are given by $a_{1}=\frac{\alpha^{2}+\left(1-\alpha \right)\sqrt{1-\alpha +\alpha^{2}}}{2(1-\alpha )}$ and $a_{2}=\frac{2-3\alpha +2\alpha^{2}+\alpha^{3}}{2\left(1-\alpha \right)\sqrt{1-\alpha +\alpha^{2}}}$ and satisfy $a_{1}>a_{2}>1$. The expression (208) vanishes if and only if

(209) $$\begin{array}{c} \frac{\sqrt{\cos\theta_{1}-\cos t}}{\sqrt{1-\cos t}}=-\frac{\xi }{2}\frac{\cos t+a_{2}}{\cos t+a_{1}}. \end{array}$$

Because the left‐hand side is strictly decreasing, and the right‐hand‐side is strictly increasing as *t* decreases from π to θ_1_, there is a unique $\zeta =c^{-1}+R_{1}e^{it}$, $t\in (\theta_{1},\pi )$, such that $\text{Re}\;(\Phi^{\prime }\left(\zeta \right)d\zeta )=0$, and this must be *s*. This implies that ReΦ(ζ)−ReΦ(s) is of constant sign on $\gamma_{1}\setminus \left(\bar{\Sigma_{1}}\cup \left\{s\right\}\right)\cap C^{+}$. By (208), $\text{Re}\;(\Phi^{\prime }\left(\zeta \right)d\zeta )>0$ at $t=\theta_{1}$ (recall that ξ<0), so the claim is proved.▪

Therefore, we can find contours $\gamma_{\zeta }^{*}$ and $\gamma_{\omega }^{*}$ as described in Proposition 17, see also Figure 16 (right).

Proposition 17 Let $\left(\xi ,\eta \right)\in L_{\alpha }$ with $\eta =\frac{\xi }{2}<0$. There exist contours $\gamma_{\zeta }^{*}$ and $\gamma_{\omega }^{*}$ such that

- $\gamma_{\omega }^{*}\subset \text{int}\left(\bar{\Sigma_{\alpha }\cup \Sigma_{1}}\right)$, it surrounds αc and $\alpha c^{-1}$, and it goes through *s* and $\bar{s}$ in such a way that
  $$\begin{array}{c} \text{Re}\;\Phi \left(\omega \right)>\text{Re}\;\Phi \left(s\right),\;\omega \in \gamma_{\omega }^{*}\setminus \left\{s,\bar{s}\right\}, \end{array}$$
- $\gamma_{\zeta }^{*}\subset \bar{\text{int}(\gamma_{1})}$, surrounds *c* and $c^{-1}$, and it goes through *s* and $\bar{s}$ in such a way that
  $$\begin{array}{c} \text{Re}\;\Phi \left(\zeta \right)<\text{Re}\;\Phi \left(s\right),\;\zeta \in \gamma_{\zeta }^{*}\setminus \left\{s,\bar{s}\right\}. \end{array}$$

## 11 SADDLE POINT ANALYSIS

In this section, we prove Proposition 11 by means of a saddle point analysis that mainly follows the lines of Ref. 21. This analysis relies mostly on Sections 9 and 10 and is only valid for (ξ,η) in the lower left part of the liquid region, that is for $\left(\xi ,\eta \right)\in L_{\alpha }\cap \left\{\eta \leq \frac{\xi }{2}\leq 0\right\}$. We divide the proof in three subcases: $\eta \leq \frac{\xi }{2}<0$, $\eta <\frac{\xi }{2}=0$, and η=ξ=0.

Remark 6 By adapting the analysis of this section and of Section 10, it is possible to carry out similar saddle point analysis when (ξ,η) lies in the other quadrants of the liquid region. Note however that this is not needed, thanks to the symmetries of Section 7.2 (see also Proposition 11).

### 11.1 The case $\eta \leq \frac{\xi }{2}<0$

The double integral I is defined in (116). The associated two contours of integration can be chosen freely, as long as they are closed curves surrounding *c* and $c^{-1}$ once in the positive direction, and not surrounding 0. From now, it will be convenient to take different contours in the ζ and ω variables, so we indicate this in the notation by rewriting (116) as

(210) $$\begin{array}{c} I\left(x,y;H\right)=\frac{1}{{\left(2\pi i\right)}^{2}}\int_{\gamma_{\zeta }}d\zeta \int_{\gamma_{\omega }}d\omega H\left(\omega ,\zeta \right)W\left(\omega \right)R^{U}\left(\omega ,\zeta \right)\frac{\omega^{N}}{\zeta^{N}}q{\left(\omega ,\zeta \right)}^{y}\tilde{q}{\left(\omega ,\zeta \right)}^{x}. \end{array}$$

Only the first column of *U* appears in (210), which is independent of the choice of the contour $\gamma_{C}$ associated to the RH problem for *U*. However, by using the jumps for *U*, we will find (just below) another formula for I in terms of the second column of *U*. Therefore, the choice of $\gamma_{C}$ will matter. To be able to use the steepest descent of Section 9, we assume from now that $\gamma_{C}=\gamma_{1}$. Recall that *T* is expressed in terms of *U* via (183), and define

(211) $$\begin{array}{c} {\tilde{R}}^{T}\left(\omega ,\zeta \right)=\left(\begin{array}{cc} 1 & 0 \end{array}\right)T^{-1}\left(\omega \right)T\left(\zeta \right)\left(\begin{array}{c} 1\\ 0 \end{array}\right). \end{array}$$

By Proposition 15, ${\tilde{R}}^{T}\left(\omega ,\zeta \right)$ is uniformly bounded as ζ and ω stay bounded away from $r_{+}$ and $r_{-}$. We will need the analytic continuation in ω of ${\tilde{R}}^{T}\left(\omega ,\zeta \right)$ from the interior of γ_1_ to the bounded region delimited by $\bar{\Sigma_{1}\cup \Sigma_{\alpha }}$ (see Figure 10). We denote it ${\tilde{R}}^{T,a}\left(\omega ,\zeta \right)$, and by (185) it is given by

(212) $${\tilde{R}}^{T,a}\left(\omega ,\zeta \right)=\left\{\begin{array}{cc} \left(1\;0\right)T^{-1}\left(\omega \right)T\left(\zeta \right)\left(\begin{array}{c} 1\\ 0 \end{array}\right), & |\omega -c^{-1}|<R_{1},\;\zeta \in C\setminus \gamma_{1},\\ \left(1\;-e^{4N\phi (\omega )}\right)T^{-1}\left(\omega \right)T\left(\zeta \right)\left(\begin{array}{c} 1\\ 0 \end{array}\right), & \omega \in \text{int}\left(\left(\gamma_{1}\setminus \Sigma_{1}\right)\cup \Sigma_{\alpha }\right),\;\zeta \in C\setminus \gamma_{1}. \end{array}\right.$$

By Lemma 9, Reϕ(ω)<0 for $\omega \in \text{int}(\left(\gamma_{1}\setminus \Sigma_{1}\right)\cup \Sigma_{\alpha })$, so ${\tilde{R}}^{T,a}\left(\omega ,\zeta \right)$ remains bounded as N→+∞, uniformly for ζ and ω bounded away from $r_{+}$ and $r_{-}$, as long as $\omega \in \text{int}(\bar{\Sigma_{1}}\cup \Sigma_{\alpha })$. Our next goal is to prove the following.

Proposition 18 Let (x,y) be coordinates inside the hexagon, such that $\xi :=\frac{x}{N}-1$ and $\eta :=\frac{y}{N}-1$ satisfy $\left(\xi ,\eta \right)\in L_{\alpha }$ with $\eta \leq \frac{\xi }{2}<0$. Take $\gamma_{\zeta }^{*}$ and $\gamma_{\omega }^{*}$ as in Proposition 16 if $\eta <\frac{\xi }{2}$, and as in Proposition 17 if $\eta =\frac{\xi }{2}$ (see also Figures 15 and 16). Then, the double contour integral (116) is equal to

(213) $$I\left(x,y;H\right)=\frac{1}{2\pi i}\int_{\bar{s}}^{s}H\left(\zeta ,\zeta \right)d\zeta +\frac{1}{{\left(2\pi i\right)}^{2}}\int_{\gamma_{\zeta }^{*}}d\zeta \int_{\gamma_{\omega }^{*}}\frac{d\omega }{\omega -\zeta }H\left(\omega ,\zeta \right){\tilde{R}}^{T,a}\left(\omega ,\zeta \right)e^{2N(\Phi (\zeta ;\xi ,\eta )-\Phi (\omega ;\xi ,\eta ))}.$$

Remark 7 By Proposition 17, $\gamma_{\zeta }^{*}$ intersects $\gamma_{1}\setminus \bar{\Sigma_{1}}$ whenever $\eta =\frac{\xi }{2}$. We do not indicate whether we take the + or − boundary values in the integrand of (213). This is without ambiguity, because

$$\begin{array}{c} \zeta \mapsto T\left(\zeta \right)\left(\begin{array}{c} 1\\ 0 \end{array}\right)e^{2N\Phi (\zeta ;\xi ,\eta )} \end{array}$$

has no jumps on γ_1_ (this can be verified using (184) and (185)).

Take $\gamma_{\omega }=\gamma_{1}$ and $\gamma_{\zeta }$ lying strictly inside γ_1_ in (210). From the jumps for *U* (25), we have

$$\begin{array}{c} W\left(\omega \right)\left(\begin{array}{cc} 0 & 1 \end{array}\right)U{\left(\omega \right)}^{-1}=\left(\begin{array}{cc} 1 & 0 \end{array}\right)U_{-}{\left(\omega \right)}^{-1}-\left(\begin{array}{cc} 1 & 0 \end{array}\right)U_{+}{\left(\omega \right)}^{-1},\;\omega \in \gamma_{1}. \end{array}$$

Inserting this in (210), and using the U↦T transformation (183), we get

(214) $$\begin{array}{cc} I(x,y;H) & =\frac{1}{{\left(2\pi i\right)}^{2}}\int_{\gamma_{\zeta }}d\zeta \int_{\gamma_{\omega }=\gamma_{1}}\frac{d\omega }{\omega -\zeta }H\left(\omega ,\zeta \right){\tilde{R}}_{+}^{T}\left(\omega ,\zeta \right)e^{2N(g\left(\zeta \right)-g_{+}\left(\omega \right))}\frac{\omega^{N}}{\zeta^{N}}q{\left(\omega ,\zeta \right)}^{y}\tilde{q}{\left(\omega ,\zeta \right)}^{x}\\ & -\frac{1}{{\left(2\pi i\right)}^{2}}\int_{\gamma_{\zeta }}d\zeta \int_{\gamma_{\omega }=\gamma_{1}}\frac{d\omega }{\omega -\zeta }H\left(\omega ,\zeta \right){\tilde{R}}_{-}^{T}\left(\omega ,\zeta \right)e^{2N(g\left(\zeta \right)-g_{-}\left(\omega \right))}\frac{\omega^{N}}{\zeta^{N}}q{\left(\omega ,\zeta \right)}^{y}\tilde{q}{\left(\omega ,\zeta \right)}^{x}, \end{array}$$

where ${\tilde{R}}_{+}^{T}\left(\omega ,\zeta \right)$ and ${\tilde{R}}_{-}^{T}\left(\omega ,\zeta \right)$ denote the limits of ${\tilde{R}}^{T}\left(\omega^{\prime },\zeta \right)$ as $\omega^{\prime }\to \omega$ from the interior and exterior of γ_1_, respectively.

Remark 8 For x,y∈{1,2,…,2N−1}, we define

(215) $$\begin{array}{c} m\left(\omega ,\zeta \right)=\frac{1}{\omega -\zeta }H\left(\omega ,\zeta \right){\tilde{R}}^{T}\left(\omega ,\zeta \right)e^{2N(g(\zeta )-g(\omega ))}\frac{\omega^{N}}{\zeta^{N}}q{\left(\omega ,\zeta \right)}^{y}\tilde{q}{\left(\omega ,\zeta \right)}^{x}. \end{array}$$

The boundary values of *m* appear in the integrand of (214). We recall that *q* and $\tilde{q}$ are defined in (99), that *H* satisfies the conditions stated in Proposition 11, and that g(ω) is bounded for ω in compact subsets and satisfies g(ω)∼log(ω) as ω→∞. Therefore, the following properties hold:

(i) The function ζ↦m(ω,ζ) is analytic in $C\setminus \{\omega ,0,c,c^{-1}\}$,
(ii) The function ω↦m(ω,ζ) is analytic in $\left(C\cup \left\{\infty \right\}\right)\setminus \left(\left\{\zeta ,\alpha c,\alpha c^{-1}\right\}\cup \gamma_{1}\right)$.

The statement that ω↦m(ω,ζ) is analytic at ∞ deserves a little computation: because x,y∈{1,2,…,2N−1}, we have $m\left(\omega ,\zeta \right)=O\left(\omega^{-1-2N+N-y+x}\right)=O\left(\omega^{-2}\right)$ as ω→∞.

If $\eta <\frac{\xi }{2}$, Proposition 16 states that $\gamma_{\zeta }$ lies strictly inside γ_1_, so in this case we can (and do) take $\gamma_{\zeta }=\gamma_{\zeta }^{*}$ in (214). If $\eta =\frac{\xi }{2}$, we know from Proposition 17 that $\gamma_{\zeta }^{*}$ intersects $\gamma_{1}\setminus \bar{\Sigma_{1}}$. In this case, we let $\gamma_{\zeta }$ in (214) tend to $\gamma_{\zeta }^{*}$ from the interior of γ_1_. In what follows, we will abuse notation and simply write $\gamma_{\zeta }^{*}$. We will also omit the boundary values in the ζ‐variable, see Remark 7 (or (i)).

Let us deform $\gamma_{\omega }$ from γ_1_ to $\Sigma_{1}\cup \bar{\Sigma_{\alpha }}$ in each of the two integrals of (214). For each deformation, we pick up a residue at ω=αc. These residues cancel each other and we get

$$\begin{array}{cc} I(x,y;H) & =\frac{1}{{\left(2\pi i\right)}^{2}}\int_{\gamma_{\zeta }^{*}}d\zeta \int_{\gamma_{\omega }=\Sigma_{1}\cup \bar{\Sigma_{\alpha }}}\frac{d\omega }{\omega -\zeta }H\left(\omega ,\zeta \right){\tilde{R}}_{+}^{T,a}\left(\omega ,\zeta \right)e^{2N(g\left(\zeta \right)-g_{+}\left(\omega \right))}\frac{\omega^{N}}{\zeta^{N}}q{\left(\omega ,\zeta \right)}^{y}\tilde{q}{\left(\omega ,\zeta \right)}^{x}\\ & -\frac{1}{{\left(2\pi i\right)}^{2}}\int_{\gamma_{\zeta }^{*}}d\zeta \int_{\gamma_{\omega }=\Sigma_{1}\cup \bar{\Sigma_{\alpha }}}\frac{d\omega }{\omega -\zeta }H\left(\omega ,\zeta \right){\tilde{R}}_{-}^{T}\left(\omega ,\zeta \right)e^{2N(g\left(\zeta \right)-g_{-}\left(\omega \right))}\frac{\omega^{N}}{\zeta^{N}}q{\left(\omega ,\zeta \right)}^{y}\tilde{q}{\left(\omega ,\zeta \right)}^{x}. \end{array}$$

By (ii), the integrand of the second integral has no poles in the exterior region of $\Sigma_{1}\cup \bar{\Sigma_{\alpha }}$, so by deforming $\gamma_{\omega }$ at ∞, we find that this integral is 0. Therefore, we simply get

$$\begin{array}{cc} I(x,y;H) & =\frac{1}{{\left(2\pi i\right)}^{2}}\int_{\gamma_{\zeta }^{*}}d\zeta \int_{\gamma_{\omega }=\Sigma_{1}\cup \bar{\Sigma_{\alpha }}}\frac{d\omega }{\omega -\zeta }H\left(\omega ,\zeta \right){\tilde{R}}_{+}^{T,a}\left(\omega ,\zeta \right)e^{2N(g\left(\zeta \right)-g_{+}\left(\omega \right))}\frac{\omega^{N}}{\zeta^{N}}q{\left(\omega ,\zeta \right)}^{y}\tilde{q}{\left(\omega ,\zeta \right)}^{x}. \end{array}$$

This formula can be written in terms of Φ (see Definition 7) as follows:

(216) $$\begin{array}{cc} I(x,y;H) & =\frac{1}{{\left(2\pi i\right)}^{2}}\int_{\gamma_{\zeta }^{*}}d\zeta \int_{\gamma_{\omega }=\Sigma_{1}\cup \bar{\Sigma_{\alpha }}}\frac{d\omega }{\omega -\zeta }H\left(\omega ,\zeta \right){\tilde{R}}_{+}^{T,a}\left(\omega ,\zeta \right)e^{2N(\Phi \left(\zeta ;\xi ,\eta \right)-\Phi_{+}\left(\omega ;\xi ,\eta \right))}, \end{array}$$

where ξ:=x/N−1 and η:=y/N−1. Finally, we deform $\gamma_{\omega }$ into $\gamma_{\omega }^{*}$. This gives the right‐most term of (213) plus a residue at ω=ζ (by (ii)). After a small computation, we find that this residue is the first term on the right‐hand side of (213). This finishes the proof.▪

Proof of Proposition 11 for $\eta \leq \frac{\xi }{2}<0$

Let $\{{\left(x_{N},y_{N}\right\}}_{N\geq 1}$ be a sequence satisfying (38) with $\left(\xi ,\eta \right)\in L_{\alpha }\cap \left\{\eta \leq \frac{\xi }{2}<0\right\}$, and define $\xi_{N}:=x_{N}/N-1$ and $\eta_{N}:=y_{N}/N-1$. By (38), we have $\xi_{N}\to \xi$ and $\eta_{N}\to \eta$ as N→+∞. If $\eta =\frac{\xi }{2}$, we assume that $\left(\xi_{N},\eta_{N}\right)\in L_{\alpha }\cap \left\{\eta \leq \frac{\xi }{2}<0\right\}$ for all large enough *N* (this is without loss of generality, see Lemma 7). Replacing (x,y) in (213) by $\left(x_{N},y_{N}\right)$, we get

(217) $$I\left(x_{N},y_{N};H\right)-\frac{1}{2\pi i}\int_{\bar{s_{N}}}^{s_{N}}H\left(\zeta ,\zeta \right)d\zeta =\frac{1}{{\left(2\pi i\right)}^{2}}\int_{\gamma_{\zeta }^{*}}d\zeta \int_{\gamma_{\omega }^{*}}\frac{d\omega }{\omega -\zeta }H\left(\omega ,\zeta \right){\tilde{R}}^{T,a}\left(\omega ,\zeta \right)e^{2N(\Phi_{N}\left(\zeta \right)-\Phi_{N}\left(\omega \right))},$$

where $s_{N}=s\left(\xi_{N},\eta_{N};\alpha \right)$, $\Phi_{N}\left(\zeta \right):=\Phi \left(\zeta ;\xi_{N},\eta_{N}\right)$, and the contours $\gamma_{\zeta }^{*}$ and $\gamma_{\omega }^{*}$ also depend on *N*, even though this is not indicated in the notation. Because $\gamma_{\zeta }^{*}$ and $\gamma_{\omega }^{*}$ do not pass through $r_{+}$ and $r_{-}$, Proposition 15 implies that

$$\begin{array}{c} {\tilde{R}}^{T,a}\left(\omega ,\zeta \right)=O\left(1\right),\;\text{as}\;N\to +\infty \;\;\text{uniformly}\;\text{for}\;\text{all}\;\zeta \in \gamma_{\zeta }^{*}\;\text{and}\;\omega \in \gamma_{\omega }^{*}. \end{array}$$

We also know from Propositions 16 and 17 that

$$\begin{array}{c} \text{Re}\;\Phi_{N}\left(\zeta \right)<\text{Re}\;\Phi_{N}\left(s_{N}\right)<\text{Re}\;\Phi_{N}\left(\omega \right),\;\text{for}\;\text{all}\;\zeta \in \gamma_{\zeta }^{*}\setminus \left\{s_{N},\bar{s_{N}}\right\},\omega \in \gamma_{\omega }^{*}\setminus \left\{s_{N},\bar{s_{N}}\right\}, \end{array}$$

which implies that the right‐hand side of (217) is

(218) $$\begin{array}{c} \frac{1}{{\left(2\pi i\right)}^{2}}\int_{\gamma_{\zeta }^{*}\cap D_{\varepsilon }}d\zeta \int_{\gamma_{\omega }^{*}\cap D_{\varepsilon }}\frac{d\omega }{\omega -\zeta }H\left(\omega ,\zeta \right){\tilde{R}}^{T,a}\left(\omega ,\zeta \right)e^{2N(\Phi_{N}\left(\zeta \right)-\Phi_{N}\left(\omega \right))}+O\left(e^{-C_{1}N}\right),\;\text{as}\;N\to \infty , \end{array}$$

for a certain $C_{1}>0$, and where $D_{\varepsilon }$ is the union of two small disks of radii ε>0 surrounding *s* and $\bar{s}$. Because $s_{N}$ and $\bar{s_{N}}$ are simple zeros of $\Phi_{N}^{\prime }$, we have the estimates

$$\begin{array}{cccc} & \text{Re}\;\left(\Phi_{N}\left(\zeta \right)-\Phi_{N}\left(s_{N}\right)\right)<-C_{2}{\left|\zeta -s_{N}\right|}^{2}, & & \text{for}\;\zeta \in \gamma_{\zeta }^{*}\setminus \left\{s_{N},\bar{s_{N}}\right\},\\ & \text{Re}\;\left(\Phi_{N}\left(\omega \right)-\Phi_{N}\left(s_{N}\right)\right)\geq C_{2}{\left|\omega -s_{N}\right|}^{2}, & & \text{for}\;\omega \in \gamma_{\omega }^{*}\setminus \left\{s_{N},\bar{s_{N}}\right\}, \end{array}$$

for a certain $C_{2}>0$. Therefore, the left‐most term in (218) is, in absolute value,

(219) $$\begin{array}{c} \leq C_{3}{\;\int \int \;}_{{\left|x\right|}^{2}+{\left|y\right|}^{2}\leq \varepsilon^{2}}\frac{e^{-4C_{2}N\left(x^{2}+y^{2}\right)}}{\sqrt{x^{2}+y^{2}}}dxdy=2\pi C_{3}\int_{0}^{\varepsilon }e^{-4C_{2}Nr^{2}}dr\leq C_{4}N^{-\frac{1}{2}} \end{array}$$

for certain $C_{3},C_{4}>0$ and for all large enough *N*. Therefore,

$$\begin{array}{c} I\left(x_{N},y_{N};H\right)-\frac{1}{2\pi i}\int_{\bar{s_{N}}}^{s_{N}}H\left(\zeta ,\zeta \right)d\zeta =O\left(N^{-1/2}\right),\;\text{as}\;N\to +\infty , \end{array}$$

which give (133).▪

### 11.2 The case ξ=0 and η<0

Let us briefly recall first the situation for $(\xi^{\prime },\eta^{\prime })\in L$, such that $\eta^{\prime }<\frac{\xi^{\prime }}{2}<0$. In this case, the set $N_{\Phi }$ contains four curves emanating from *s*: three of these curves, namely, Γ_1_, Γ_2_, and Γ_3_, lie in $\text{int}(\bar{\Sigma_{1}\cup \Sigma_{\alpha }})$, the other curve Γ_4_ intersects once $\bar{\Sigma_{1}\cup \Sigma_{\alpha }}$. Denote $p_{j}$ for the intersection of $\Gamma_{j}$ with R, and recall that the ordering for Γ_1_, Γ_2_, and Γ_3_ is such that $p_{1}<p_{2}<p_{3}$.

**FIGURE 17:**
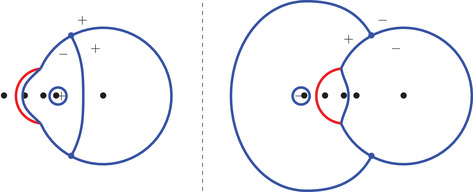
The sets $N_{\Phi }$ (left) and $N_{\Psi }$ (right) are represented in blue, and $\Sigma_{\alpha }$ in red. The parameters are (α,ξ,η)=(0.4,0,−0.75), and Σ_1_ is a subset of both $N_{\Phi }$ and $N_{\Psi }$. The signs of Re(Φ−Φ(s)) and Re(Ψ−Ψ(s)) are indicated with ±. In each figure, the black dots represent 0, αc, $\alpha c^{-1}$, *c*, and $c^{-1}$ and the blue dots are *s* and $\bar{s}$

As $\left(\xi^{\prime },\eta^{\prime }\right)\to \left(0,\eta \right)$ with η<0 (see Figures 12 and 17 (left)), we know from Proposition 4 that $s(\xi^{\prime },\eta^{\prime };\alpha )$ tends to a point s=s(0,η;α) lying on Σ_1_. In this limit, both Γ_3_ and Γ_4_ tend to the arc

$$\begin{array}{c} \Sigma_{s}:=\left\{c^{-1}+R_{1}e^{it}:-\arg s\leq t\leq \arg s\right\}\subset \Sigma_{1}, \end{array}$$

and a part of Γ_1_ tends to $\Sigma_{1}\setminus \Sigma_{s}$. Thus, the case ξ=0 gives less freedom for the contour deformations and the saddle point analysis is more involved. To handle this case, we need information about both $N_{\Phi }$ and $N_{\Psi }$, where

$$\begin{array}{c} N_{\Psi }:=\left\{\zeta \in C:\text{Re}\;\Psi \left(\zeta \right)=\Psi \left(s\right)\right\}. \end{array}$$

The sets $N_{\Phi }$ and $N_{\Psi }$ are represented in Figure 17 for a particular choice of the parameters. We have the following.

Lemma 14 For ξ=0, we have $\Sigma_{1}\subset N_{\Phi }$ and $\Sigma_{1}\subset N_{\Psi }$.

**FIGURE 18:**
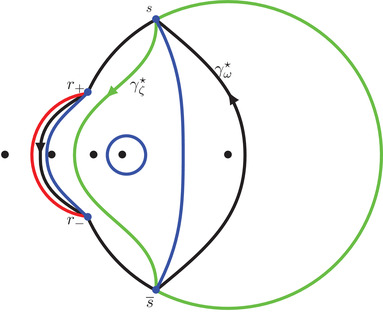
The contours $\gamma_{\zeta }^{*}$ (green) and $\gamma_{\omega }^{*}$ (black), with (α,ξ,η)=(0.4,0,−0.75)

Because Reϕ(ζ)=0 for $\zeta \in \Sigma_{1}$, by Definition 7 and (205) we have

$$\begin{array}{c} \text{Re}\;\Phi \left(\zeta \right)=\text{Re}\;\Psi \left(\zeta \right)=-\frac{\eta }{2}\text{Re}\;f_{1}\left(\zeta \right), \end{array}$$

and by Lemma 11 this expression is constant for $\zeta \in \Sigma_{1}$.▪

We choose $\gamma_{\zeta }^{*}$ and $\gamma_{\omega }^{*}$ as follows (see also Figure 18):

- $\gamma_{\omega }^{*}\subset \bar{\text{int}(\bar{\Sigma_{\alpha }\cup \Sigma_{1}})}$, is such that $\left(\Sigma_{1}\setminus \Sigma_{s}\right)\subset \gamma_{\omega }^{*}$, surrounds αc and $\alpha c^{-1}$, and it satisfies
  $$\begin{array}{c} \text{Re}\;\Phi \left(\omega \right)>\text{Re}\;\Phi \left(s\right),\;\omega \in \gamma_{\omega }^{*}\setminus \left(\bar{\Sigma_{1}\setminus \Sigma_{s}}\right), \end{array}$$
- $\gamma_{\zeta }^{*}\subset \bar{\text{int}(\gamma_{1})}$, is such that $\Sigma_{s}\subset \gamma_{\zeta }^{*}$, surrounds *c* and $c^{-1}$, and it satisfies
  $$\begin{array}{c} \text{Re}\;\Phi \left(\zeta \right)<\text{Re}\;\Phi \left(s\right),\;\zeta \in \gamma_{\zeta }^{*}\setminus \Sigma_{s}. \end{array}$$

Let $\{{\left(x_{N},y_{N}\right\}}_{N\geq 1}$ be a sequence satisfying (38) with $\left(\xi ,\eta \right)\in L_{\alpha }\cap \left\{\eta <\frac{\xi }{2}=0\right\}$, and define $\xi_{N}:=x_{N}/N-1$ and $\eta_{N}:=y_{N}/N-1$. By Lemma 7, we can (and do) assume without loss of generality that $\left(\xi_{N},\eta_{N}\right)\in L_{\alpha }$ satisfies $\eta_{N}<0$ and $\xi_{N}=0$ for all *N*. The proof of Proposition 18 still goes through with the above choice of $\gamma_{\zeta }^{*}$ and $\gamma_{\omega }^{*}$, and as in (217) we obtain

(220) $$I\left(x_{N},y_{N};H\right)-\frac{1}{2\pi i}\int_{\bar{s}}^{s}H\left(\zeta ,\zeta \right)d\zeta =\frac{1}{{\left(2\pi i\right)}^{2}}\int_{\gamma_{\zeta }^{*}}d\zeta \int_{\gamma_{\omega }^{*}}\frac{d\omega }{\omega -\zeta }H\left(\omega ,\zeta \right){\tilde{R}}^{T,a}\left(\omega ,\zeta \right)e^{2N(\Phi (\zeta )-\Phi (\omega ))},$$

where $s=s(\xi_{N},\eta_{N};\alpha )$, $\Phi \left(\zeta \right)=\Phi \left(\zeta ;\xi_{N},\eta_{N}\right)$, and the contours $\gamma_{\zeta }^{*}$ and $\gamma_{\omega }^{*}$ depend on *N*. We also take the + boundary value in (220) whenever $\omega \in \gamma_{1}$. Because ReΦ(ζ)=ReΦ(s) for all $\zeta \in \Sigma_{s}$ and ReΦ(ω)=ReΦ(s) for all $\omega \in \bar{\Sigma_{1}\setminus \Sigma_{s}}$, we need additional deformation of contours.

We first treat the contour deformations in the ζ‐variable. Recall the definition (212) of ${\tilde{R}}^{T,a}$. For $\zeta \in \Sigma_{s}$, we use $\Phi_{+}\left(\zeta \right)=\Psi_{-}\left(\zeta \right)$ and the jumps for *T* (184) to obtain

(221) $$\begin{array}{c} e^{2N\Phi (\zeta )}T\left(\zeta \right)\left(\begin{array}{c} 1\\ 0 \end{array}\right)=e^{2N(\Phi_{+}\left(\zeta \right)-2\phi_{+}\left(\zeta \right))}T_{+}\left(\zeta \right)\left(\begin{array}{c} 0\\ 1 \end{array}\right)-e^{2N\Psi_{-}\left(\zeta \right)}T_{-}\left(\zeta \right)\left(\begin{array}{c} 0\\ 1 \end{array}\right). \end{array}$$

**FIGURE 19:**
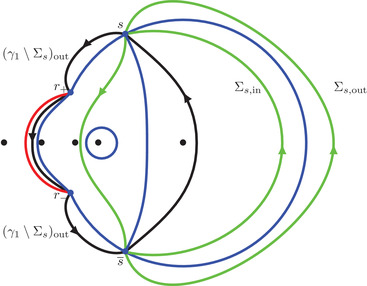
The further contour deformations we need to consider to handle the case ξ=0 and η<0

We substitute (221) in (220), and then split the integral over $\Sigma_{s}\subset \gamma_{\zeta }$ in (220) into two parts. For the second term in (221), the contour $\Sigma_{s}$ is deformed outward to $\Sigma_{s,\mathrm{out}}$, see Figure 19. Because $\Psi_{\pm }\left(\zeta \right)=\Phi_{\mp }\left(\zeta \right)$ for $\zeta \in \Sigma_{1}$, and because $\Sigma_{1}\subset N_{\Phi }\cap N_{\psi }$, the signs of

$$\begin{array}{c} \text{Re}\;\left(\Phi \left(\zeta +\varepsilon \left(\zeta -c^{-1}\right)\right)-\Phi \left(s\right)\right)\;\;\text{and}\;\;\text{Re}\;\left(\Psi \left(\zeta -\varepsilon \left(\zeta -c^{-1}\right)\right))-\Psi \left(s\right)\right) \end{array}$$

are different for all $\zeta \in \Sigma_{1}$, provided ε=ε(ζ)∈R is small enough (ε nonnecessarily positive), see also the signs around Σ_1_ in Figure 17. In particular, we have ReΨ(ζ)<ReΨ(s) for $\zeta \in \Sigma_{s,\mathrm{out}}$. For the first term in (221), the dominant part is $e^{2N(\Phi_{+}\left(\zeta \right)-2\phi_{+}\left(\zeta \right))}$, and by Definition 7, we have Ψ=Φ−2ϕ. Therefore, we deform $\Sigma_{s}$ inward to $\Sigma_{s,\mathrm{in}}$, and this contour is chosen such that ReΨ(ζ)<ReΨ(s) for $\zeta \in \Sigma_{s,\mathrm{in}}$, see Figure 19.

In the ω‐variable, we simply analytically continue the integrand and deform $\Sigma_{1}\setminus \Sigma_{s}$ outward to ${\left(\gamma_{1}\setminus \Sigma_{s}\right)}_{\mathrm{ext}}$, see Figure 19. This contour is chosen such that ReΨ(ω)>ReΨ(s) for $\omega \in {\left(\gamma_{1}\setminus \Sigma_{s}\right)}_{\mathrm{ext}}$. Because $\Phi_{+}\left(\omega \right)=\Psi_{-}\left(\omega \right)$ on Σ_1_, the exponential factor of the integrand is $e^{-2N\Psi (\omega )}$ there. Also, for $\omega \in \Sigma_{1}\setminus \Sigma_{s}$, by (184) we have

(222) $$\begin{array}{c} \left(\begin{array}{cc} 1 & 0 \end{array}\right)T_{+}^{-1}\left(\omega \right)=\left(\begin{array}{cc} e^{-4N\phi_{-}\left(\omega \right)} & -1 \end{array}\right)T_{-1}^{-1}\left(\omega \right), \end{array}$$

and we know from Lemma 9 that $e^{-4N\phi (\omega )}$ remains bounded for $\omega \in {\left(\gamma_{1}\setminus \Sigma_{s}\right)}_{\mathrm{ext}}$.

The result of the above deformations is that the integrand is uniformally exponentially small on the contours, as long as ζ stays away from $s,\bar{s}$, and that ω stays away from $s,\bar{s},r_{+},r_{-}$. By a similar analysis as the one done in (219), we show that the contribution to (220) when ζ and ω are close to *s* or $\bar{s}$ is $O(N^{-\frac{1}{2}})$ as N→+∞. When ω is close to $r_{\pm }$, we know by Proposition 15 that $T^{-1}\left(\omega \right)=O\left(N^{1/6}\right)$. Because $\Phi^{\prime }\left(r_{\pm }\right)\neq 0\neq \Psi^{\prime }\left(r_{\pm }\right)$, the contribution to (220) when ζ is close to *s* or $\bar{s}$ and simultaneously ω close to $r_{+}$ or $r_{-}$ is

$$\begin{array}{c} \leq C_{1}N^{\frac{1}{6}}{\;\int \int \;}_{{\left|x\right|}^{2}+{\left|y\right|}^{2}\leq \varepsilon^{2}}e^{-C_{2}N(|x|+y^{2})}dxdy\leq C_{3}N^{-\frac{17}{6}} \end{array}$$

for certain constant $C_{1},C_{2},C_{3}>0$ and all large enough *N*. In particular, this proves (133).

### 11.3 The case ξ=0 and η=0

At the center of the hexagon, we have $s=s\left(0,0;\alpha \right)=r_{+}$, $\bar{s}=r_{-}$, and Φ=−Ψ=ϕ (see also Definition 7). The sets $N_{\Phi }$ and $N_{\Psi }$ are then given by Lemma 9:

$$\begin{array}{c} N_{\Phi }=N_{\Psi }=N_{\phi }=\Sigma_{0}\cup \Sigma_{\alpha }\cup \Sigma_{1}. \end{array}$$

Note that for $\left(\xi^{\prime },\eta^{\prime }\right)=\left(0,\eta^{\prime }\right)\in L_{\alpha }$ with $\eta^{\prime }<0$, part of contour $\gamma_{\omega }^{*}$ lies in the region $\text{int}(\bar{\Sigma_{\alpha }\cup \Gamma_{1}})$, see Figures 17 and 19. As $\eta^{\prime }\to \eta =0$, Γ_1_ tends to $\Sigma_{\alpha }$, so we need additional contour deformations to handle this case. Consider the contours $\gamma_{\zeta }^{*}:=\gamma_{1}$ and $\gamma_{\omega }^{*}=\gamma_{\alpha }$. By Lemma 9, we have

$$\begin{array}{cccc} & \text{Re}\;\Phi \left(\omega \right)>0,\;\text{for}\;\omega \in \gamma_{\alpha }\setminus \bar{\Sigma_{\alpha }} & & \text{Re}\;\Phi \left(\omega \right)=0,\;\text{for}\;\omega \in \Sigma_{\alpha },\\ & \text{Re}\;\Phi \left(\zeta \right)<0,\;\text{for}\;\zeta \in \gamma_{1}\setminus \bar{\Sigma_{1}} & & \text{Re}\;\Phi \left(\zeta \right)=0,\;\text{for}\;\zeta \in \Sigma_{1}. \end{array}$$

For simplicity, we consider the sequence ${\left\{\left(x_{N},y_{N}\right)=\left(N,N\right)\right\}}_{N\geq 1}$, so that $\xi_{N}:=x_{N}/N-1=0$ and $\eta_{N}:=y_{N}/N-1=0$ for all *N*. In the same way as done in Proposition 18, we find

(223) $$I\left(x_{N},y_{N};H\right)-\frac{1}{2\pi i}\int_{r_{-}}^{r_{+}}H\left(\zeta ,\zeta \right)d\zeta =\frac{1}{{\left(2\pi i\right)}^{2}}\int_{\gamma_{\zeta }^{*}}d\zeta \int_{\gamma_{\omega }^{*}}\frac{d\omega }{\omega -\zeta }H\left(\omega ,\zeta \right){\tilde{R}}^{T,a}\left(\omega ,\zeta \right)e^{2N(\Phi (\zeta )-\Phi (\omega ))},$$

and we take the + boundary value in (223) whenever $\omega \in \Sigma_{\alpha }$.

For $\zeta \in \Sigma_{1}$, we use (221) to split the integrand into two parts, and again we deform the integral associated to the first term slightly inward, and the other one slightly outward. As a result, both deformed integrals have exponentially decaying integrands.

For $\omega \in \Sigma_{\alpha }$, ${\tilde{R}}^{T,a}\left(\omega ,\zeta \right)$ is given by the second line of (212), and thus the dominant ω‐part in the integrand is

$$\begin{array}{c} e^{-2N\Phi (\omega )}\left(\begin{array}{cc} 1 & -e^{4N\phi (\omega )} \end{array}\right)T^{-1}\left(\omega \right)=e^{-2N\phi (\omega )}\left(\begin{array}{cc} 1 & 0 \end{array}\right)T^{-1}\left(\omega \right)-e^{2N\phi (\omega )}\left(\begin{array}{cc} 0 & 1 \end{array}\right)T^{-1}\left(\omega \right). \end{array}$$

For the first term, we deform $\Sigma_{\alpha }$ outward so that Reϕ(ω)>0, and for the first term, we deform $\Sigma_{\alpha }$ inward so that Reϕ(ω)<0.

On the deformed contours, the integrand is uniformly exponentially small, as long as ζ and ω are bounded away from $r_{+}$ and $r_{-}$. For ζ and ω close to $r_{\pm }$, by Proposition 15 we have $T\left(\zeta \right)=O\left(N^{1/6}\right)$ and $T^{-1}\left(\omega \right)=O\left(N^{1/6}\right)$. The contribution to (223) when ζ and ω are close to $r_{+}$ and $r_{-}$ is thus bounded by

$$\begin{array}{c} \leq C_{1}N^{\frac{1}{3}}{\;\int \int \;}_{{\left|x\right|}^{2}+{\left|y\right|}^{2}\leq \varepsilon^{2}}\frac{e^{-C_{2}N\left(x^{2}+y^{2}\right)}}{\sqrt{x^{2}+y^{2}}}dxdy\leq C_{3}N^{-\frac{1}{6}} \end{array}$$

for certain $C_{1},C_{2},C_{3}>0$ and for all large enough *N*. This finishes the proof of Proposition 11.

## Footnotes

1 The quantities N,M, and *L* in the notation of Ref. 20 are equal to *N*, *N*, and 4*N* in our notation.
